# Reducing the biases of the conventional meta-analysis of correlations

**DOI:** 10.1017/rsm.2024.5

**Published:** 2025-04-01

**Authors:** T. D. Stanley, Hristos Doucouliagos, Tomas Havranek

**Affiliations:** 1 Department of Economics, Deakin University, Victoria, Australia; 2 Meta-Research Innovation Center at Stanford, Stanford, CA, USA; 3 Institute of Economic Studies, Faculty of Social Sciences, Charles University, Prague, Czechia; 4 Centre for Economic Policy Research, London, UK

**Keywords:** correlations, meta-analysis, publication selection bias, small-sample bias

## Abstract

Conventional meta-analyses (both fixed and random effects) of correlations are biased due to the mechanical relationship between the estimated correlation and its standard error. Simulations that are closely calibrated to match actual research conditions widely seen across correlational studies in psychology corroborate these biases and suggest two solutions: UWLS_+3_ and HS. UWLS_+3_ is a simple inverse-variance weighted average (the unrestricted weighted least squares) that adjusts the degrees of freedom and thereby reduces small-sample bias to scientific negligibility. UWLS_+3_ as well as the Hunter and Schmidt approach (HS) are less biased than conventional random-effects estimates of correlations and Fisher’s *z*, whether or not there is publication selection bias. However, publication selection bias remains a ubiquitous source of bias and false-positive findings. Despite the relationship between the estimated correlation and its standard error in the absence of selective reporting, the precision-effect test/precision-effect estimate with standard error (PET-PEESE) nearly eradicates publication selection bias. Surprisingly, PET-PEESE keeps the rate of false positives (i.e., type I errors) within their nominal levels under the typical conditions widely seen across psychological research whether there is publication selection bias, or not.

## Highlights

### What is already known?

- Dozens, perhaps hundreds, of meta-analyses of correlations are conducted each year.
- It has only recently been shown that all inverse-variance weighted meta-analyses of correlations are biased.^2^

### What is new?

- We investigate the statistical properties of alternative meta-analysis estimators of the population correlation coefficient with simulations that closely match typical research conditions widely seen across correlational studies in psychology with and without publication selection bias.
- We explore a novel correction, UWLS_+3,_ along with an often-neglected approach of Hunter and Schmidt (HS). Both reduce these small-sample biases to scientific negligibility.
- UWLS_+3_ is the unrestricted weighted least squares weighted average that adjusts degrees of freedom, and the HS approach uses the sample size as the weight. Both effectively eliminate small-sample biases and are less biased than random effects calculated on correlations or Fisher’s *z* whether there is publication selection bias or not.
- Despite the mechanical relationship between estimated correlations and their standard errors, precision-effect test/precision-effect estimate with standard error (PET-PEESE) effectively removes publication selection bias under the typical research conditions widely found across correlational studies in psychology.

### Potential impact for RSM readers outside the authors’ field

These meta-analysis methods apply widely to all disciplines where one wishes to conduct a systematic review of correlations.

## 1 Introduction

Correlations are widely used to summarize psychological research via inverse-variance weighted meta-analysis although, by conventional definitions, the variance (and standard error [SE]) of correlations is a function of the correlation estimate itself. What has yet to be fully recognized is that this dependence of the variance on the size of the correlation causes fixed and random-effects meta-analysis of correlations and partial correlations to be biased.^1^ ^–^ ^2^ The conventional approach to this dependence is to employ the Fisher’s *z* transformation,^3^ as its SE is independent of the estimate of *z*.^i^ Yet, many meta-analyses of simple, untransformed correlations are routinely conducted in psychology. For example, a survey found that a majority of the meta-analyses published in the *Psychological Bulletin* (108 of 200) concerned correlations. Within these 108 meta-analyses of correlations, 84.3% did *not* use the Fisher’s *z* transformation, but rather, the simple untransformed correlations.^4^

We follow previous studies which found that conventional meta-analyses (i.e., inverse-variance weighted averages, fixed and random effects, without additional corrections) of bivariate and partial correlations are biased because sample correlations are inversely correlated with their variances.^1^ ^–^ ^2^ Fortunately, these biases are small-sample biases. A new estimator, UWLS_+3_, is introduced below that reduces these biases to scientific negligibility by making a simple adjustment to the degrees of freedom. However, past studies assumed that the sample sizes were constant across all studies within a meta-analysis, there was no excess heterogeneity, and no publication selection bias (PSB). While these assumptions were necessary to isolate and to identify the small-sample bias caused by a correlation’s mechanical inverse correlation with its own SE, none of these conditions hold, even approximately, for the majority of meta-analyses of social science research. Relaxing these assumptions constitutes our main contribution to this stream of research.

The range of sample sizes synthesized by the typical meta-analysis is many times its median value. Thus, at least some studies in the majority of meta-analyses will be sufficiently large to reduce a correlation’s small-sample bias to practical negligibility. Second, although not every area of research selects for statistical significance and thereby produces PSB, it is rare when PSB can be ruled out *a priori*. When present, PSB can be substantial, creating high rates of false positives in conventional meta-analyses.^5^ Lastly, heterogeneity among psychological studies is rather large: *I* ^2^ = 74%, tau > .3*d*.^4^ In this study, we show that a new small-sample correction, UWLS_+3,_ and the old but often-overlooked Hunter and Schmidt (HS) approach^6^ ^–^ ^7^ reduce meta-analysis bias to rounding errors and investigate whether conventional meta-analysis (uncorrected inverse-variance weighted averages) will still be biased when there is a wide range of sample sizes and heterogeneity, with and without accompanying selection for statistical significance. In short, conventional, inverse-variance weighted meta-analyses are still biased under typical research conditions seen in psychology. However, we do not stop there. We also identify those meta-analyses methods that have no notable biases with or without PBS as well as those that are able to maintain their nominal type I errors (that is, those that do not have inflated rates of false positives) even with publication bias.

## 2 Correlation and its variances

The conventional formula for the Pearson (bivariate) correlation coefficient, *r*, is:
(1)

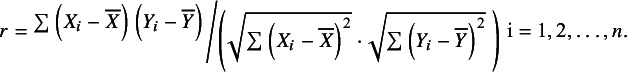



When testing whether the association between *X* and *Y* is statistically significant (i.e., H_0_: *ρ* = 0), *r*’s variance is:
(2)

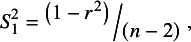

 and 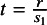 is the corresponding conventional test statistic. Correlation’s *t*-value is also equal to the *t*-value for the slope coefficient from the simple linear bivariate regression between *X* and *Y*. See Stanley et al.^2^ for numerical-analytic proof.

In contrast, conventional meta-analysis uses a different variance for correlations:^3^ ^,^ ^6^ ^,^ ^7^

(3)

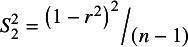




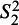
 is often considered the “correct” variance formula for a correlation when conducting meta-analyses.^1^ ^,^ ^3^ ^,^ ^6^ Note that the differences between these variance formulae are: 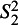 squares 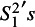 numerator, 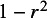 , and 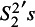 degrees of freedom, 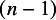 , are one fewer. Because −1 ≤ *r* ≤ 1 and 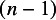 > 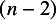 , 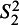 < 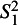 for all sample sizes and 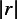 ≠ {0 or 1}. Simulations reported in Table 1, below, establish that using 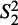 causes conventional meta-analyses to be twice as biased as those which use 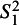 . These results corroborate prior findings.^1^ ^,^ ^2^ The main reason is that squaring the numerator reinforces the unwelcome mechanical relationship between the estimated correlation and its variance. Despite being the “correct” variance formula, 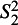 is less suitable than 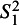 to be used in the weighting of sample correlations for meta-analysis.

**Table 1:** Meta-analyses of correlations (RE and UWLS) using different formulas for the correlation variance

Design			Bias				Coverage				RMSE			
Het/PSB	ρ	*I* ^2^	RE_2_	RE_1_	UWLS_2_	UWLS_1_	RE_2_	RE_1_	UWLS_2_	UWLS_1_	RE_2_	RE_1_	UWLS_2_	UWLS_1_
None	.447	.1058	.0097	.0042	.0103	.0042	.8595	.9572	.8362	.8925	.0142	.0110	.0147	.0110
None	.243	.1069	.0065	.0028	.0069	.0030	.9261	.9562	.9177	.9318	.0139	.0123	.0140	.0123
None	.110	.1079	.0028	.0010	.0030	.0011	.9530	.9603	.9483	.9493	.0132	.0126	.0131	.0125
*Average*			.0063	.0027	.0067	.0028	.9129	.9579	.9007	.9245	.0138	.0120	.0139	.0120
Het	.447	.6023	.0075	.0007	.0197	.0057	.8991	.9357	.7676	.8960	.0192	.0173	.0254	.0162
Het	.243	.6491	.0036	−.0009	.0138	.0039	.9247	.9331	.8928	.9377	.0228	.0216	.0244	.0192
Het	.110	.6638	.0016	−.0006	.0068	.0019	.9276	.9308	.9404	.9511	.0240	.0229	.0225	.0201
*Average*			.0042	−.0003	.0135	.0038	.9171	.9332	.8669	.9283	.0220	.0206	.0241	.0185
PSB	.447	.5668	.0190	.0113	.0260	.0124	.7573	.8686	.5988	.8019	.0250	.0194	.0301	.0190
PSB	.243	.6171	.0527	.0451	.0478	.0363	.2305	.3304	.2846	.4439	.0562	.0488	.0510	.0400
PSB	.110	.6879	.0955	.0894	.0819	.0725	.0187	.0226	.0368	.0560	.0982	.0919	.0842	.0748
*Average*			.0557	.0486	.0519	.0404	.3355	.4072	.3067	.4339	.0598	.0534	.0551	.0446
*Het and PSB average*			.0300	.0242	.0327	.0221	.6263	.6702	.5868	.6811	.0409	.0370	.0396	.0316

*Note*: *HET/PSB* describes different assumed conditions. With PSB, the simulations force both heterogeneity and 50% of the study results to be selected for statistical significance that is publication bias, Het assumes only heterogeneity, and None allows neither. *ρ* is the “true” population correlation. *Bias* is the difference between the meta-analysis estimate calculated from 50 estimated correlation coefficients and averaged across 10,000 replications. *RMSE* is the square root of the mean squared error. *Coverage* is the proportion of 10,000 meta-analyses’ 95% confidence intervals that contain *ρ*. *RE* is the random-effect’s estimate of the mean, and *UWLS* is the unrestricted weighted least squares’ estimate of the mean. The subscripts (1 and 2) refer to the use of either correlation variance, 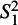 , from Equation (2) or 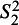 from Equation (3) to calculate UWLS’ and RE’s weighted averages. *I* ^**2**^ is a relative measure of heterogeneity.

Finally, there are different ways to calculate correlations. Following Gustafson^8^ and Fisher,^9^ Stanley et al.^2^ demonstrated that Equation (4) gives the exact same values for estimated correlations as the more conventional correlation formula, Equation (1).
(4)

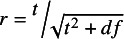

where 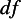 *= n –* 2. *t* is the conventional *t*-test for the statistical significance of the slope coefficient of a bivariate regression or, equivalently, the *t*-value of correlations using 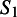 . This *t*-formula for correlations, Equation (4), is central to a new small-sample correction, UWLS_+3_.

## 3 Meta-analysis of correlations

Random-effects (REs) weighted averages are, by far, the most employed meta-analysis approach used to systematically review and summarize correlations across studies in a given area of research in psychology. RE is, thereby, the conventional standard upon which to establish the bias of the conventional meta-analyses of correlations—see Table 1. RE serves as the baseline from which to evaluate the statistical performance of alternative meta-analysis methods.

### 3.1 The unrestricted weighted least squares (UWLS) weighted average

The UWLS is an alternative simple weighted average that has statistical properties practically equivalent to RE under ideal conditions for RE and is notably superior if there is publication bias or if small-sample studies are more heterogeneous.^10^ ^–^ ^13^ Also, UWLS has been shown to be widely and notably superior to RE in most applications in psychology and medicine.^13^ ^,^ ^14^

UWLS is calculated from the simple meta-regression:
(5)

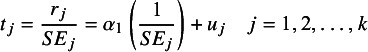

where *k* is the number of estimates contained in the meta-analysis, 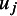 is the conventional regression error term, and 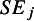 is the SE of the *j*th correlation calculated as the square root of either 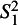 or 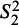 from their respective formulas; that is, Equation (2) or Equation (3). Without assuming the normality of 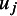 but merely that it is independently and identically distributed (i.e., 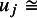 IID(0, 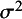 ), the Gauss–Markov Theorem proves that UWLS is unbiased and minimum variance or, more precisely, BLUE (best linear unbiased estimator).^15^ ^–^ ^16^ Any standard statistical software for regression analysis will automatically estimate UWLS (the slope coefficient, 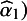 , its standard error, CI, and test statistics.

Stanley et al.^17^ offered a new correction, UWLS_+3_, for the small-sample biases of the conventional meta-analysis of *partial* correlations first identified in Stanley and Doucouliagos.^1^ Like the biases of the meta-analysis of partial correlations, Stanley et al. (Table 1)^2^ show that conventional RE’s small-sample biases are positive, can be of a notable magnitude for small samples, and are halved if 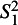 replaces the “correct” variance, 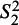 . Unfortunately, even with this change in variance, the small-sample biases can be larger than rounding error (.01). We seek to reduce further the biases of meta-analyses of correlations. A century ago, Fisher^18^ argued that what is true for correlations is also true for partial correlation:

Sampling distribution of the partial correlation obtained from *n* pairs of values, when one variable is eliminated, is the same as the random sampling distribution of a total correlation derived from (*n*-1) pairs. By mere repetition of the above reasoning it appears that when *s* variates are eliminated the effective size of the sample is diminished to (*n-s*). (Fisher, p. 330)^18^

Perhaps then, the reverse is also true: what is true for partial correlations is also true for correlations?

**Figure 1:**
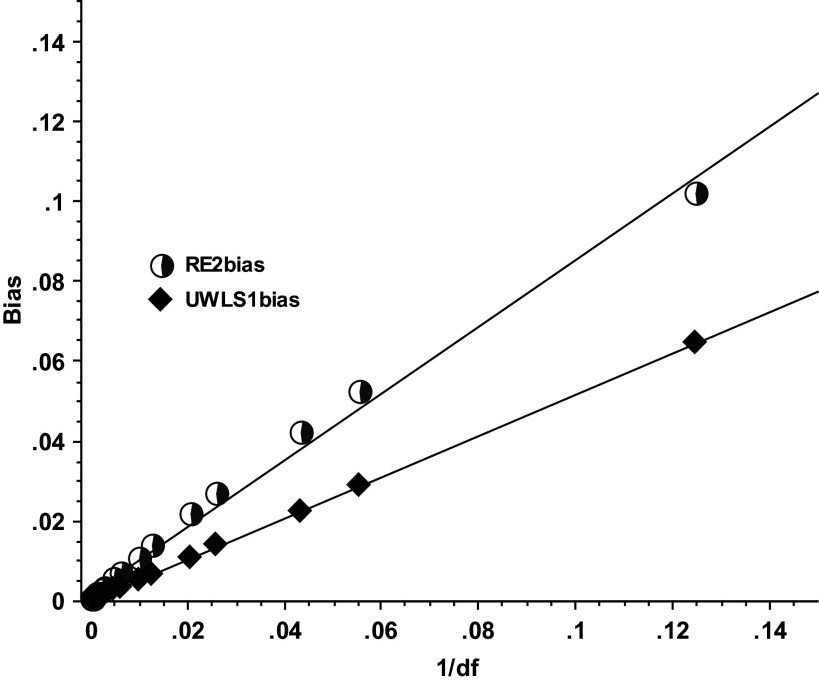
*Biases of random-effects (RE) and the unrestricted weighted least squares (UWLS)*. *RE2bias is RE’s bias across 10,000 replications that use the conventional MA variance*, 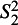 , *from Equation (3). UWLS1bias is UWLS’ bias across 10,000 replications that use* 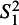 *from Equation (2).*

To address this question and to better understand the nature of the biases of the conventional meta-analysis of correlations, we first conduct a numerical analysis. To do so, we run simulation experiments of 10,000 replications, each doubling the sample size of the previous experiment (*n* = {10, 20, 40, 80, 160, 320, 640, 1280 & 25, 50, 100, 200, 400, 800, 1600, 2500}) for *ρ* = 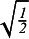 ; otherwise, these simulations use the same design as those reported in (Stanley, Doucouliagos, Maier and Bartos).^2^ Figure 1 plots conventional meta-analysis biases (i.e., RE’s bias using the “correct” variance, 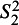 ) and UWLS’ biases using 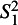 against the inverse of degrees of freedom (1/*df*). Figure 1 illustrates that the use of 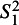 halves these biases, a doubling of the sample size also halves these biases, and the biases of UWLS are effectively an exact function of the inverse of degrees of freedom (1/*df*):

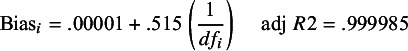




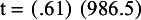

That is, numerical analysis reveals a near perfect inverse relationship of UWLS’ biases to degrees of freedom, 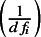 , explaining over 99.99% of the variation in UWLS’s bias (*R* ^2^ ≈ 99.999%) and leaving only a negligible random error. Note also that UWLS’s bias shrinks virtually to zero asymptotically (i.e., as *n* **→** ∞), 95% CI = (−0.000030; 0.000053). This near perfect fit demonstrates that these are small-sample biases and suggests that a modification to the degrees of freedom may correct these biases.

Figure 1 also reveals a very close relationship of RE’s bias with inverse *df*, although its fit is not nearly as close as UWLS’.^ii^ RE’s standard error of the estimate, which is the typical deviation of these biases from their predicted values, is 38 times larger than UWLS’. We focus on adjusting UWLS’ degrees of freedom (giving UWLS_+3_) rather than adjusting RE because we find that UWLS_+3_ has smaller biases and better statistical properties than adjusting RE.^17^ In part, this is due to the fact that RE must first estimate the heterogeneity variance before an estimate of mean effect can be calculated and thereby creates an additional source of variation and sampling error that UWLS does not have. Furthermore, any small-sample correction to RE is more biased than any of the alternative weighted averages in the presence of PSB just as RE is widely known to be more biased than UWLS when there is PSB.^10^ ^–^ ^12^ Below, we show that UWLS_+3_, can reduce these small-sample biases to scientific triviality.

As suggested by our numerical analysis, an adjustment to degrees of freedom may remove UWLS’ small-sample biases. Because Gustafson’s^8^ formula for a correlation, Equation (4), is itself a function of degrees of freedom, an adjustment to the degrees of freedom there might remove these small-sample biases? UWLS_+3_ is the unrestricted weighted least squares weighted average (i.e., Equation (5)) after three is added to the degrees of freedom in Equations (4) and (2) giving:
(6)

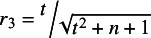



(7)

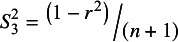



(8)

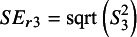



The *t*-values in Equation (6) are the *t*-values from the estimated bivariant regression slope coefficient or they may be equivalently calculated from the conventional *t*-value for correlations, *t* = 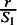 . Again, correlations calculated from Equation (4), with *df* = *n* − 2, produce identical correlation values as those calculated from conventional formulas for correlations.^2^ Note that that adding exactly three to the degrees of freedom is an arbitrary choice; adding four would further reduce the bias. But, as we have noted, once three are added, the remaining bias becomes trivial, so we prefer the least biased correction.

It is important to note that these small-sample corrections of correlations, *r*_3_ and *SEr_3_*, *should not* be applied to individual stand-alone correlations because it is widely known that individual correlation estimates, and partial correlation coefficients, are biased downward.^6^ ^,^ ^17^ ^,^ ^19^Applying this small-sample adjustment to stand-alone correlations would then only make a small downward bias worse. Rather, these transformations are merely an intermediate step in the calculations of meta-analysis weighted averages of correlations.

### 3.2 The HS approach to the meta-analysis of correlations

Hunter and Schmidt^6^ ^,^ ^7^ offered an alternative meta-analysis approach (HS), which they argued is superior to Fisher’s *z*.^iii^ HS uses the sample size, *n*, of each study as the weights. Thus, like Fisher’s *z*, HS avoids any dependence arising from the weights being dependent on the estimated correlations and thereby their sampling errors. The HS meta-analysis estimate of the mean correlation is:
(9)

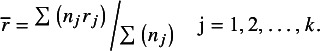



The variance of HS is not calculated as the conventional RE and FE meta-analysis by the inverse of the sum of the weights, but rather as:
(10)

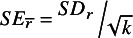

where the correlations’ standard deviation, 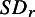 , is the square root of the weighted sum of squared deviations from the mean, 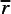 :^6^ ^,^ ^20^

(11)

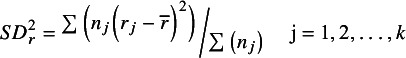



Table 2 compares the statistical properties of the resulting HS estimator to REz, UWLS_+3_, and precision-effect test/precision-effect estimate with standard error (PET-PEESE).

### 3.3 Fisher’s *z* transformation

An issue that has long been recognized by meta-analysts is that the SEs of correlations are a mathematical function of the correlation itself—recall Equations (2) and (3). This dependence is the source of the small-sample bias of the meta-analysis of partial correlations.^1^ Strictly speaking, the inverse-variance weights are no longer optimal and create bias. To circumvent this issue, meta-analysts often first transform correlations to Fisher’s *z*, calculate the random-effects estimate, then convert this RE estimate from terms of *z* back to a correlation.^3^ ^,^ ^iv^ Here, we call this *z*-transformed RE estimator, REz.

### 3.4 PET-PEESE model of publication selection bias

Publication selection bias (PSB), variously called: the “file drawer problem,” “publication bias,” “reporting bias,” “p-hacking,” and “questionable research practices” (QRP), has long been recognized by social scientists and medical researchers as a central problem for meta-analysis and empirical research in general. PSB has been offered as a leading explanation of the widely discussed “replication crisis,” and recent meta-research surveys have shown that PSB is the central suspect in the exaggeration of psychology’s typical reported effect sizes and statistical significance.^21^ ^–^ ^23^

PET-PEESE ranks among the best methods to accommodate and reduce PSB.^5^ ^,^ ^22^ ^,^ ^24^ ^,^ ^25^ PET-PEESE is calculated as the slope coefficient from one of two meta-regressions:
(12)

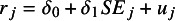



(13)

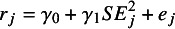

 using weighted least squares (WLS) with 1/ 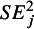 as the weights.^25^ If the regression coefficient, 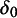 , is statistically significant (one-tail α = .10), then the estimate of 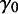 is PET-PEESE. Otherwise, the estimate of the regression coefficient, 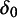 , is PET-PEESE.

PET-PEESE has been used in dozens of meta-analyses in psychology. For example, PET-PEESE anticipated the failure of ego depletion to replicate.^26^ ^,^ ^27^ Kvarven et al.^5^ conducted a systematic review of all pairs of preregistered multi-lab replications and meta-analysis. They compared RE, 3PSM (i.e., three-parameter selection model of publication bias), and PET-PEESE to the findings from these large-scale preregistered, multi-lab replications. On average, RE was three times larger than the corresponding replication result, bias = .26 *d* (Cohen’s *d*), and RE had a 100% “false-positive” rate.^5^ 3PSM was little better. In contrast, PET-PEESE’s bias, relative to these preregistered multi-lab replications, is only .051*d*, and PET’s false-positive rate is much lower than RE’s, especially so (9%) when Cohen’s^28^ probabilistic proof of a null effect defines “false positive.”^29^ ^,^ ^v^

Incidentally, PET-PEESE belongs to the same family of UWLS estimators as UWLS_+3_ along with other methods that progressively reduce publication bias: WAAP (weighted average of the adequately powered)^12^ and WILS (weighted and iterated least squares).^30^ ^,^ ^vi^ UWLS may be seen as a PET-PEESE meta-regression model that uses the same weights but does not include any independent variable: neither *SE* nor *SE* ^2^.

Table 2 reports simulations for PET-PEESE (PP) and a second version of PET-PEESE that regresses Fisher’s *z* on its *SE* or variance (PPz). PPz first converts correlations to Fisher’s *z*, regresses these *z*s using corresponding versions of Equations (12) and (13), and then transforms PPz back to a correlation. PPz avoids the correlation of *r* and its *SE* when there is no publication bias. Another alternative solution, which we do not simulate here, would be to use the square root of the inverse sample size as an instrument for the standard error in PET-PEESE.^31^ ^,^ ^32^ Among other things, the instrumental-variable PET-PEESE technique accounts for the mechanical correlation between *r* and its SE. See Irsova et al.^32^ for simulations of this instrumental-variable approach.

**Table 2:** RE_z_, UWLS_+3,_ HS, and PET-PEESE meta-analyses of correlations

Design: No heterogeneity or PSB														
Bias					Coverage					RMSE				
UWLS+_3_	REz	HS	PP	PPz	UWLS+_3_	REz	HS	PP	PPz	UWLS+_3_	REz	HS	PP	PPz
−.0002	.0013	−.0015	.0128	−.0002	.9537	.9588	.9449	.8674	.9488	.0100	.0101	.0101	.0198	.0154
−.0001	.0008	−.0010	.0083	−.0001	.9496	.9571	.9402	.9266	.9487	.0120	.0121	.0119	.0199	.0181
.0000	.0005	−.0004	.0033	−.0012	.9452	.9540	.9375	.9435	.9467	.0127	.0128	.0126	.0217	.0221
***.0001* *^a^***	***.0008***	***−.0010***	***.0081***	***−.0005***	***.9495***	***.9566***	***.9409***	***.9125***	***.9481***	***.0115***	***.0117***	***.0116***	***.0205***	***.0185***
*Type I error rate*					**.0233**	**.0185**	**.0254**	**.0248**	**.0257**	
*Design: Only heterogeneity*														
.0015	−.0009	−.0070	.0274	−.0004	.9547	.9368	.9365	.8170	.9718	.0153	.0173	.0166	.0343	.0211
.0010	−.0009	−.0050	.0192	−.0004	.9559	.9356	.9457	.9347	.9749	.0186	.0212	.0186	.0321	.0252
.0005	−.0005	−.0024	.0056	−.0066	.9571	.9349	.9498	.9684	.9777	.0200	.0226	.0192	.0350	.0366
***.0010***	***−.0008***	***−.0048***	***.0174***	***−.0025***	***.9559***	***.9358***	***.9440***	***.9067***	***.9748***	***.0180***	***.0204***	***.0181***	***.0338***	***.0276***
*Type I error rate*					**.0227**	**.0342**	**.0255**	**.0074**	**.0116**	
*Design: Both heterogeneity and 50% PSB*														
.0079	.0093	.0001	.0198	−.0076	.9190	.8985	.9463	.8912	.9598	.0165	.0184	.0143	.0284	.0217
.0341	.0444	.0274	.0141	−.0058	.5291	.3383	.6297	.9463	.9650	.0380	.0481	.0318	.0292	.0251
.0716	.0890	.0653	.0057	−.0214	.0657	.0202	.0861	.8260	.8632	.0739	.0914	.0677	.0611	.0643
***.0379***	***.0476***	***.0309***	***.0132***	***−.0116***	***.5046***	***.4190***	***.5540***	***.8878***	***.9293***	***.0428***	***.0526***	***.0379***	***.0396***	***.0370***
*Type I error rate*					**.9961**	**.9993**	**.9961**	**.0169**	**.0113**	
*Design: Averaged across all heterogeneity and PSB conditions*														
***.0194*** *^a^*	***.0242*** *^a^*	***.0179*** *^a^*	***.0153***	***−.0070***	***.7302***	***.6774***	***.7490***	***.8973***	***.9521***	***.0304***	***.0365***	***.0280***	***.0367***	***.0323***

*Note*: The design conditions in Table 2 are the same as in Table 1, where the three rows differ as *ρ* = {.447, .243, .110}. *ρ* is the “true” population correlation. *Bias* is the difference between the meta-analysis estimate calculated from 50 estimated correlation coefficients and averaged across 10,000 replications. *RMSE* is the square root of the mean squared error. *Coverage* is the proportion of 10,000 meta-analyses’ 95% confidence intervals that contain *ρ*. Type I errors, by definition, must assume that *ρ* = 0, and thereby only be reported once for each design condition. *UWLS+*_**3**_, as discussed in text, is the unrestricted weighted least squares meta-average with three additional degrees of freedom, *REz* is the random effects estimate of the mean correlation after being transformed back from Fisher’s *z*, *HS* is the Hunter and Schmidt approach, *PP* is PET-PEESE, and *PPz* is the PET-PEESE that uses the Fisher’s *z* transformation. Biases reported as “.0000” have absolute values < .00005. The fourth row in *bold italics* for each design is the average of the above three design conditions.

a Average biases are averaged across the absolute values of the biases.

### 3.5 An illustration

Eastwick et al. conducted a meta-analysis of the correlations of physical attractiveness and earning potential on men and women’s romantic evaluations.^33^ The research literature suggests that: “The attractiveness of the target affects men’s romantic evaluations more than women’s, and the earning prospects of the target affect women’s romantic evaluations more than men’s” (p. 627).^33^ This meta-analysis reported several random-effects estimates but focused on the gender differences and their moderators. For the sake of illustrating the methods discussed above, we focus on the correlation between the perceived earning potential of candidate men on women’s romantic evaluations. Conventional random-effects estimate the correlation of the earnings potential of the target on women’s romantic evaluations as: 0.128; 95% CI (0.092, 0.164), *k* = 73. Using REz does not notably affect these values: 0.127; 95% CI (0.092, 0.163). This is to be expected as the correlation is small, and small correlations have smaller small-sample biases. Furthermore, the sample sizes vary widely from 11 to over 7,000 with most studies having *n* > 100.

On the other hand, UWLS_+3_ reduces the RE estimate by over 60%: 0.050, 95% CI (0.022, 0.078). That is, UWLS_+3_ reduces a small correlation to a trivial one by Cohen’s benchmarks.^34^ It is important to note that Eastwick et al. *^33^* accept Cohen’s definition of “small” effect sizes (.1 ≤ *r* ≤ .3) and use it to characterize their central findings—(Eastwick et al., Abstract).^33^ UWLS_+3_ is calculated by first adjusting each correlation by Equation (6), giving 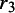 , then applying the simple UWLS regression, Equation (5), of *t*-values = 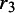 /SEr_3_ (DV) with precision, 1/SEr_3_, as the only explanatory variable and no constant—see Equations (6), (7), and (8) and the Supplement for the STATA code. HS produces virtually the same mean estimate as does UWLS_+3_: 0.047, 95% CI (0.014, 0.079).

The primary reason that HS and UWLS_+3_ notably reduces the effect size is likely publication selection bias. UWLS, in general, is widely known to reduce PSB more than corresponding random-effects, and the below simulations confirm that both HS and UWLS_+3_ are less biased than either RE or REz. HS is also less vulnerable to PSB because, like UWLS, its weights are not moderated by the additive heterogeneity variance, tau.^2^ However, these simulations also show that all weighted averages are notably biased when there is a small correlation, PSB, and notable heterogeneity, as we see here (tau = 0.128; *I* ^2^ = 85%).

**Figure 2:**
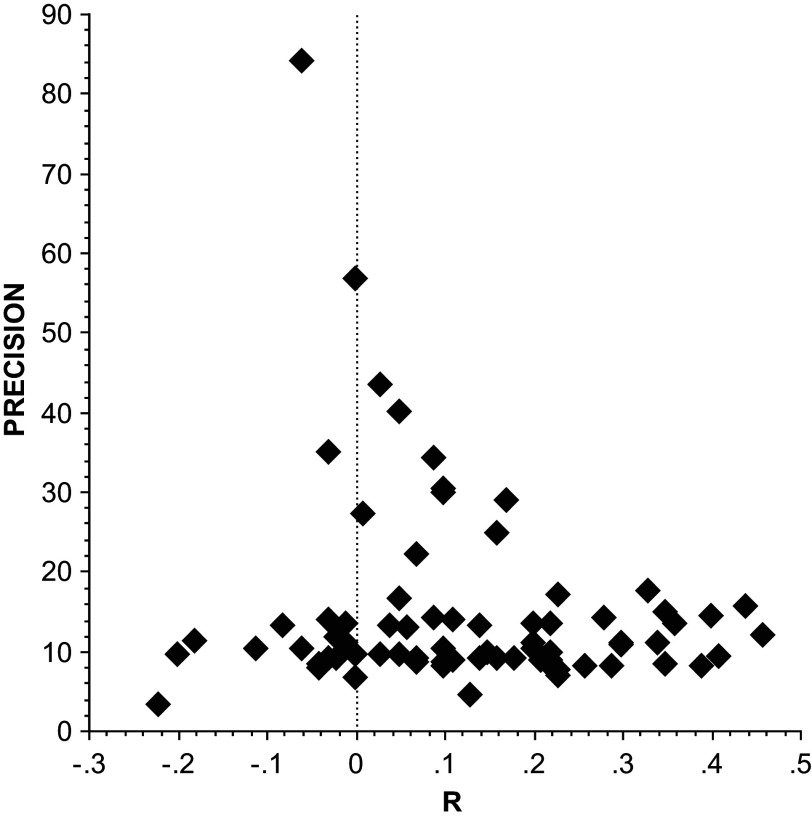
*A plot of the earnings-romance correlations, r, for women against their precision*, 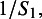 *on the vertical axis. Source*: Eastwick et al.^33^

Testing whether the coefficient on SE in Equation (12) is statistically significant is a test for PSB (the Egger test), also called the funnel-asymmetry test, or FAT.^25^ ^,^ ^31^ ^,^ ^35^ The estimated FAT-PET meta-regression, Equation (12), for these earnings-romance correlations and the associated Fisher’s *z* (Fz) are:
(14)

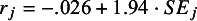




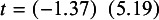



(15)

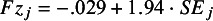




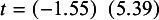

where the second lines report the *t*-values of the intercept (PET) and slope coefficients (FAT) in parentheses, and both meta-regressions use inverse variances as WLS weights. Also note that 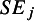 is different in Equations (14) and (15). For correlations, 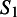 is its standard error, and Fisher’s *z* employs 1/sqrt(*n*−3) as its standard error. In both cases, PET fails to reject the null hypothesis that the earnings-romance correlation for women is zero (*t* = {−1.37; −1.55}; *p* > .05). In other words, once potential publication selection bias (or generally funnel asymmetry) is accommodated, no evidence of a positive earnings-romance correlation remains.^vii^ Also, both tests of the slope coefficients (FAT) are consistent with funnel asymmetry and, therefore, PSB (*t* = {5.17; 5.39}; *p* < .001) and that this size of this bias is quite large.^36^ ^,^ ^viii^ This funnel asymmetry can also be clearly seen in the funnel graph, see Figure 2.

Consistent with this interpretation, observe that the largest sample estimates are all quite small. For example, there are only two studies that are adequately powered (power ≥ 80%), when power is computed using UWLS_+3_ as the estimate of the population mean correlation. These two studies are at the top of the funnel (Figure 2) and have correlations = {−0.06, 0}; thus, the most reliable and informative studies in this area of research find no evidence of a positive correlation between perceived earning potential and women’s romantic inclinations. Considerations of power alone make the random-effects estimate dubious.^29^ Greater resilience to PBS is perhaps HS and UWLS_+3_’s most important property in application. We turn next to simulations that show this to be a general property of both UWLS_+3_ and HS.

## 4 Simulations

To better understand the statistical properties of the meta-analysis of correlations under research conditions commonly seen in psychology, we conduct Monte Carlo simulations. Unlike replications or other empirical analyses, simulations allow us to set and thereby know the exact ‘true’ (population) value, *ρ*, of the correlations investigated. To ensure that they reflect typical research conditions found across psychology, we closely calibrate our simulations design to match the key research dimensions found in correlational research. For this purpose, we employ 108 *Psychological Bulletin* meta-analyses of correlations reported in Stanley *et al*.^4^ These 108 meta-analyses jointly contain 5,891 pairs of estimated correlations and their standard errors, from which we can also calculate the sample sizes.

To generate estimated correlations for some variable of interest, 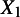 , we begin with the regression:
(16)

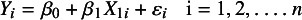



For simplicity, we assume that 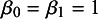 and that 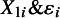 are independently, identically and normally distributed as *N*(0,1). In these simulations, 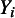 , is generated by Equation (16) after random and independent *N*(0,1) values are generated separately for 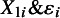 . Next, the simple, bivariate regression, Equation (16), is estimated, and the *t-*value of the estimated regression coefficient, 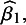 is calculated. 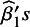 *t*-value is then converted to a correlation by Equation (4).

The median sample size is 95, which we round to 100, the 10th percentile uses a sample of 30, and 90th percentile is 424, which we round down to 400 so as not to exaggerate the likely precision of some studies in an area of research. Although a very small percentage of studies use thousands and tens of thousands of observations, to assume larger sample sizes risks underestimating the biases of the majority of meta-analysis of correlations. Recall that related simulation experiments where the sample sizes are fixed but repeatedly doubled reveal how all meta-analyses of correlations have small-sample biases which predictably disappear with larger sample sizes at a rate nearly exactly proportional to 1/df and df = *n*−2. Using these percentiles as our anchors, we fill in the remainder of the sample size distribution as, *n* = {30, 40, 50, 75, 100, 100, 125, 160, 200, 400}, to correspond to the sample size distribution observed across these 108 *Psychological Bulletin* meta-analyses.

Similarly, the values of the population correlation are set to correspond to the observed distribution of random-effects estimates reported in these same 108 meta-analyses. The median absolute value of these 108 REs is 0.232, which, for convenience, we approximate by the sqrt (1/17) = 0.243. The 10th percentile is 0.07, which we “round” up to sqrt (1/82) = 0.110. As shown in previous studies and as confirm below, small values of *ρ* produce practically no bias unless study results are selected for their statistical significance (i.e., publication selection bias). Thus, we make this small correlation a bit larger, intentionally. The 90th percentile of the RE distribution is 0.422, which we “round” up to sqrt (1/5) = 0.447. The 10th and 90th percentiles reflect a range of *ρ* values most likely seen in practice. However, as discussed in Section 3.1, all these values are likely an exaggerated reflection of the “true” population mean as RE is widely recognized to be upwardly biased in the presence of PSB, a condition we simulate, corroborate, and discuss further below. Thus, one should focus on the results of the more representative correlation effect sizes, 0.243 and 0.110, or consider the average across all three values of *ρ* reported in Tables 1 and 2 as “representative.”

For each correlational study, all data are generated from Equation (16), this regression is estimated, then 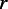 is calculated from Equation (4), 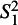 is calculated from Equation (2), and 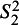 from Equation (3). Each of these steps is repeated 50 times to represent *one* meta-analysis.^ix^ From these 50 randomly generated estimated correlations, RE is calculated using the DerSimonian-Laird estimate of the heterogeneity variance, and both RE and UWLS weighted averages are calculated using both formulas for variance (Table 1). For each of 10,000 randomly generated meta-analysis, RE’s and UWLS’ biases, square roots of the mean squared errors (RMSE), and coverage rates are calculated. See the Supplement for the simulation code. To investigate whether the use of the “correct” variance, 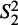 , continues to cause conventional meta-analysis weighted averages to have consistently larger biases than those employing 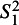 , Table 1 reports the results of these simulations using both versions of *r*’s variance—Equation (2) and Equation (3) for RE and UWLS. Prior studies showed that 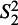 consistently produces twice the bias as 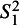 , but it remains an open question whether this result will remain under the above more representative research conditions found in psychology. Table 1 corroborates prior findings that 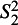 generates larger mean squared errors and inferior coverage (i.e., coverage rates that are often much different than their nominal 95% level).

For Table 2, we use the same simulation design to evaluate the statistical properties of alternative meta-analysis estimators of the mean effect: UWLS_+3_, HS, and Fisher’s *z*. Table 2 also reports type I error rates, which, by definition, assume that *ρ* = 0 and then counts how many tests of H_0_: *ρ* = 0, reject H_0_ in the positive direction (α = .05). Table 2 also investigates how well PET-PEESE accommodates PSB; it does surprisingly well.

In the heterogeneity conditions, labeled *Het* in Tables 1 and 2, we again rely on what was found to be the typical across these 108 meta-analyses, mean *I* ^2^ = 64.5%. Note that the typical heterogeneity reported in Tables 1 and 2, for the *Het* case nearly reproduces this level of relative heterogeneity. To do so, we assume that heterogeneity is weakly and inversely correlated with sample size; that is, normally distributed with standard deviations of *τ* = {.45, .45, .3, .3, .3, .3, .3, .3, .075, .075} as *n* = {30, 40, 50, 75, 100, 100, 125, 160, 200, 400}. Meta-research evidence shows that psychology’s heterogeneity is inversely correlated with sample size and simulations confirm that these values of heterogeneity produce the level of correlated heterogeneity observed across dozens of psychology meta-analyses.^13^ To generate random normal heterogeneity, we first convert each estimated correlation to Cohen’s *d*, add a random normal deviation with mean zero and standard deviations {.45, .45, .3, .3, .3, .3, .3, .3, .075, .075} and transform these Cohen’s *ds* back to correlations.^x^

In the PSB condition, we follow previous studies by assuming that exactly half of the results contained in a meta-analysis have been selected to be statistically significant, while the first random result produced by the other 50% is reported, as it is, statistically significant or not, and included in the meta-analysis.^10^ ^,^ ^12^ ^,^ ^22^ ^,^ ^25^ ^,^ ^37^ We do not mean to imply that all areas of psychology have such strong selection for statistical significance. Thus, we also report cases of no selection for statistical significance. Table 2 reports the average statistical results across simulations where there is 50% publication selection bias and where there is no selection for statistical significance. The average across heterogeneity (*Het*) and 50% PSB (*PSB*) is likely to better reflect typical areas of psychology, this average is reported in the last row of Table 2, labeled *PSB & Het Ave*.

The full details of how we generate 500,000 correlation studies from individual subject data, collectively containing 64 million subjects, are reported in the Supplement and found in previous studies.^2^ ^,^ ^17^ The central innovations in this paper relative to these recent simulation studies are: (i) the use of a distribution of sample sizes, rather than a single fixed sample size, (ii) the inclusion of heterogeneity typically seen in psychology, (iii) the infusion of 50% PSB, (iv) the investigation of different weighted averages, UWLS_+3_ and HS (Table 2), (v) the assessment of PET-PEESE for PSB also reported in Table 2, and (vi) a simulation context that follows what is known about actual meta-analyses of correlations in psychology.

## 5 Results and discussion

Table 1 confirms that the conventional meta-analysis formula of correlations’ variance,^3^ 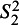 , should not be used in meta-analysis when an obvious and simple alternative is always available, 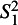 . In all cases, the biases, MSE and CIs are better when 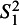 is used rather than 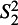 . Thus, by simply not squaring the numerator of correlation’s variance formula, the biases and MSEs of conventional meta-analysis are reduced, and the CIs notably improved. However, when there is no publication bias, the biases of conventional random effects are little more than rounding error (≤ .01). When there is notable selection for statistical significance (i.e., publication selection bias), the biases of all simple meta-analysis methods can be of scientific and practical consequence (> .05).^xi^ This is especially problematic for more than half of psychological research where effect sizes are small (see the smaller values of *ρ* = {.11; .243} in the above simulations). The publication bias of RE is especially pernicious when research synthesis is needed most: small correlations. For these, the bias of RE is likely to be as large as the true population correlation or nearly so, and RE is likely to falsely suggest a genuine effect where there is none (see Tables 1 and 2). With notable publication bias, conventional random-effects meta-analyses of 50 correlations are virtually certain to be falsely positive (i.e., to be statistically significant when the correlation is, in fact, zero)—see the type I error rates reported in Table 2.^xii^

Table 2 reports two further meta-analysis estimators, UWLS_+3_ and REz, shown by Stanley et al.^17^ and Stanley et al.,^2^ respectively, to outperform conventional unadjusted inverse-variance weighted meta-analyses of partial correlations and correlations. To these, we add the HS approach.^6^ ^,^ ^7^ Table 2’s simulations show that all three of these alternative estimators outperform conventional meta-analyses of correlations (RE) and reduce the small-sample biases to less than rounding error, unless, of course, there is notable publication selection bias. This remains the case even when meta-analyses have a typical distribution of sample sizes and heterogeneity, see the top two thirds of Table 2 and compare them to Table 1. However, as expected, all simple weighted averages, including HS, UWLS_+3_ and REz can have scientifically notable biases when there is 50% PSB.

There is a long-standing controversy regarding which approach is better: the Hedges–Olkin random-effects of Fisher’s z (REz) or the Hunter and Schmidt sample-size weighting of sample correlations (HS). Hunter and Schmidt^6^ claimed that their method was better than the random-effects conversion to Fisher’s *z* and recommended against the use the Fisher’s *z* transformation. Yet, most applications currently follow Borenstein et al.^3^ and calculate random-effects of Fisher’s *z*. Several studies have addressed this controversy,^38^ ^–^ ^41^ and the more recent ones^39^ ^,^ ^41^ generally find that HS is somewhat better. Our simulations find that both UWLS_+3_ and HS have better statistical properties than REz and thus agree with Hall and Brannick^39^ and Field.^41^ However, we also find that the differences are trivial under typical conditions found in the meta-analysis of psychology.

In Section 3.4, above, we discussed PET-PEESE as a method to reduce publication bias in psychology. Several researchers have questioned the validity of PET-PEESE and related meta-regression corrections for publication bias (based on the Egger regression) because SE can be correlated with effect size in the absence of publication bias.^35^ ^,^ ^42^ ^,^ ^43^ Thus, a surprising finding is that, even for correlations, where the correlation with SE in the absence of publication bias is mechanical, PET-PEESE works well to reduce PSB and type I errors when there is publication bias—see the columns associated with PP and PPz in Table 2. Average bias of PP is only about .01 when there is PSB, which is approximately four times smaller than conventional random-effects’ biases (using either correlations or Fisher’s *z* transformation). Despite the correlation between SE and *r* in the PET-PEESE meta-regressions, PET-PEESE has relatively excellent statistical properties. Especially relevant, note that PET’s type I errors are always within their nominal levels, whether or not there is publication bias. However, PET-PEESE is not perfect and can be improved through the Fisher’s *z* transformation because *z* is not correlated to its SE. PPz reports the statistical properties of first converting correlations to *z*, calculating PET-PEESE in terms of Fisher’s *z*, and lastly converting PET-PEESE in terms of *z* back to a correlation. On average, PPz has smaller bias, MSE, type I errors and better CIs than PP of correlations. However, there is a potential problem with using PPz in the place of PP. PPz is downwardly biased for small correlations (.11), and this is a rather crucial effect size range as Cohen’s guidelines suggest that anything less than .1 is “trivial” or “null.”^28^ In contrast, PP is never downwardly bias, and its upward biases are less than rounding error (< .01) for small “true” effect sizes. When analyzing effects that may be null or trivial, it would, therefore, be better to use the untransformed PET-PEESE but to rely on PPz in other cases. For the sake of simplicity, the untransformed PET and its PET-PEESE estimate are always good choices as meta-analysis methods for correlations.

Surprisingly, across the two most representative research conditions, Het and PSB (i.e., heterogeneity without PBS and heterogeneity with 50% PSB, respectively), HS, followed closely by UWLS_+3_, has the smallest average RMSE. Yet, these simple weighted averages do not correct for PSB, explicitly. Although PET-PEESE does adjust for PSB, the conditional switch between these two models adds a source of variability, hence increasing RMSE. The somewhat smaller RMSEs of HS and UWLS_+3_ are not justification to employ only these weighted averages when PSB is suspected. All weighted averages have unacceptable Type I errors (>99.6%) with 50% PSB. Because PSB can rarely be ruled out either *a priori* or though tests of PSB (as they all tend to have low power), PET-PEESE should be *routinely* reported along with either HS or UWLS_+3._

## 6 Conclusions

Conventional inverse-variance weighted meta-analyses of correlations are biased, even under ideal conditions. However, to isolate and to document these small-sample biases, past studies assumed that all studies in a meta-analysis had the same sample size, no heterogeneity, and no selection for statistical significance (i.e., no PSB).^2^ ^,^ ^17^ The purpose of this paper is to investigate the statistical properties of conventional meta-analysis methods under typical conditions widely seen in correlational research in psychology. We find that these small-sample biases remain although they are, for the most part, smaller than rounding error (<.01). Regardless, PSB is the larger threat. Under the typical conditions found among meta-analyses of psychology, the small-sample biases of conventional meta-analysis, alone, are of little consequence (<.01), unless they use the “correct” variance, Equation (3).

This study corroborates prior findings.^1^ ^,^ ^2^ The conventional formula for the variance of correlations, 
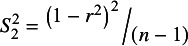
, often considered the “correct” variance of correlations,^3^ should never be used in meta-analysis as it is statistically dominated in all cases by a simpler formula, 
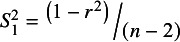
, that does not square the numerator.

When some results are selected for their statistical significance, PET-PEESE has no notable bias of scientific consequence (< .02), and tests of the correlation’s statistical significance (PET) maintain their nominal type I errors. This is an especially surprising finding as estimated correlations are mechanically correlated with their standard errors, though inversely so, in the absence of PSB. This correlation is seen by some to be a disqualifying condition for the application of PET-PEESE and the related Egger regression to meta-analysis.^42^ ^,^ ^43^ PET-PEESE is a notable improvement over RE even when averaged across research areas with or without PSB (see the last row of Table 2). With PSB, RE can have biases as large as the population mean correlation it is estimating, and RE is virtually certain (99.9%) to falsely identify statistically significant correlations that do not exist (see Table 2, Type I errors). It is publication selection bias that causes biases of notable scientific and practical consequences, not small-sample biases alone.

We also show that a new simple weighted average, UWLS_+3_, along with an older but infrequently employed weighted average, HS, statistically dominate RE whether or not correlations are first transformed to Fisher’s *z* (Table 2). This simple correction for small-sample bias, UWLS_+3_, adjusts the degrees of freedom and emerges as the preferred meta-analysis estimator in the absence of PSB along with HS and Fisher’s *z*. With PSB, PET-PEESE, using either correlations or Fisher’s *z*, has the best statistical properties under typical correlational research conditions. Unless publication selection bias can be ruled out *a priori*, we recommend researchers report PET-PEESE.

In sum, the central lessons of this study are:

- The small-sample biases of meta-analysis of correlations are rarely more than rounding errors (.01) unless the “correct” variance formula, Equation (3) is used.
- Several simple weighted averages (REz, HS, UWLS_+3_) provide adequate estimates of the mean effect in the absence of publication bias.
- With publication bias, PET-PEESE is surprisingly effective in spite of SE’s mechanical dependence upon the estimated correlation. Thus, PET-PEESE should be reported routinely in a large majority of meta-analyses.

Needless to say, there are limitations to our findings. Our findings apply fully only to the specifications that we simulate, which assume that meta-analyses have the typical conditions seen widely across correlational studies in psychology. However, not all meta-analyses involve “typical” correlational research. In particular, if all studies use small samples (*n* ≤ 100), small-sample biases will generally be larger, and PET-PEESE is no longer valid as there will be too little variation in SE and, as a result, PET-PEESE will produce unreliable estimates.^44^ When there is little variation in *SE*, this “independent” (or explanatory) variable in the PET-PEESE meta-regression will have little useful information with which to estimate its regression coefficient. In meta-analyses with little variation in SE, one should not employ PET-PEESE.^44^ ^,^ ^xiii^

Although not unique to the methods introduced here, coding errors and other influential data can distort any meta-analysis. To prevent any undue influence from one or a few overly influential effects, meta-analysts should always use influence statistics (also called leverage points or, incorrectly, “outliers”) to identify and correct, or remove such overly influential studies regardless of their cause. The criterion and method used to identify leverage points can be stated in a pre-analysis plan. Without the identification and removal of highly influential effect sizes, any meta-analysis result can be highly skewed towards simple coding/transcription/transformation error or, in rare cases, fraud.^xiv^

In summary, a simple adjustment to degrees of freedom, UWLS_+3_, along with those weighted averages that depend on sample size alone (Fisher’s *z* and the Hunter and Schmidt approach), will typically eliminate the small-sample biases of the meta-analysis of correlations to something less than rounding error (<.01). However, in practice, the larger problem is frequently publication selection biases. This study finds that PSB is effectively corrected by PET-PEESE under typical conditions seen widely across correlational studies in psychology.

## Supporting information

Stanley et al. supplementary material Stanley et al. supplementary material

## Data Availability

The data used in the illustration are available at: https://osf.io/8we4b/. Codes for the illustration and the simulations are given in the online supplement also at: https://osf.io/8we4b/.

## Notes

i. It should be noted that Fisher’s *z* transformation is not the same as the *z*-values, widely used throughout statistics and meta-analysis to represent the normal distribution.

ii. Figure 1 is not meant to imply that RE biases are larger than UWLS’s for the same variance formula and sample size. In fact, the contrary is true.^17^ Rather, Figure 1 is offered merely to show compactly and clearly that these biases are a near perfect function of the inverse degrees of freedom; hence, they must be small-sample biases.

iii. We thank an anonymous reviewer and the associate editor for suggesting the Hunter and Schmidt approach.

iv. Correlations are converted to *z* by: , and Fisher’s *z* is transformed back to correlations by: .

v. Only the precision-effect test (PET) provides a valid test (H_0_:  = 0, from Equation (12) for the presence of a nonzero mean effect, after correcting for potential PSB.

vi. Both WAAP and WILS calculate UWLS on a subset of the effect sizes. WAAP uses only those studies that have 80% or higher statistical power, while WILS first removes those estimates most responsible for excess statistical significance.

vii Because these intercepts are in the opposite direction of the meta-analysis estimates (i.e., negative), we interpret them as negligible. When the PET estimate is of the opposite sign as UWLS, PEESE, Equation (13), should not be calculated. In these cases, there is such a strong correlation with the standard error that any statistical evidence of a mean effect is erased once potential PSB in accommodated. PEESE should be employed only if there is some evidence of an effect in the predominant direction.

viii. Note that the magnitude of the estimated FAT coefficients, 1.94, is quite substantial. When, the FAT coefficient is two or larger, Doucouliagos and Stanley^36^ categorize this as “severe” publication selection because it implies that the average effect size is exaggerated by twice its SEs; just sufficient to make a null effect appear statistically significant. The SE of Fisher’s *z* does not depend on the magnitude of the correlation (or Fz); thus, this clear positive correlation with SE cannot be dismissed as a statistical artifact of its variance formula. Nor can the fact that the formula for  depends on *r* be used to dismiss its *positive* correlation, Equation (14), as this formula embeds a slight *negative* correlation.

ix. In psychology, the average number of estimated correlations per meta-analysis is 55.^4^ The biases of correlations are largely independent of the number of correlations (*k*) meta-analyzed. In contrast, the sample size (*n*) of the primary study used to calculate correlations is a very important determinant of this bias, as meta-analysis of correlations suffers from small-sample bias.^2^ In these simulations, we assume that the distribution of sample sizes reflects what is typically seen in psychology.

x. Random heterogeneity added to *ρ* produce asymmetric, nonnormal, sampling distributions that induce further estimation biases. Conversion to Cohen’s *d* avoids this added source of bias. Generating heterogeneity through random variations to *X_1_*’s regression coefficient,  , Equation (16), produces approximately the same overall results.

xi. The threshold for scientific or practical significance of a given bias would depend on what is a notable difference for the specific application in question. Here, we consider .05 to be a bias of potential scientific consequence as a null correlation < .1, according to Cohen’s guidelines, could easily double and become what most psychologists regard as “small” and non-null. ^28^ ^,^ ^34^

xii.
Table 2 reports that the type I error of the random effects estimates that are based on the *z*-transformation, REz, to be 99.85%. In all cases, REz has better statistical properties than the conventional RE of correlations. Compare RE_2_ in Table 1 to REz in Table 2.

xiii. However, it needs to be emphasized that meta-analyses in psychology typically have sufficient variation in sample size and SE to allow the PET-PEESE models to be reliably estimated. Across 596 psychology meta-analyses, the typical (median) ratio between the smallest sample size and the largest is a factor of 20.^41^ ^–^ ^45^ Thus, the typical distribution of sample sizes across psychology is wider than the distribution of sample sizes used in this paper’s simulations. In those rare cases where there is little variation among samples sizes (e.g., *n* ≤ 100, for all studies), we recommend Bayesian model averaging that lets the research record, itself, decide on the appropriate weights.^22^

xiv. An illustrative example comes from Kivikangas’ et al.^46^ meta-analysis of the correlation between moral foundations and political orientation. In this area of research, one study stands out, Graham et al.^47^ It uses an online survey, YourMorals.org, requiring the volition of over 200,000 individual subjects. Including this one study doubles the mean effect size, as Graham et al.^47^ reports both the largest correlations and the largest sample by more than an order of magnitude. This study’s large effect size is probably not an error and clearly is not fraud. As Kivikangas et al.^46^ argue, the large effect was likely the result of self-selection to participate by those with the more extreme political orientations. Regardless of cause, such overly influential studies need to be omitted or accommodated through moderator analysis, just as Kivikangas et al.^46^ did.

## Supplementary material

The supplementary material for this article can be found at https://doi.org/10.1017/rsm.2024.5.
