# Supplementary material for: Reducing the biases of the conventional meta-analysis of correlations
Source: Res Synth Methods. 2025 Apr 1;16(1):42–59. doi: 10.1017/rsm.2024.5 (PMC12631149; doi:10.1017/rsm.2024.5)
Supplement: Stanley et al. supplementary material [file S175928792400005Xsup001.docx]

**Online Supplement to:**

**“Reducing the biases of the conventional meta-analysis of correlations”**

T. D. Stanley^*^, Hristos Doucouliagos^*^, and Tomas Havranek^***^

1. STATA and R programs for UWLS

1. An R program for UWLS is:

#read in your data file that has a column for

# correlations, labeled "r", and standard errors

# as the sqrt of eq(2) labeled "ser".

pathName = "C:/Users/USER/Documents/MyData.csv"

dat = read.csv(pathName)

r = dat$r

ser = dat$ser

k = length(r) #number of studies

t = r/ser

Precision=1/ser

reg = lm(t ~ 0 + Precision)

UWLS = as.numeric(reg$coefficients)

2. A STATA program for UWLS

Merely, bring up STATA program and have a STATA datafile called “MyData.dta” that contains correlations, labeled "r", and standard errors labeled "ser".

*get data

cd "C:\Users \My\ Desktop\"

use " MyData.dta"

*transform variables

generate t=r/ser

generate Precision=1/ser

*calculate UWLS

regress t Precision, noconstant

* Department of Economics, Deakin University, 221 Burwood Highway, Burwood, 3125, Victoria, Australia. Email: [tom.stanley1@deakin.edu.au](mailto:tom.stanley1@deakin.edu.au)

** Department of Economics, Deakin University, 221 Burwood Highway, Burwood, 3125, Victoria, Australia. Email: douc@deakin.edu.au.

***Institute of Economic Studies, Faculty of Social Sciences, Charles University, Prague, Czech Republic.

B.  **STATA code for the illustration:**

*STATA code of this illustration starts with a STATA file containing two columns of data: r and n.

generate S1_sq = (1- r^2 )/( n-2 )

generate S2_sq = (1- r^2 )^2/( n-1 )

generate S2 = sqrt( S2_sq )

generate S1 = sqrt( S1_sq )

generate tr = r/S1

generate r3 = tr/( tr^2+ n+1 )^.5

generate Fz = .5* ln((1+ r )/(1-r ))

generate t3 = r3/S1

generate precision = 1/S1

generate SEz = 1/ sqrt(n-3)

generate preZ = sqrt(n-3)

generate tz = Fz/SEz

generate precision_sq = precision^2

generate preZ_sq = preZ^2

*Random effects

meta set r S2, random

meta summarize

*REz

meta set Fz SEz, random

meta summarize

*UWLS_+3_

regress t3 precision, noconstant

* PET regression, eq. (7)

regress r S1 [aweight = precision_sq]

* PET regression, eq. (7), for REz

regress Fz SEz [aweight = preZ_sq]

*convert Fz results back to correlation by $\frac{(e^{\left( 2z \right)}-1)}{(e^{\left( 2z \right)}+1)}$

Data are available at**:** <https://osf.io/8we4b/>

Calculations for the Hunter and Schmidt (1990) approach were done on a spreadsheet also available at: <https://osf.io/8we4b/>

**Results**

**RE**

meta set r S2, random

meta summarize

meta summarize

Effect-size label: Effect Size

Effect size: r

Std. Err.: S2

Meta-analysis summary Number of studies = 73

Random-effects model Heterogeneity:

Method: REML tau2 = 0.0164

I2 (%) = 85.45

H2 = 6.87

theta | 0.128 0.092 0.164

REML estimate of tau

**REz**

meta set Fz SEz, random

Meta-analysis setting information

Study information

No. of studies: 73

Study label: Generic

Study size: N/A

Effect size

Type: Generic

Label: Effect Size

Variable: Fz

Precision

Std. Err.: Sez

. meta summarize

Effect-size label: Effect Size

Effect size: Fz

Std. Err.: SEz

Meta-analysis summary Number of studies = 73

Random-effects model Heterogeneity:

Method: DerSimonian-Laird tau2 = 0.0132

I2 (%) = 81.85

H2 = 5.51

theta | 0.128 0.092 0.164

Converted back to correlations using (exp(2 * "Fishers z")-1)/(exp(2 * "Fishers z")+1))

gives: .127 (.092, .163)

**UWLS+3**

regress t3 precision, noconstant

Source | SS df MS Number of obs = 73

-------------+---------------------------------- F(1, 72) = 12.41

Model | 69.1019498 1 69.1019498 Prob > F = 0.0007

Residual | 400.967176 72 5.56898855 R-squared = 0.1470

-------------+---------------------------------- Adj R-squared = 0.1352

Total | 470.069126 73 6.43930309 Root MSE = 2.3599

------------------------------------------------------------------------------

t3 | Coef. Std. Err. t P>|t| [95% Conf. Interval]

-------------+----------------------------------------------------------------

precision | .049867 .0141565 3.52 0.001 .0216465 .0780875

FAT-PET Eq.(12)

regress r S1 [aweight = precision_sq]

(sum of wgt is 27,788.4329719543)

Source | SS df MS Number of obs = 73

-------------+---------------------------------- F(1, 71) = 26.91

Model | .294550231 1 .294550231 Prob > F = 0.0000

Residual | .777200268 71 .010946483 R-squared = 0.2748

-------------+---------------------------------- Adj R-squared = 0.2646

Total | 1.0717505 72 .014885424 Root MSE = .10463

------------------------------------------------------------------------------

r | Coef. Std. Err. t P>|t| [95% Conf. Interval]

-------------+----------------------------------------------------------------

S1 | 1.943058 .3745789 5.19 0.000 1.196168 2.689947

_cons | -.026349 .0191987 -1.37 0.174 -.0646302 .0119322

FAT-PET-Z

regress Fz SEz [aweight = preZ_sq]

(sum of wgt is 27,237.000146389)

Source | SS df MS Number of obs = 73

-------------+---------------------------------- F(1, 71) = 29.07

Model | .308913563 1 .308913563 Prob > F = 0.0000

Residual | .754432902 71 .010625816 R-squared = 0.2905

-------------+---------------------------------- Adj R-squared = 0.2805

Total | 1.06334646 72 .014768701 Root MSE = .10308

------------------------------------------------------------------------------

Fz | Coef. Std. Err. t P>|t| [95% Conf. Interval]

-------------+----------------------------------------------------------------

SEz | 1.944126 .3605679 5.39 0.000 1.225174 2.663078

_cons | -.0290044 .0186668 -1.55 0.125 -.0662248 .0082161

C. GAUSS program for Tables 1 and 2

**Table 1:**

/************************************************************************/

/* PROGRAM: Correlations with different n, het, & pub'bias */

/* AUTHOR: Tom Stanley 01:39 PM 07-Jun-2023 */

/************************************************************************/

new;

screen off;

rep=10000;/*the number of replications*/

sum1=0;sum2=0;sum3=0;sum10=0;sum11=0;

MSE1=0;MSE2=0;MSE3=0;MSE4=0;

sum4=0;sum5=0;sum6=0;sum7=0;sum9=0;sum8=0;

cov1=0;cov4=0;cov2=0;cov3=0;

ID={30,40,50,75,100,100,125,160,200,400};/* sample size of the original studies*/

het={.45,.45,.3,.3,.3,.3,.3,.3,.075,.075};

critt={2.048,2.024,2.011,1.993,1.984,1.984,1.98,1.978,1.975,1.97};

k=1;

do while (k<=rep);/* the number of replications*/

Seinv1={};Seinv2={};Seinv3={};Seinv4={};

t1={}; t2={};r={};t3={};t4={};

W1i={};Zf={};

Ssq1={};Ssq3={};

W2i={};W3i={};W4i={};

Ssq2={}; Ssq4={};

r2=5;r1=0;/* the number of research studies is the sum times 10*/

j=1;

do while (j<=r2);

i=1;

do while (i<=10);

X2=rndn(ID[i],1)/2;

/* The below generates random subject data for each of 50 studies*/

/*Bivariate Correlation using regressions' t-values */

X1=ones(ID[i],1);

Y=X1+X2+rndn(ID[i],1);

X=X2;

_olsres=1;

{ nam,m,b,stb,vc,std,sig,cx,rsq,resid,dbw } = ols(0,Y,X);

ti=b[2]/std[2];

corrpart=ti^2+ID[i]-2;corr=ti/sqrt(corrpart);

b1=0;/*adds random Het in terms of Cohen's d*/

d1=b1+2*corr/(1-corr^2)^.5;corr=d1/(d1^2+4)^.5;

/* SEtest is the SE for testing whether corr=0*/

SEtest=sqrt((1-corr^2)/(ID[i]-2));

/* SEma is the conventional meta-analysis SE for corr's*/

SEma=(1-corr^2)/sqrt((ID[i]-1));

t1i=corr/SEma;Seinv1i=1/SEma;

t2i=corr/SEtest;Seinv2i=1/SEtest;

Seinv1=Seinv1|Seinv1i;t1=t1|t1i;

Seinv2=Seinv2|Seinv2i;t2=t2|t2i;

r=r|corr;

W1ii=Seinv1i^2;

W2ii=Seinv2i^2;

W1i=W1i|W1ii;W2i=W2i|W2ii;

Ssq1=Ssq1|SEma^2;Ssq2=Ssq2|SEtest^2;

i=i+1;

endo;

j=j+1;

endo;

/* The above program does not select results at all */

/* The below program selects entirely for statistical significance */

j=1;

do while (j<=r1);

i=1;

do while (i<=10);

ti=0;

do until ti>=critt[i];

X2=rndn(ID[i],1)/2;

/*Bivariate Correlation using regressions' t-values */

X1=ones(ID[i],1);

Y=X1+X2+rndn(ID[i],1);

X=X2;

_olsres=1;

{ nam,m,b,stb,vc,std,sig,cx,rsq,resid,dbw } = ols(0,Y,X);

ti=b[2]/std[2];

corrpart=ti^2+ID[i]-2;corr=ti/sqrt(corrpart);

b1=0;/*adds random Het in terms of Cohen's d*/

d1=b1+2*corr/(1-corr^2)^.5;corr=d1/(d1^2+4)^.5;

/* SEtest is the SE for testing whether corr=0*/

SEtest=sqrt((1-corr^2)/(ID[i]-2));

ti=corr/SEtest;

endo;

/* SEma is the conventional meta-analysis SE for corr's*/

SEma=(1-corr^2)/sqrt((ID[i]-1));

t1i=corr/SEma;Seinv1i=1/SEma;

t2i=corr/SEtest;Seinv2i=1/SEtest;

Seinv1=Seinv1|Seinv1i;t1=t1|t1i;

Seinv2=Seinv2|Seinv2i;t2=t2|t2i;

r=r|corr;

W1ii=Seinv1i^2;

W2ii=Seinv2i^2;

W1i=W1i|W1ii;W2i=W2i|W2ii;

Ssq1=Ssq1|SEma^2;Ssq2=Ssq2|SEtest^2;

i=i+1;

endo;

j=j+1;

endo;

rr=10*(r2+r1);

z1=ones(rr,1);

ave=r'*z1/rr; sum3=sum3+ave;

sumW1=W1i'*z1;sumW2=W2i'*z1;

/* The below program calculates the unrestricted WLS2 & UWLS1 */

bb1=inv(Seinv2'*Seinv2)*Seinv2't2;sum1=sum1+bb1;

s2wls=(t2-Seinv2*bb1)'*(t2-Seinv2*bb1)/(rr-1);

WLSVAR1=s2wls*inv(Seinv1'*Seinv1);MSE3=MSE3+(bb1[1]-sqrt(1/5))^2;

UL1=bb1[1]+2.01*sqrt(WLSVAR1);

LL1=bb1[1]-2.01*sqrt(WLSVAR1);

if LL1<=sqrt(1/5) and UL1>=sqrt(1/5);

cov3=cov3+1;

endif;

bb2=inv(Seinv1'*Seinv1)*Seinv1't1;sum2=sum2+bb2;

s2wls2=(t1-Seinv1*bb2)'*(t1-Seinv1*bb2)/(rr-1);

WLSVAR2=s2wls2*inv(Seinv1'*Seinv1);MSE4=MSE4+(bb2[1]-sqrt(1/5))^2;

UL2=bb2[1]+2.01*sqrt(WLSVAR2);

LL2=bb2[1]-2.01*sqrt(WLSVAR2);

if LL2<=sqrt(1/5) and UL2>=sqrt(1/5);

cov4=cov4+1;

endif;

I2=(s2wls2-1)/s2wls2;

if I2<0;I2=0;endif;sum8=sum8+I2;

/* The below program calculates RE */

Wave1=sumW1/rr;/*see Eq (5.3) in Sutton et al (2000) */

S2w1=(t1'*z1-rr*Wave1^2)/(rr-1);/*Eq (5.4)*/

U=(rr-1)*(Wave1-S2w1/(rr*Wave1));/*Eq (5.5)*/

Q=(W1i'r^2)-(r'*W1i)^2/sumW1;/*Eq (3.2)*/

BSvar=(Q-rr+1)/U;/*Eq (5.6)*/

if Q<=rr-1;

BSvar=0;

endif;

iBS=1; weight={};

do while (iBS<=rr);

weighti=1/(BSvar+Ssq1[iBS]);/*Eq(5.7)*/

weight=weight|weighti;

iBS=iBS+1;

endo;

sumWeight1=weight'*z1;

REWA1=(r'*weight)/sumWeight1; /*Eq(5.8)*/

VARRE=1/sumWeight1;

ulr=REWA1+1.96*sqrt(VARRE);

llr=REWA1-1.96*sqrt(VARRE);

if llr<=sqrt(1/5) and ulr>=sqrt(1/5);

cov1=cov1+1;

endif;

sum5=sum5+REWA1;MSE1=MSE1+(REWA1-sqrt(1/5))^2;

Wave2=sumW2/rr;/*see Eq (5.3) in Sutton et al (2000) */

S2w2=(t2'*z1-rr*Wave2^2)/(rr-1);/*Eq (5.4)*/

U=(rr-1)*(Wave2-S2w2/(rr*Wave2));/*Eq (5.5)*/

Q=(W2i'r^2)-(r'*W2i)^2/sumW2;/*Eq (3.2)*/

BSvar=(Q-rr+1)/U;/*Eq (5.6)*/

if Q<=rr-1;

BSvar=0;

endif;

iBS=1; weight={};

do while (iBS<=rr);

weighti=1/(BSvar+Ssq2[iBS]);/*Eq(5.7)*/

weight=weight|weighti;

iBS=iBS+1;

endo;

sumWeight2=weight'*z1;

REWA2=(r'*weight)/sumWeight2; /*Eq(5.8)*/

VARRE2=1/sumWeight2;

ulr=REWA2+1.96*sqrt(VARRE2);

llr=REWA2-1.96*sqrt(VARRE2);

if llr<=sqrt(1/5) and ulr>=sqrt(1/5);

cov2=cov2+1;

endif;

sum7=sum7+REWA2;MSE2=MSE2+(REWA2-sqrt(1/5))^2;

k=k+1;

endo;

UWLSBias1=(sum1/rep)-sqrt(1/5);UWLScov1=cov3/rep;

UWLSBias2=(sum2/rep)-sqrt(1/5);UWLScov2=cov4/rep;

REBias1=(sum5/rep)-sqrt(1/5);REcov1=cov1/rep;Isq=sum8/rep;

REBias2=(sum7/rep)-sqrt(1/5);REcov2=cov2/rep;

WLSRMSE1=(MSE3/rep)^.5;WLSRMSE2=(MSE4/rep)^.5;

RERMSE1=(MSE1/rep)^.5;RERMSE2=(MSE2/rep)^.5;

AveBias=(sum3/rep)-sqrt(1/5);

screen on;

"RE & UWLS; NoHet, CorrHet & PB50;k=50;n={30,40,50,75,100,100,125,160,200,400};True r=sqrt{1/2;1/4;1/9}";

"I-sq, Average bias, REBias1(SEma), REBias2SE(test), UWLS1Bias,UWLS2bias,Coverages, MSEs";

Isq;;AveBias;;REBias1;;REBias2;;UWLSBias2;;UWLSBias1;;REcov1;;REcov2;;UWLScov2;;UWLScov1;;RERMSE1;;RERMSE2;;WLSRMSE2;;WLSRMSE1;

screen off;

sum1=0;sum2=0;sum3=0;sum10=0;sum11=0;

MSE1=0;MSE2=0;MSE3=0;MSE4=0;

sum4=0;sum5=0;sum6=0;sum7=0;sum9=0;sum8=0;

cov1=0;cov4=0;cov2=0;cov3=0;

k=1;

do while (k<=rep);/* the number of replications*/

Seinv1={};Seinv2={};Seinv3={};Seinv4={};

t1={}; t2={};r={};t3={};t4={};

W1i={};Zf={};

Ssq1={};Ssq3={};

W2i={};W3i={};W4i={};

Ssq2={}; Ssq4={};

/* space holder 1*/

/* space holder 2*/

/* space holder 3*/

r2=5;r1=0;/* the number of research studies is the sum times 10*/

j=1;

do while (j<=r2);

i=1;

do while (i<=10);

X2=rndn(ID[i],1)/4;

/* space holder 4*/

/* space holder 5*/

/* space holder 6*/

/*Bivariate Correlation using regressions' t-values */

X1=ones(ID[i],1);

Y=X1+X2+rndn(ID[i],1);

X=X2;

_olsres=1;

{ nam,m,b,stb,vc,std,sig,cx,rsq,resid,dbw } = ols(0,Y,X);

ti=b[2]/std[2];

corrpart=ti^2+ID[i]-2;corr=ti/sqrt(corrpart);

b1=0;/*adds random Het in terms of Cohen's d*/

d1=b1+2*corr/(1-corr^2)^.5;corr=d1/(d1^2+4)^.5;

/* SEtest is the SE for testing whether corr=0*/

SEtest=sqrt((1-corr^2)/(ID[i]-2));

/* SEma is the conventional meta-analysis SE for corr's*/

SEma=(1-corr^2)/sqrt((ID[i]-1));

t1i=corr/SEma;Seinv1i=1/SEma;

t2i=corr/SEtest;Seinv2i=1/SEtest;

Seinv1=Seinv1|Seinv1i;t1=t1|t1i;

Seinv2=Seinv2|Seinv2i;t2=t2|t2i;

r=r|corr;

W1ii=Seinv1i^2;

W2ii=Seinv2i^2;

W1i=W1i|W1ii;W2i=W2i|W2ii;

Ssq1=Ssq1|SEma^2;Ssq2=Ssq2|SEtest^2;

i=i+1;

endo;

j=j+1;

endo;

/* The above program does not select results at all */

/* The below program selects entirely for statistical significance */

j=1;

do while (j<=r1);

i=1;

do while (i<=10);

ti=0;

do until ti>=critt[i];

X2=rndn(ID[i],1)/4;

/*Bivariate Correlation using regressions' t-values */

/* space holder 4*/

/* space holder 5*/

/* space holder 6*/

X1=ones(ID[i],1);

Y=X1+X2+rndn(ID[i],1);

X=X2;

_olsres=1;

{ nam,m,b,stb,vc,std,sig,cx,rsq,resid,dbw } = ols(0,Y,X);

ti=b[2]/std[2];

corrpart=ti^2+ID[i]-2;corr=ti/sqrt(corrpart);

b1=0;/*adds random Het in terms of Cohen's d*/

d1=b1+2*corr/(1-corr^2)^.5;corr=d1/(d1^2+4)^.5;

/* SEtest is the SE for testing whether corr=0*/

SEtest=sqrt((1-corr^2)/(ID[i]-2));

ti=corr/SEtest;

endo;

/* SEma is the conventional meta-analysis SE for corr's*/

SEma=(1-corr^2)/sqrt((ID[i]-1));

t1i=corr/SEma;Seinv1i=1/SEma;

t2i=corr/SEtest;Seinv2i=1/SEtest;

Seinv1=Seinv1|Seinv1i;t1=t1|t1i;

Seinv2=Seinv2|Seinv2i;t2=t2|t2i;

r=r|corr;

W1ii=Seinv1i^2;

W2ii=Seinv2i^2;

W1i=W1i|W1ii;W2i=W2i|W2ii;

Ssq1=Ssq1|SEma^2;Ssq2=Ssq2|SEtest^2;

i=i+1;

endo;

j=j+1;

endo;

rr=10*(r2+r1);

z1=ones(rr,1);

ave=r'*z1/rr; sum3=sum3+ave;

sumW1=W1i'*z1;sumW2=W2i'*z1;

/* The below program calculates the unrestricted WLS2 & UWLS1 */

bb1=inv(Seinv2'*Seinv2)*Seinv2't2;sum1=sum1+bb1;

s2wls=(t2-Seinv2*bb1)'*(t2-Seinv2*bb1)/(rr-1);

WLSVAR1=s2wls*inv(Seinv1'*Seinv1);MSE3=MSE3+(bb1[1]-sqrt(1/17))^2;

UL1=bb1[1]+2.01*sqrt(WLSVAR1);

LL1=bb1[1]-2.01*sqrt(WLSVAR1);

if LL1<=sqrt(1/17) and UL1>=sqrt(1/17);

cov3=cov3+1;

endif;

bb2=inv(Seinv1'*Seinv1)*Seinv1't1;sum2=sum2+bb2;

s2wls2=(t1-Seinv1*bb2)'*(t1-Seinv1*bb2)/(rr-1);

WLSVAR2=s2wls2*inv(Seinv1'*Seinv1);MSE4=MSE4+(bb2[1]-sqrt(1/17))^2;

UL2=bb2[1]+2.01*sqrt(WLSVAR2);

LL2=bb2[1]-2.01*sqrt(WLSVAR2);

if LL2<=sqrt(1/17) and UL2>=sqrt(1/17);

cov4=cov4+1;

endif;

I2=(s2wls2-1)/s2wls2;

if I2<0;I2=0;endif;sum8=sum8+I2;

/* The below program calculates RE */

Wave1=sumW1/rr;/*see Eq (5.3) in Sutton et al (2000) */

S2w1=(t1'*z1-rr*Wave1^2)/(rr-1);/*Eq (5.4)*/

U=(rr-1)*(Wave1-S2w1/(rr*Wave1));/*Eq (5.5)*/

Q=(W1i'r^2)-(r'*W1i)^2/sumW1;/*Eq (3.2)*/

BSvar=(Q-rr+1)/U;/*Eq (5.6)*/

if Q<=rr-1;

BSvar=0;

endif;

iBS=1; weight={};

do while (iBS<=rr);

weighti=1/(BSvar+Ssq1[iBS]);/*Eq(5.7)*/

weight=weight|weighti;

iBS=iBS+1;

endo;

sumWeight1=weight'*z1;

REWA1=(r'*weight)/sumWeight1; /*Eq(5.8)*/

VARRE=1/sumWeight1;

ulr=REWA1+1.96*sqrt(VARRE);

llr=REWA1-1.96*sqrt(VARRE);

if llr<=sqrt(1/17) and ulr>=sqrt(1/17);

cov1=cov1+1;

endif;

sum5=sum5+REWA1;MSE1=MSE1+(REWA1-sqrt(1/17))^2;

Wave2=sumW2/rr;/*see Eq (5.3) in Sutton et al (2000) */

S2w2=(t2'*z1-rr*Wave2^2)/(rr-1);/*Eq (5.4)*/

U=(rr-1)*(Wave2-S2w2/(rr*Wave2));/*Eq (5.5)*/

Q=(W2i'r^2)-(r'*W2i)^2/sumW2;/*Eq (3.2)*/

BSvar=(Q-rr+1)/U;/*Eq (5.6)*/

if Q<=rr-1;

BSvar=0;

endif;

iBS=1; weight={};

do while (iBS<=rr);

weighti=1/(BSvar+Ssq2[iBS]);/*Eq(5.7)*/

weight=weight|weighti;

iBS=iBS+1;

endo;

sumWeight2=weight'*z1;

REWA2=(r'*weight)/sumWeight2; /*Eq(5.8)*/

VARRE2=1/sumWeight2;

ulr=REWA2+1.96*sqrt(VARRE2);

llr=REWA2-1.96*sqrt(VARRE2);

if llr<=sqrt(1/17) and ulr>=sqrt(1/17);

cov2=cov2+1;

endif;

sum7=sum7+REWA2;MSE2=MSE2+(REWA2-sqrt(1/17))^2;

k=k+1;

endo;

UWLSBias1=(sum1/rep)-sqrt(1/17);UWLScov1=cov3/rep;

UWLSBias2=(sum2/rep)-sqrt(1/17);UWLScov2=cov4/rep;

REBias1=(sum5/rep)-sqrt(1/17);REcov1=cov1/rep;Isq=sum8/rep;

REBias2=(sum7/rep)-sqrt(1/17);REcov2=cov2/rep;

WLSRMSE1=(MSE3/rep)^.5;WLSRMSE2=(MSE4/rep)^.5;

RERMSE1=(MSE1/rep)^.5;RERMSE2=(MSE2/rep)^.5;

AveBias=(sum3/rep)-sqrt(1/17);

screen on;

Isq;;AveBias;;REBias1;;REBias2;;UWLSBias2;;UWLSBias1;;REcov1;;REcov2;;UWLScov2;;UWLScov1;;RERMSE1;;RERMSE2;;WLSRMSE2;;WLSRMSE1;

screen off;

sum1=0;sum2=0;sum3=0;sum10=0;sum11=0;

MSE1=0;MSE2=0;MSE3=0;MSE4=0;

sum4=0;sum5=0;sum6=0;sum7=0;sum9=0;sum8=0;

cov1=0;cov4=0;cov2=0;cov3=0;

k=1;

do while (k<=rep);/* the number of replications*/

Seinv1={};Seinv2={};Seinv3={};Seinv4={};

t1={}; t2={};r={};t3={};t4={};

W1i={};Zf={};

Ssq1={};Ssq3={};

W2i={};W3i={};W4i={};

Ssq2={}; Ssq4={};

/* space holder 1*/

/* space holder 2*/

/* space holder 3*/

r2=5;r1=0;/* the number of research studies is the sum times 10*/

j=1;

do while (j<=r2);

i=1;

do while (i<=10);

X2=rndn(ID[i],1)/9;

/* space holder 4*/

/* space holder 5*/

/* space holder 6*/

/*Bivariate Correlation using regressions' t-values */

X1=ones(ID[i],1);

Y=X1+X2+rndn(ID[i],1);

X=X2;

_olsres=1;

{ nam,m,b,stb,vc,std,sig,cx,rsq,resid,dbw } = ols(0,Y,X);

ti=b[2]/std[2];

corrpart=ti^2+ID[i]-2;corr=ti/sqrt(corrpart);

b1=0;/*adds random Het in terms of Cohen's d*/

d1=b1+2*corr/(1-corr^2)^.5;corr=d1/(d1^2+4)^.5;

/* SEtest is the SE for testing whether corr=0*/

SEtest=sqrt((1-corr^2)/(ID[i]-2));

/* SEma is the conventional meta-analysis SE for corr's*/

SEma=(1-corr^2)/sqrt((ID[i]-1));

t1i=corr/SEma;Seinv1i=1/SEma;

t2i=corr/SEtest;Seinv2i=1/SEtest;

Seinv1=Seinv1|Seinv1i;t1=t1|t1i;

Seinv2=Seinv2|Seinv2i;t2=t2|t2i;

r=r|corr;

W1ii=Seinv1i^2;

W2ii=Seinv2i^2;

W1i=W1i|W1ii;W2i=W2i|W2ii;

Ssq1=Ssq1|SEma^2;Ssq2=Ssq2|SEtest^2;

i=i+1;

endo;

j=j+1;

endo;

/* The above program does not select results at all */

/* The below program selects entirely for statistical significance */

j=1;

do while (j<=r1);

i=1;

do while (i<=10);

ti=0;

do until ti>=critt[i];

X2=rndn(ID[i],1)/9;

/*Bivariate Correlation using regressions' t-values */

/* space holder 4*/

/* space holder 5*/

/* space holder 6*/

X1=ones(ID[i],1);

Y=X1+X2+rndn(ID[i],1);

X=X2;

_olsres=1;

{ nam,m,b,stb,vc,std,sig,cx,rsq,resid,dbw } = ols(0,Y,X);

ti=b[2]/std[2];

corrpart=ti^2+ID[i]-2;corr=ti/sqrt(corrpart);

b1=0;/*adds random Het in terms of Cohen's d*/

d1=b1+2*corr/(1-corr^2)^.5;corr=d1/(d1^2+4)^.5;

/* SEtest is the SE for testing whether corr=0*/

SEtest=sqrt((1-corr^2)/(ID[i]-2));

ti=corr/SEtest;

endo;

/* SEma is the conventional meta-analysis SE for corr's*/

SEma=(1-corr^2)/sqrt((ID[i]-1));

t1i=corr/SEma;Seinv1i=1/SEma;

t2i=corr/SEtest;Seinv2i=1/SEtest;

Seinv1=Seinv1|Seinv1i;t1=t1|t1i;

Seinv2=Seinv2|Seinv2i;t2=t2|t2i;

r=r|corr;

W1ii=Seinv1i^2;

W2ii=Seinv2i^2;

W1i=W1i|W1ii;W2i=W2i|W2ii;

Ssq1=Ssq1|SEma^2;Ssq2=Ssq2|SEtest^2;

i=i+1;

endo;

j=j+1;

endo;

rr=10*(r2+r1);

z1=ones(rr,1);

ave=r'*z1/rr; sum3=sum3+ave;

sumW1=W1i'*z1;sumW2=W2i'*z1;

/* The below program calculates the unrestricted WLS2 & UWLS1 */

bb1=inv(Seinv2'*Seinv2)*Seinv2't2;sum1=sum1+bb1;

s2wls=(t2-Seinv2*bb1)'*(t2-Seinv2*bb1)/(rr-1);

WLSVAR1=s2wls*inv(Seinv1'*Seinv1);MSE3=MSE3+(bb1[1]-sqrt(1/82))^2;

UL1=bb1[1]+2.01*sqrt(WLSVAR1);

LL1=bb1[1]-2.01*sqrt(WLSVAR1);

if LL1<=sqrt(1/82) and UL1>=sqrt(1/82);

cov3=cov3+1;

endif;

bb2=inv(Seinv1'*Seinv1)*Seinv1't1;sum2=sum2+bb2;

s2wls2=(t1-Seinv1*bb2)'*(t1-Seinv1*bb2)/(rr-1);

WLSVAR2=s2wls2*inv(Seinv1'*Seinv1);MSE4=MSE4+(bb2[1]-sqrt(1/82))^2;

UL2=bb2[1]+2.01*sqrt(WLSVAR2);

LL2=bb2[1]-2.01*sqrt(WLSVAR2);

if LL2<=sqrt(1/82) and UL2>=sqrt(1/82);

cov4=cov4+1;

endif;

I2=(s2wls2-1)/s2wls2;

if I2<0;I2=0;endif;sum8=sum8+I2;

/* The below program calculates RE */

Wave1=sumW1/rr;/*see Eq (5.3) in Sutton et al (2000) */

S2w1=(t1'*z1-rr*Wave1^2)/(rr-1);/*Eq (5.4)*/

U=(rr-1)*(Wave1-S2w1/(rr*Wave1));/*Eq (5.5)*/

Q=(W1i'r^2)-(r'*W1i)^2/sumW1;/*Eq (3.2)*/

BSvar=(Q-rr+1)/U;/*Eq (5.6)*/

if Q<=rr-1;

BSvar=0;

endif;

iBS=1; weight={};

do while (iBS<=rr);

weighti=1/(BSvar+Ssq1[iBS]);/*Eq(5.7)*/

weight=weight|weighti;

iBS=iBS+1;

endo;

sumWeight1=weight'*z1;

REWA1=(r'*weight)/sumWeight1; /*Eq(5.8)*/

VARRE=1/sumWeight1;

ulr=REWA1+1.96*sqrt(VARRE);

llr=REWA1-1.96*sqrt(VARRE);

if llr<=sqrt(1/82) and ulr>=sqrt(1/82);

cov1=cov1+1;

endif;

sum5=sum5+REWA1;MSE1=MSE1+(REWA1-sqrt(1/82))^2;

Wave2=sumW2/rr;/*see Eq (5.3) in Sutton et al (2000) */

S2w2=(t2'*z1-rr*Wave2^2)/(rr-1);/*Eq (5.4)*/

U=(rr-1)*(Wave2-S2w2/(rr*Wave2));/*Eq (5.5)*/

Q=(W2i'r^2)-(r'*W2i)^2/sumW2;/*Eq (3.2)*/

BSvar=(Q-rr+1)/U;/*Eq (5.6)*/

if Q<=rr-1;

BSvar=0;

endif;

iBS=1; weight={};

do while (iBS<=rr);

weighti=1/(BSvar+Ssq2[iBS]);/*Eq(5.7)*/

weight=weight|weighti;

iBS=iBS+1;

endo;

sumWeight2=weight'*z1;

REWA2=(r'*weight)/sumWeight2; /*Eq(5.8)*/

VARRE2=1/sumWeight2;

ulr=REWA2+1.96*sqrt(VARRE2);

llr=REWA2-1.96*sqrt(VARRE2);

if llr<=sqrt(1/82) and ulr>=sqrt(1/82);

cov2=cov2+1;

endif;

sum7=sum7+REWA2;MSE2=MSE2+(REWA2-sqrt(1/82))^2;

k=k+1;

endo;

UWLSBias1=(sum1/rep)-sqrt(1/82);UWLScov1=cov3/rep;

UWLSBias2=(sum2/rep)-sqrt(1/82);UWLScov2=cov4/rep;

REBias1=(sum5/rep)-sqrt(1/82);REcov1=cov1/rep;Isq=sum8/rep;

REBias2=(sum7/rep)-sqrt(1/82);REcov2=cov2/rep;

WLSRMSE1=(MSE3/rep)^.5;WLSRMSE2=(MSE4/rep)^.5;

RERMSE1=(MSE1/rep)^.5;RERMSE2=(MSE2/rep)^.5;

AveBias=(sum3/rep)-sqrt(1/82);

screen on;

Isq;;AveBias;;REBias1;;REBias2;;UWLSBias2;;UWLSBias1;;REcov1;;REcov2;;UWLScov2;;UWLScov1;;RERMSE1;;RERMSE2;;WLSRMSE2;;WLSRMSE1;

screen off;

sum1=0;sum2=0;sum3=0;sum10=0;sum11=0;

MSE1=0;MSE2=0;MSE3=0;MSE4=0;

sum4=0;sum5=0;sum6=0;sum7=0;sum9=0;sum8=0;

cov1=0;cov4=0;cov2=0;cov3=0;

k=1;

do while (k<=rep);/* the number of replications*/

Seinv1={};Seinv2={};Seinv3={};Seinv4={};

t1={}; t2={};r={};t3={};t4={};

W1i={};Zf={};

Ssq1={};Ssq3={};

W2i={};W3i={};W4i={};

Ssq2={}; Ssq4={};

/* space holder 1*/

/* space holder 2*/

/* space holder 3*/

r2=5;r1=0;/* the number of research studies is the sum times 10*/

j=1;

do while (j<=r2);

i=1;

do while (i<=10);

X2=rndn(ID[i],1)/2;

/* space holder 4*/

/* space holder 5*/

/* space holder 6*/

/*Bivariate Correlation using regressions' t-values */

X1=ones(ID[i],1);

Y=X1+X2+rndn(ID[i],1);

X=X2;

_olsres=1;

{ nam,m,b,stb,vc,std,sig,cx,rsq,resid,dbw } = ols(0,Y,X);

ti=b[2]/std[2];

corrpart=ti^2+ID[i]-2;corr=ti/sqrt(corrpart);

b1=het[i]*rndn(1,1);/*adds random Het in terms of Cohen's d*/

d1=b1+2*corr/(1-corr^2)^.5;corr=d1/(d1^2+4)^.5;

/* SEtest is the SE for testing whether corr=0*/

SEtest=sqrt((1-corr^2)/(ID[i]-2));

/* SEma is the conventional meta-analysis SE for corr's*/

SEma=(1-corr^2)/sqrt((ID[i]-1));

t1i=corr/SEma;Seinv1i=1/SEma;

t2i=corr/SEtest;Seinv2i=1/SEtest;

Seinv1=Seinv1|Seinv1i;t1=t1|t1i;

Seinv2=Seinv2|Seinv2i;t2=t2|t2i;

r=r|corr;

W1ii=Seinv1i^2;

W2ii=Seinv2i^2;

W1i=W1i|W1ii;W2i=W2i|W2ii;

Ssq1=Ssq1|SEma^2;Ssq2=Ssq2|SEtest^2;

i=i+1;

endo;

j=j+1;

endo;

/* The above program does not select results at all */

/* The below program selects entirely for statistical significance */

j=1;

do while (j<=r1);

i=1;

do while (i<=10);

ti=0;

do until ti>=critt[i];

X2=rndn(ID[i],1)/2;

/*Bivariate Correlation using regressions' t-values */

/* space holder 4*/

/* space holder 5*/

/* space holder 6*/

X1=ones(ID[i],1);

Y=X1+X2+rndn(ID[i],1);

X=X2;

_olsres=1;

{ nam,m,b,stb,vc,std,sig,cx,rsq,resid,dbw } = ols(0,Y,X);

ti=b[2]/std[2];

corrpart=ti^2+ID[i]-2;corr=ti/sqrt(corrpart);

b1=het[i]*rndn(1,1);/*adds random Het in terms of Cohen's d*/

d1=b1+2*corr/(1-corr^2)^.5;corr=d1/(d1^2+4)^.5;

/* SEtest is the SE for testing whether corr=0*/

SEtest=sqrt((1-corr^2)/(ID[i]-2));

ti=corr/SEtest;

endo;

/* SEma is the conventional meta-analysis SE for corr's*/

SEma=(1-corr^2)/sqrt((ID[i]-1));

t1i=corr/SEma;Seinv1i=1/SEma;

t2i=corr/SEtest;Seinv2i=1/SEtest;

Seinv1=Seinv1|Seinv1i;t1=t1|t1i;

Seinv2=Seinv2|Seinv2i;t2=t2|t2i;

r=r|corr;

W1ii=Seinv1i^2;

W2ii=Seinv2i^2;

W1i=W1i|W1ii;W2i=W2i|W2ii;

Ssq1=Ssq1|SEma^2;Ssq2=Ssq2|SEtest^2;

i=i+1;

endo;

j=j+1;

endo;

rr=10*(r2+r1);

z1=ones(rr,1);

ave=r'*z1/rr; sum3=sum3+ave;

sumW1=W1i'*z1;sumW2=W2i'*z1;

/* The below program calculates the unrestricted WLS2 & UWLS1 */

bb1=inv(Seinv2'*Seinv2)*Seinv2't2;sum1=sum1+bb1;

s2wls=(t2-Seinv2*bb1)'*(t2-Seinv2*bb1)/(rr-1);

WLSVAR1=s2wls*inv(Seinv1'*Seinv1);MSE3=MSE3+(bb1[1]-sqrt(1/5))^2;

UL1=bb1[1]+2.01*sqrt(WLSVAR1);

LL1=bb1[1]-2.01*sqrt(WLSVAR1);

if LL1<=sqrt(1/5) and UL1>=sqrt(1/5);

cov3=cov3+1;

endif;

bb2=inv(Seinv1'*Seinv1)*Seinv1't1;sum2=sum2+bb2;

s2wls2=(t1-Seinv1*bb2)'*(t1-Seinv1*bb2)/(rr-1);

WLSVAR2=s2wls2*inv(Seinv1'*Seinv1);MSE4=MSE4+(bb2[1]-sqrt(1/5))^2;

UL2=bb2[1]+2.01*sqrt(WLSVAR2);

LL2=bb2[1]-2.01*sqrt(WLSVAR2);

if LL2<=sqrt(1/5) and UL2>=sqrt(1/5);

cov4=cov4+1;

endif;

I2=(s2wls2-1)/s2wls2;

if I2<0;I2=0;endif;sum8=sum8+I2;

/* The below program calculates RE */

Wave1=sumW1/rr;/*see Eq (5.3) in Sutton et al (2000) */

S2w1=(t1'*z1-rr*Wave1^2)/(rr-1);/*Eq (5.4)*/

U=(rr-1)*(Wave1-S2w1/(rr*Wave1));/*Eq (5.5)*/

Q=(W1i'r^2)-(r'*W1i)^2/sumW1;/*Eq (3.2)*/

BSvar=(Q-rr+1)/U;/*Eq (5.6)*/

if Q<=rr-1;

BSvar=0;

endif;

iBS=1; weight={};

do while (iBS<=rr);

weighti=1/(BSvar+Ssq1[iBS]);/*Eq(5.7)*/

weight=weight|weighti;

iBS=iBS+1;

endo;

sumWeight1=weight'*z1;

REWA1=(r'*weight)/sumWeight1; /*Eq(5.8)*/

VARRE=1/sumWeight1;

ulr=REWA1+1.96*sqrt(VARRE);

llr=REWA1-1.96*sqrt(VARRE);

if llr<=sqrt(1/5) and ulr>=sqrt(1/5);

cov1=cov1+1;

endif;

sum5=sum5+REWA1;MSE1=MSE1+(REWA1-sqrt(1/5))^2;

Wave2=sumW2/rr;/*see Eq (5.3) in Sutton et al (2000) */

S2w2=(t2'*z1-rr*Wave2^2)/(rr-1);/*Eq (5.4)*/

U=(rr-1)*(Wave2-S2w2/(rr*Wave2));/*Eq (5.5)*/

Q=(W2i'r^2)-(r'*W2i)^2/sumW2;/*Eq (3.2)*/

BSvar=(Q-rr+1)/U;/*Eq (5.6)*/

if Q<=rr-1;

BSvar=0;

endif;

iBS=1; weight={};

do while (iBS<=rr);

weighti=1/(BSvar+Ssq2[iBS]);/*Eq(5.7)*/

weight=weight|weighti;

iBS=iBS+1;

endo;

sumWeight2=weight'*z1;

REWA2=(r'*weight)/sumWeight2; /*Eq(5.8)*/

VARRE2=1/sumWeight2;

ulr=REWA2+1.96*sqrt(VARRE2);

llr=REWA2-1.96*sqrt(VARRE2);

if llr<=sqrt(1/5) and ulr>=sqrt(1/5);

cov2=cov2+1;

endif;

sum7=sum7+REWA2;MSE2=MSE2+(REWA2-sqrt(1/5))^2;

k=k+1;

endo;

UWLSBias1=(sum1/rep)-sqrt(1/5);UWLScov1=cov3/rep;

UWLSBias2=(sum2/rep)-sqrt(1/5);UWLScov2=cov4/rep;

REBias1=(sum5/rep)-sqrt(1/5);REcov1=cov1/rep;Isq=sum8/rep;

REBias2=(sum7/rep)-sqrt(1/5);REcov2=cov2/rep;

WLSRMSE1=(MSE3/rep)^.5;WLSRMSE2=(MSE4/rep)^.5;

RERMSE1=(MSE1/rep)^.5;RERMSE2=(MSE2/rep)^.5;

AveBias=(sum3/rep)-sqrt(1/5);

screen on;

Isq;;AveBias;;REBias1;;REBias2;;UWLSBias2;;UWLSBias1;;REcov1;;REcov2;;UWLScov2;;UWLScov1;;RERMSE1;;RERMSE2;;WLSRMSE2;;WLSRMSE1;

screen off;

sum1=0;sum2=0;sum3=0;sum10=0;sum11=0;

MSE1=0;MSE2=0;MSE3=0;MSE4=0;

sum4=0;sum5=0;sum6=0;sum7=0;sum9=0;sum8=0;

cov1=0;cov4=0;cov2=0;cov3=0;

k=1;

do while (k<=rep);/* the number of replications*/

Seinv1={};Seinv2={};Seinv3={};Seinv4={};

t1={}; t2={};r={};t3={};t4={};

W1i={};Zf={};

Ssq1={};Ssq3={};

W2i={};W3i={};W4i={};

Ssq2={}; Ssq4={};

/* space holder 1*/

/* space holder 2*/

/* space holder 3*/

r2=5;r1=0;/* the number of research studies is the sum times 10*/

j=1;

do while (j<=r2);

i=1;

do while (i<=10);

X2=rndn(ID[i],1)/4;

/* space holder 4*/

/* space holder 5*/

/* space holder 6*/

/*Bivariate Correlation using regressions' t-values */

X1=ones(ID[i],1);

Y=X1+X2+rndn(ID[i],1);

X=X2;

_olsres=1;

{ nam,m,b,stb,vc,std,sig,cx,rsq,resid,dbw } = ols(0,Y,X);

ti=b[2]/std[2];

corrpart=ti^2+ID[i]-2;corr=ti/sqrt(corrpart);

b1=het[i]*rndn(1,1);/*adds random Het in terms of Cohen's d*/

d1=b1+2*corr/(1-corr^2)^.5;corr=d1/(d1^2+4)^.5;

/* SEtest is the SE for testing whether corr=0*/

SEtest=sqrt((1-corr^2)/(ID[i]-2));

/* SEma is the conventional meta-analysis SE for corr's*/

SEma=(1-corr^2)/sqrt((ID[i]-1));

t1i=corr/SEma;Seinv1i=1/SEma;

t2i=corr/SEtest;Seinv2i=1/SEtest;

Seinv1=Seinv1|Seinv1i;t1=t1|t1i;

Seinv2=Seinv2|Seinv2i;t2=t2|t2i;

r=r|corr;

W1ii=Seinv1i^2;

W2ii=Seinv2i^2;

W1i=W1i|W1ii;W2i=W2i|W2ii;

Ssq1=Ssq1|SEma^2;Ssq2=Ssq2|SEtest^2;

i=i+1;

endo;

j=j+1;

endo;

/* The above program does not select results at all */

/* The below program selects entirely for statistical significance */

j=1;

do while (j<=r1);

i=1;

do while (i<=10);

ti=0;

do until ti>=critt[i];

X2=rndn(ID[i],1)/4;

/*Bivariate Correlation using regressions' t-values */

/* space holder 4*/

/* space holder 5*/

/* space holder 6*/

X1=ones(ID[i],1);

Y=X1+X2+rndn(ID[i],1);

X=X2;

_olsres=1;

{ nam,m,b,stb,vc,std,sig,cx,rsq,resid,dbw } = ols(0,Y,X);

ti=b[2]/std[2];

corrpart=ti^2+ID[i]-2;corr=ti/sqrt(corrpart);

b1=het[i]*rndn(1,1);/*adds random Het in terms of Cohen's d*/

d1=b1+2*corr/(1-corr^2)^.5;corr=d1/(d1^2+4)^.5;

/* SEtest is the SE for testing whether corr=0*/

SEtest=sqrt((1-corr^2)/(ID[i]-2));

ti=corr/SEtest;

endo;

/* SEma is the conventional meta-analysis SE for corr's*/

SEma=(1-corr^2)/sqrt((ID[i]-1));

t1i=corr/SEma;Seinv1i=1/SEma;

t2i=corr/SEtest;Seinv2i=1/SEtest;

Seinv1=Seinv1|Seinv1i;t1=t1|t1i;

Seinv2=Seinv2|Seinv2i;t2=t2|t2i;

r=r|corr;

W1ii=Seinv1i^2;

W2ii=Seinv2i^2;

W1i=W1i|W1ii;W2i=W2i|W2ii;

Ssq1=Ssq1|SEma^2;Ssq2=Ssq2|SEtest^2;

i=i+1;

endo;

j=j+1;

endo;

rr=10*(r2+r1);

z1=ones(rr,1);

ave=r'*z1/rr; sum3=sum3+ave;

sumW1=W1i'*z1;sumW2=W2i'*z1;

/* The below program calculates the unrestricted WLS2 & UWLS1 */

bb1=inv(Seinv2'*Seinv2)*Seinv2't2;sum1=sum1+bb1;

s2wls=(t2-Seinv2*bb1)'*(t2-Seinv2*bb1)/(rr-1);

WLSVAR1=s2wls*inv(Seinv1'*Seinv1);MSE3=MSE3+(bb1[1]-sqrt(1/17))^2;

UL1=bb1[1]+2.01*sqrt(WLSVAR1);

LL1=bb1[1]-2.01*sqrt(WLSVAR1);

if LL1<=sqrt(1/17) and UL1>=sqrt(1/17);

cov3=cov3+1;

endif;

bb2=inv(Seinv1'*Seinv1)*Seinv1't1;sum2=sum2+bb2;

s2wls2=(t1-Seinv1*bb2)'*(t1-Seinv1*bb2)/(rr-1);

WLSVAR2=s2wls2*inv(Seinv1'*Seinv1);MSE4=MSE4+(bb2[1]-sqrt(1/17))^2;

UL2=bb2[1]+2.01*sqrt(WLSVAR2);

LL2=bb2[1]-2.01*sqrt(WLSVAR2);

if LL2<=sqrt(1/17) and UL2>=sqrt(1/17);

cov4=cov4+1;

endif;

I2=(s2wls2-1)/s2wls2;

if I2<0;I2=0;endif;sum8=sum8+I2;

/* The below program calculates RE */

Wave1=sumW1/rr;/*see Eq (5.3) in Sutton et al (2000) */

S2w1=(t1'*z1-rr*Wave1^2)/(rr-1);/*Eq (5.4)*/

U=(rr-1)*(Wave1-S2w1/(rr*Wave1));/*Eq (5.5)*/

Q=(W1i'r^2)-(r'*W1i)^2/sumW1;/*Eq (3.2)*/

BSvar=(Q-rr+1)/U;/*Eq (5.6)*/

if Q<=rr-1;

BSvar=0;

endif;

iBS=1; weight={};

do while (iBS<=rr);

weighti=1/(BSvar+Ssq1[iBS]);/*Eq(5.7)*/

weight=weight|weighti;

iBS=iBS+1;

endo;

sumWeight1=weight'*z1;

REWA1=(r'*weight)/sumWeight1; /*Eq(5.8)*/

VARRE=1/sumWeight1;

ulr=REWA1+1.96*sqrt(VARRE);

llr=REWA1-1.96*sqrt(VARRE);

if llr<=sqrt(1/17) and ulr>=sqrt(1/17);

cov1=cov1+1;

endif;

sum5=sum5+REWA1;MSE1=MSE1+(REWA1-sqrt(1/17))^2;

Wave2=sumW2/rr;/*see Eq (5.3) in Sutton et al (2000) */

S2w2=(t2'*z1-rr*Wave2^2)/(rr-1);/*Eq (5.4)*/

U=(rr-1)*(Wave2-S2w2/(rr*Wave2));/*Eq (5.5)*/

Q=(W2i'r^2)-(r'*W2i)^2/sumW2;/*Eq (3.2)*/

BSvar=(Q-rr+1)/U;/*Eq (5.6)*/

if Q<=rr-1;

BSvar=0;

endif;

iBS=1; weight={};

do while (iBS<=rr);

weighti=1/(BSvar+Ssq2[iBS]);/*Eq(5.7)*/

weight=weight|weighti;

iBS=iBS+1;

endo;

sumWeight2=weight'*z1;

REWA2=(r'*weight)/sumWeight2; /*Eq(5.8)*/

VARRE2=1/sumWeight2;

ulr=REWA2+1.96*sqrt(VARRE2);

llr=REWA2-1.96*sqrt(VARRE2);

if llr<=sqrt(1/17) and ulr>=sqrt(1/17);

cov2=cov2+1;

endif;

sum7=sum7+REWA2;MSE2=MSE2+(REWA2-sqrt(1/17))^2;

k=k+1;

endo;

UWLSBias1=(sum1/rep)-sqrt(1/17);UWLScov1=cov3/rep;

UWLSBias2=(sum2/rep)-sqrt(1/17);UWLScov2=cov4/rep;

REBias1=(sum5/rep)-sqrt(1/17);REcov1=cov1/rep;Isq=sum8/rep;

REBias2=(sum7/rep)-sqrt(1/17);REcov2=cov2/rep;

WLSRMSE1=(MSE3/rep)^.5;WLSRMSE2=(MSE4/rep)^.5;

RERMSE1=(MSE1/rep)^.5;RERMSE2=(MSE2/rep)^.5;

AveBias=(sum3/rep)-sqrt(1/17);

screen on;

Isq;;AveBias;;REBias1;;REBias2;;UWLSBias2;;UWLSBias1;;REcov1;;REcov2;;UWLScov2;;UWLScov1;;RERMSE1;;RERMSE2;;WLSRMSE2;;WLSRMSE1;

screen off;

sum1=0;sum2=0;sum3=0;sum10=0;sum11=0;

MSE1=0;MSE2=0;MSE3=0;MSE4=0;

sum4=0;sum5=0;sum6=0;sum7=0;sum9=0;sum8=0;

cov1=0;cov4=0;cov2=0;cov3=0;

k=1;

do while (k<=rep);/* the number of replications*/

Seinv1={};Seinv2={};Seinv3={};Seinv4={};

t1={}; t2={};r={};t3={};t4={};

W1i={};Zf={};

Ssq1={};Ssq3={};

W2i={};W3i={};W4i={};

Ssq2={}; Ssq4={};

/* space holder 1*/

/* space holder 2*/

/* space holder 3*/

r2=5;r1=0;/* the number of research studies is the sum times 10*/

j=1;

do while (j<=r2);

i=1;

do while (i<=10);

X2=rndn(ID[i],1)/9;

/* space holder 4*/

/* space holder 5*/

/* space holder 6*/

/*Bivariate Correlation using regressions' t-values */

X1=ones(ID[i],1);

Y=X1+X2+rndn(ID[i],1);

X=X2;

_olsres=1;

{ nam,m,b,stb,vc,std,sig,cx,rsq,resid,dbw } = ols(0,Y,X);

ti=b[2]/std[2];

corrpart=ti^2+ID[i]-2;corr=ti/sqrt(corrpart);

b1=het[i]*rndn(1,1);/*adds random Het in terms of Cohen's d*/

d1=b1+2*corr/(1-corr^2)^.5;corr=d1/(d1^2+4)^.5;

/* SEtest is the SE for testing whether corr=0*/

SEtest=sqrt((1-corr^2)/(ID[i]-2));

/* SEma is the conventional meta-analysis SE for corr's*/

SEma=(1-corr^2)/sqrt((ID[i]-1));

t1i=corr/SEma;Seinv1i=1/SEma;

t2i=corr/SEtest;Seinv2i=1/SEtest;

Seinv1=Seinv1|Seinv1i;t1=t1|t1i;

Seinv2=Seinv2|Seinv2i;t2=t2|t2i;

r=r|corr;

W1ii=Seinv1i^2;

W2ii=Seinv2i^2;

W1i=W1i|W1ii;W2i=W2i|W2ii;

Ssq1=Ssq1|SEma^2;Ssq2=Ssq2|SEtest^2;

i=i+1;

endo;

j=j+1;

endo;

/* The above program does not select results at all */

/* The below program selects entirely for statistical significance */

j=1;

do while (j<=r1);

i=1;

do while (i<=10);

ti=0;

do until ti>=critt[i];

X2=rndn(ID[i],1)/9;

/*Bivariate Correlation using regressions' t-values */

/* space holder 4*/

/* space holder 5*/

/* space holder 6*/

X1=ones(ID[i],1);

Y=X1+X2+rndn(ID[i],1);

X=X2;

_olsres=1;

{ nam,m,b,stb,vc,std,sig,cx,rsq,resid,dbw } = ols(0,Y,X);

ti=b[2]/std[2];

corrpart=ti^2+ID[i]-2;corr=ti/sqrt(corrpart);

b1=het[i]*rndn(1,1);/*adds random Het in terms of Cohen's d*/

d1=b1+2*corr/(1-corr^2)^.5;corr=d1/(d1^2+4)^.5;

/* SEtest is the SE for testing whether corr=0*/

SEtest=sqrt((1-corr^2)/(ID[i]-2));

ti=corr/SEtest;

endo;

/* SEma is the conventional meta-analysis SE for corr's*/

SEma=(1-corr^2)/sqrt((ID[i]-1));

t1i=corr/SEma;Seinv1i=1/SEma;

t2i=corr/SEtest;Seinv2i=1/SEtest;

Seinv1=Seinv1|Seinv1i;t1=t1|t1i;

Seinv2=Seinv2|Seinv2i;t2=t2|t2i;

r=r|corr;

W1ii=Seinv1i^2;

W2ii=Seinv2i^2;

W1i=W1i|W1ii;W2i=W2i|W2ii;

Ssq1=Ssq1|SEma^2;Ssq2=Ssq2|SEtest^2;

i=i+1;

endo;

j=j+1;

endo;

rr=10*(r2+r1);

z1=ones(rr,1);

ave=r'*z1/rr; sum3=sum3+ave;

sumW1=W1i'*z1;sumW2=W2i'*z1;

/* The below program calculates the unrestricted WLS2 & UWLS1 */

bb1=inv(Seinv2'*Seinv2)*Seinv2't2;sum1=sum1+bb1;

s2wls=(t2-Seinv2*bb1)'*(t2-Seinv2*bb1)/(rr-1);

WLSVAR1=s2wls*inv(Seinv1'*Seinv1);MSE3=MSE3+(bb1[1]-sqrt(1/82))^2;

UL1=bb1[1]+2.01*sqrt(WLSVAR1);

LL1=bb1[1]-2.01*sqrt(WLSVAR1);

if LL1<=sqrt(1/82) and UL1>=sqrt(1/82);

cov3=cov3+1;

endif;

bb2=inv(Seinv1'*Seinv1)*Seinv1't1;sum2=sum2+bb2;

s2wls2=(t1-Seinv1*bb2)'*(t1-Seinv1*bb2)/(rr-1);

WLSVAR2=s2wls2*inv(Seinv1'*Seinv1);MSE4=MSE4+(bb2[1]-sqrt(1/82))^2;

UL2=bb2[1]+2.01*sqrt(WLSVAR2);

LL2=bb2[1]-2.01*sqrt(WLSVAR2);

if LL2<=sqrt(1/82) and UL2>=sqrt(1/82);

cov4=cov4+1;

endif;

I2=(s2wls2-1)/s2wls2;

if I2<0;I2=0;endif;sum8=sum8+I2;

/* The below program calculates RE */

Wave1=sumW1/rr;/*see Eq (5.3) in Sutton et al (2000) */

S2w1=(t1'*z1-rr*Wave1^2)/(rr-1);/*Eq (5.4)*/

U=(rr-1)*(Wave1-S2w1/(rr*Wave1));/*Eq (5.5)*/

Q=(W1i'r^2)-(r'*W1i)^2/sumW1;/*Eq (3.2)*/

BSvar=(Q-rr+1)/U;/*Eq (5.6)*/

if Q<=rr-1;

BSvar=0;

endif;

iBS=1; weight={};

do while (iBS<=rr);

weighti=1/(BSvar+Ssq1[iBS]);/*Eq(5.7)*/

weight=weight|weighti;

iBS=iBS+1;

endo;

sumWeight1=weight'*z1;

REWA1=(r'*weight)/sumWeight1; /*Eq(5.8)*/

VARRE=1/sumWeight1;

ulr=REWA1+1.96*sqrt(VARRE);

llr=REWA1-1.96*sqrt(VARRE);

if llr<=sqrt(1/82) and ulr>=sqrt(1/82);

cov1=cov1+1;

endif;

sum5=sum5+REWA1;MSE1=MSE1+(REWA1-sqrt(1/82))^2;

Wave2=sumW2/rr;/*see Eq (5.3) in Sutton et al (2000) */

S2w2=(t2'*z1-rr*Wave2^2)/(rr-1);/*Eq (5.4)*/

U=(rr-1)*(Wave2-S2w2/(rr*Wave2));/*Eq (5.5)*/

Q=(W2i'r^2)-(r'*W2i)^2/sumW2;/*Eq (3.2)*/

BSvar=(Q-rr+1)/U;/*Eq (5.6)*/

if Q<=rr-1;

BSvar=0;

endif;

iBS=1; weight={};

do while (iBS<=rr);

weighti=1/(BSvar+Ssq2[iBS]);/*Eq(5.7)*/

weight=weight|weighti;

iBS=iBS+1;

endo;

sumWeight2=weight'*z1;

REWA2=(r'*weight)/sumWeight2; /*Eq(5.8)*/

VARRE2=1/sumWeight2;

ulr=REWA2+1.96*sqrt(VARRE2);

llr=REWA2-1.96*sqrt(VARRE2);

if llr<=sqrt(1/82) and ulr>=sqrt(1/82);

cov2=cov2+1;

endif;

sum7=sum7+REWA2;MSE2=MSE2+(REWA2-sqrt(1/82))^2;

k=k+1;

endo;

UWLSBias1=(sum1/rep)-sqrt(1/82);UWLScov1=cov3/rep;

UWLSBias2=(sum2/rep)-sqrt(1/82);UWLScov2=cov4/rep;

REBias1=(sum5/rep)-sqrt(1/82);REcov1=cov1/rep;Isq=sum8/rep;

REBias2=(sum7/rep)-sqrt(1/82);REcov2=cov2/rep;

WLSRMSE1=(MSE3/rep)^.5;WLSRMSE2=(MSE4/rep)^.5;

RERMSE1=(MSE1/rep)^.5;RERMSE2=(MSE2/rep)^.5;

AveBias=(sum3/rep)-sqrt(1/82);

screen on;

Isq;;AveBias;;REBias1;;REBias2;;UWLSBias2;;UWLSBias1;;REcov1;;REcov2;;UWLScov2;;UWLScov1;;RERMSE1;;RERMSE2;;WLSRMSE2;;WLSRMSE1;

screen off;

sum1=0;sum2=0;sum3=0;sum10=0;sum11=0;

MSE1=0;MSE2=0;MSE3=0;MSE4=0;

sum4=0;sum5=0;sum6=0;sum7=0;sum9=0;sum8=0;

cov1=0;cov4=0;cov2=0;cov3=0;

k=1;

do while (k<=rep);/* the number of replications*/

Seinv1={};Seinv2={};Seinv3={};Seinv4={};

t1={}; t2={};r={};t3={};t4={};

W1i={};Zf={};

Ssq1={};Ssq3={};

W2i={};W3i={};W4i={};

Ssq2={}; Ssq4={};

/* space holder 1*/

/* space holder 2*/

/* space holder 3*/

r2=5;r1=0;/* the number of research studies is the sum times 10*/

j=1;

do while (j<=r2);

i=1;

do while (i<=10);

PB=rndu(1,1);

if PB <= .5;

X2=rndn(ID[i],1)/2;

/* space holder 4*/

/* space holder 5*/

/* space holder 6*/

/*Bivariate Correlation using regressions' t-values */

X1=ones(ID[i],1);

Y=X1+X2+rndn(ID[i],1);

X=X2;

_olsres=1;

{ nam,m,b,stb,vc,std,sig,cx,rsq,resid,dbw } = ols(0,Y,X);

ti=b[2]/std[2];

corrpart=ti^2+ID[i]-2;corr=ti/sqrt(corrpart);

b1=het[i]*rndn(1,1);/*adds random Het in terms of Cohen's d*/

d1=b1+2*corr/(1-corr^2)^.5;corr=d1/(d1^2+4)^.5;

/* SEtest is the SE for testing whether corr=0*/

SEtest=sqrt((1-corr^2)/(ID[i]-2));

/* SEma is the conventional meta-analysis SE for corr's*/

SEma=(1-corr^2)/sqrt((ID[i]-1));

t1i=corr/SEma;Seinv1i=1/SEma;

t2i=corr/SEtest;Seinv2i=1/SEtest;

Seinv1=Seinv1|Seinv1i;t1=t1|t1i;

Seinv2=Seinv2|Seinv2i;t2=t2|t2i;

r=r|corr;

W1ii=Seinv1i^2;

W2ii=Seinv2i^2;

W1i=W1i|W1ii;W2i=W2i|W2ii;

Ssq1=Ssq1|SEma^2;Ssq2=Ssq2|SEtest^2;

/* The below program selects entirely for statistical significance */

else;

ti=0;

do until ti>=critt[i];

X2=rndn(ID[i],1)/2;

/*Bivariate Correlation using regressions' t-values */

/* space holder 4*/

/* space holder 5*/

/* space holder 6*/

X1=ones(ID[i],1);

Y=X1+X2+rndn(ID[i],1);

X=X2;

_olsres=1;

{ nam,m,b,stb,vc,std,sig,cx,rsq,resid,dbw } = ols(0,Y,X);

ti=b[2]/std[2];

corrpart=ti^2+ID[i]-2;corr=ti/sqrt(corrpart);

b1=het[i]*rndn(1,1);/*adds random Het in terms of Cohen's d*/

d1=b1+2*corr/(1-corr^2)^.5;corr=d1/(d1^2+4)^.5;

/* SEtest is the SE for testing whether corr=0*/

SEtest=sqrt((1-corr^2)/(ID[i]-2));

ti=corr/SEtest;

endo;

/* SEma is the conventional meta-analysis SE for corr's*/

SEma=(1-corr^2)/sqrt((ID[i]-1));

t1i=corr/SEma;Seinv1i=1/SEma;

t2i=corr/SEtest;Seinv2i=1/SEtest;

Seinv1=Seinv1|Seinv1i;t1=t1|t1i;

Seinv2=Seinv2|Seinv2i;t2=t2|t2i;

r=r|corr;

W1ii=Seinv1i^2;

W2ii=Seinv2i^2;

W1i=W1i|W1ii;W2i=W2i|W2ii;

Ssq1=Ssq1|SEma^2;Ssq2=Ssq2|SEtest^2;

endif;

i=i+1;

endo;

j=j+1;

endo;

rr=10*(r2+r1);

z1=ones(rr,1);

ave=r'*z1/rr; sum3=sum3+ave;

sumW1=W1i'*z1;sumW2=W2i'*z1;

/* The below program calculates the unrestricted WLS2 & UWLS1 */

bb1=inv(Seinv2'*Seinv2)*Seinv2't2;sum1=sum1+bb1;

s2wls=(t2-Seinv2*bb1)'*(t2-Seinv2*bb1)/(rr-1);

WLSVAR1=s2wls*inv(Seinv1'*Seinv1);MSE3=MSE3+(bb1[1]-sqrt(1/5))^2;

UL1=bb1[1]+2.01*sqrt(WLSVAR1);

LL1=bb1[1]-2.01*sqrt(WLSVAR1);

if LL1<=sqrt(1/5) and UL1>=sqrt(1/5);

cov3=cov3+1;

endif;

bb2=inv(Seinv1'*Seinv1)*Seinv1't1;sum2=sum2+bb2;

s2wls2=(t1-Seinv1*bb2)'*(t1-Seinv1*bb2)/(rr-1);

WLSVAR2=s2wls2*inv(Seinv1'*Seinv1);MSE4=MSE4+(bb2[1]-sqrt(1/5))^2;

UL2=bb2[1]+2.01*sqrt(WLSVAR2);

LL2=bb2[1]-2.01*sqrt(WLSVAR2);

if LL2<=sqrt(1/5) and UL2>=sqrt(1/5);

cov4=cov4+1;

endif;

I2=(s2wls2-1)/s2wls2;

if I2<0;I2=0;endif;sum8=sum8+I2;

/* The below program calculates RE */

Wave1=sumW1/rr;/*see Eq (5.3) in Sutton et al (2000) */

S2w1=(t1'*z1-rr*Wave1^2)/(rr-1);/*Eq (5.4)*/

U=(rr-1)*(Wave1-S2w1/(rr*Wave1));/*Eq (5.5)*/

Q=(W1i'r^2)-(r'*W1i)^2/sumW1;/*Eq (3.2)*/

BSvar=(Q-rr+1)/U;/*Eq (5.6)*/

if Q<=rr-1;

BSvar=0;

endif;

iBS=1; weight={};

do while (iBS<=rr);

weighti=1/(BSvar+Ssq1[iBS]);/*Eq(5.7)*/

weight=weight|weighti;

iBS=iBS+1;

endo;

sumWeight1=weight'*z1;

REWA1=(r'*weight)/sumWeight1; /*Eq(5.8)*/

VARRE=1/sumWeight1;

ulr=REWA1+1.96*sqrt(VARRE);

llr=REWA1-1.96*sqrt(VARRE);

if llr<=sqrt(1/5) and ulr>=sqrt(1/5);

cov1=cov1+1;

endif;

sum5=sum5+REWA1;MSE1=MSE1+(REWA1-sqrt(1/5))^2;

Wave2=sumW2/rr;/*see Eq (5.3) in Sutton et al (2000) */

S2w2=(t2'*z1-rr*Wave2^2)/(rr-1);/*Eq (5.4)*/

U=(rr-1)*(Wave2-S2w2/(rr*Wave2));/*Eq (5.5)*/

Q=(W2i'r^2)-(r'*W2i)^2/sumW2;/*Eq (3.2)*/

BSvar=(Q-rr+1)/U;/*Eq (5.6)*/

if Q<=rr-1;

BSvar=0;

endif;

iBS=1; weight={};

do while (iBS<=rr);

weighti=1/(BSvar+Ssq2[iBS]);/*Eq(5.7)*/

weight=weight|weighti;

iBS=iBS+1;

endo;

sumWeight2=weight'*z1;

REWA2=(r'*weight)/sumWeight2; /*Eq(5.8)*/

VARRE2=1/sumWeight2;

ulr=REWA2+1.96*sqrt(VARRE2);

llr=REWA2-1.96*sqrt(VARRE2);

if llr<=sqrt(1/5) and ulr>=sqrt(1/5);

cov2=cov2+1;

endif;

sum7=sum7+REWA2;MSE2=MSE2+(REWA2-sqrt(1/5))^2;

k=k+1;

endo;

UWLSBias1=(sum1/rep)-sqrt(1/5);UWLScov1=cov3/rep;

UWLSBias2=(sum2/rep)-sqrt(1/5);UWLScov2=cov4/rep;

REBias1=(sum5/rep)-sqrt(1/5);REcov1=cov1/rep;Isq=sum8/rep;

REBias2=(sum7/rep)-sqrt(1/5);REcov2=cov2/rep;

WLSRMSE1=(MSE3/rep)^.5;WLSRMSE2=(MSE4/rep)^.5;

RERMSE1=(MSE1/rep)^.5;RERMSE2=(MSE2/rep)^.5;

AveBias=(sum3/rep)-sqrt(1/5);

screen on;

Isq;;AveBias;;REBias1;;REBias2;;UWLSBias2;;UWLSBias1;;REcov1;;REcov2;;UWLScov2;;UWLScov1;;RERMSE1;;RERMSE2;;WLSRMSE2;;WLSRMSE1;

screen off;

sum1=0;sum2=0;sum3=0;sum10=0;sum11=0;

MSE1=0;MSE2=0;MSE3=0;MSE4=0;

sum4=0;sum5=0;sum6=0;sum7=0;sum9=0;sum8=0;

cov1=0;cov4=0;cov2=0;cov3=0;

k=1;

do while (k<=rep);/* the number of replications*/

Seinv1={};Seinv2={};Seinv3={};Seinv4={};

t1={}; t2={};r={};t3={};t4={};

W1i={};Zf={};

Ssq1={};Ssq3={};

W2i={};W3i={};W4i={};

Ssq2={}; Ssq4={};

/* space holder 1*/

/* space holder 2*/

/* space holder 3*/

r2=5;r1=0;/* the number of research studies is the sum times 10*/

j=1;

do while (j<=r2);

i=1;

do while (i<=10);

PB=rndu(1,1);

if PB <= .5;

X2=rndn(ID[i],1)/4;

/* space holder 4*/

/* space holder 5*/

/* space holder 6*/

/*Bivariate Correlation using regressions' t-values */

X1=ones(ID[i],1);

Y=X1+X2+rndn(ID[i],1);

X=X2;

_olsres=1;

{ nam,m,b,stb,vc,std,sig,cx,rsq,resid,dbw } = ols(0,Y,X);

ti=b[2]/std[2];

corrpart=ti^2+ID[i]-2;corr=ti/sqrt(corrpart);

b1=het[i]*rndn(1,1);/*adds random Het in terms of Cohen's d*/

d1=b1+2*corr/(1-corr^2)^.5;corr=d1/(d1^2+4)^.5;

/* SEtest is the SE for testing whether corr=0*/

SEtest=sqrt((1-corr^2)/(ID[i]-2));

/* SEma is the conventional meta-analysis SE for corr's*/

SEma=(1-corr^2)/sqrt((ID[i]-1));

t1i=corr/SEma;Seinv1i=1/SEma;

t2i=corr/SEtest;Seinv2i=1/SEtest;

Seinv1=Seinv1|Seinv1i;t1=t1|t1i;

Seinv2=Seinv2|Seinv2i;t2=t2|t2i;

r=r|corr;

W1ii=Seinv1i^2;

W2ii=Seinv2i^2;

W1i=W1i|W1ii;W2i=W2i|W2ii;

Ssq1=Ssq1|SEma^2;Ssq2=Ssq2|SEtest^2;

/* The below program selects entirely for statistical significance */

else;

ti=0;

do until ti>=critt[i];

X2=rndn(ID[i],1)/4;

/*Bivariate Correlation using regressions' t-values */

/* space holder 4*/

/* space holder 5*/

/* space holder 6*/

X1=ones(ID[i],1);

Y=X1+X2+rndn(ID[i],1);

X=X2;

_olsres=1;

{ nam,m,b,stb,vc,std,sig,cx,rsq,resid,dbw } = ols(0,Y,X);

ti=b[2]/std[2];

corrpart=ti^2+ID[i]-2;corr=ti/sqrt(corrpart);

b1=het[i]*rndn(1,1);/*adds random Het in terms of Cohen's d*/

d1=b1+2*corr/(1-corr^2)^.5;corr=d1/(d1^2+4)^.5;

/* SEtest is the SE for testing whether corr=0*/

SEtest=sqrt((1-corr^2)/(ID[i]-2));

ti=corr/SEtest;

endo;

/* SEma is the conventional meta-analysis SE for corr's*/

SEma=(1-corr^2)/sqrt((ID[i]-1));

t1i=corr/SEma;Seinv1i=1/SEma;

t2i=corr/SEtest;Seinv2i=1/SEtest;

Seinv1=Seinv1|Seinv1i;t1=t1|t1i;

Seinv2=Seinv2|Seinv2i;t2=t2|t2i;

r=r|corr;

W1ii=Seinv1i^2;

W2ii=Seinv2i^2;

W1i=W1i|W1ii;W2i=W2i|W2ii;

Ssq1=Ssq1|SEma^2;Ssq2=Ssq2|SEtest^2;

endif;

i=i+1;

endo;

j=j+1;

endo;

rr=10*(r2+r1);

z1=ones(rr,1);

ave=r'*z1/rr; sum3=sum3+ave;

sumW1=W1i'*z1;sumW2=W2i'*z1;

/* The below program calculates the unrestricted WLS2 & UWLS1 */

bb1=inv(Seinv2'*Seinv2)*Seinv2't2;sum1=sum1+bb1;

s2wls=(t2-Seinv2*bb1)'*(t2-Seinv2*bb1)/(rr-1);

WLSVAR1=s2wls*inv(Seinv1'*Seinv1);MSE3=MSE3+(bb1[1]-sqrt(1/17))^2;

UL1=bb1[1]+2.01*sqrt(WLSVAR1);

LL1=bb1[1]-2.01*sqrt(WLSVAR1);

if LL1<=sqrt(1/17) and UL1>=sqrt(1/17);

cov3=cov3+1;

endif;

bb2=inv(Seinv1'*Seinv1)*Seinv1't1;sum2=sum2+bb2;

s2wls2=(t1-Seinv1*bb2)'*(t1-Seinv1*bb2)/(rr-1);

WLSVAR2=s2wls2*inv(Seinv1'*Seinv1);MSE4=MSE4+(bb2[1]-sqrt(1/17))^2;

UL2=bb2[1]+2.01*sqrt(WLSVAR2);

LL2=bb2[1]-2.01*sqrt(WLSVAR2);

if LL2<=sqrt(1/17) and UL2>=sqrt(1/17);

cov4=cov4+1;

endif;

I2=(s2wls2-1)/s2wls2;

if I2<0;I2=0;endif;sum8=sum8+I2;

/* The below program calculates RE */

Wave1=sumW1/rr;/*see Eq (5.3) in Sutton et al (2000) */

S2w1=(t1'*z1-rr*Wave1^2)/(rr-1);/*Eq (5.4)*/

U=(rr-1)*(Wave1-S2w1/(rr*Wave1));/*Eq (5.5)*/

Q=(W1i'r^2)-(r'*W1i)^2/sumW1;/*Eq (3.2)*/

BSvar=(Q-rr+1)/U;/*Eq (5.6)*/

if Q<=rr-1;

BSvar=0;

endif;

iBS=1; weight={};

do while (iBS<=rr);

weighti=1/(BSvar+Ssq1[iBS]);/*Eq(5.7)*/

weight=weight|weighti;

iBS=iBS+1;

endo;

sumWeight1=weight'*z1;

REWA1=(r'*weight)/sumWeight1; /*Eq(5.8)*/

VARRE=1/sumWeight1;

ulr=REWA1+1.96*sqrt(VARRE);

llr=REWA1-1.96*sqrt(VARRE);

if llr<=sqrt(1/17) and ulr>=sqrt(1/17);

cov1=cov1+1;

endif;

sum5=sum5+REWA1;MSE1=MSE1+(REWA1-sqrt(1/17))^2;

Wave2=sumW2/rr;/*see Eq (5.3) in Sutton et al (2000) */

S2w2=(t2'*z1-rr*Wave2^2)/(rr-1);/*Eq (5.4)*/

U=(rr-1)*(Wave2-S2w2/(rr*Wave2));/*Eq (5.5)*/

Q=(W2i'r^2)-(r'*W2i)^2/sumW2;/*Eq (3.2)*/

BSvar=(Q-rr+1)/U;/*Eq (5.6)*/

if Q<=rr-1;

BSvar=0;

endif;

iBS=1; weight={};

do while (iBS<=rr);

weighti=1/(BSvar+Ssq2[iBS]);/*Eq(5.7)*/

weight=weight|weighti;

iBS=iBS+1;

endo;

sumWeight2=weight'*z1;

REWA2=(r'*weight)/sumWeight2; /*Eq(5.8)*/

VARRE2=1/sumWeight2;

ulr=REWA2+1.96*sqrt(VARRE2);

llr=REWA2-1.96*sqrt(VARRE2);

if llr<=sqrt(1/17) and ulr>=sqrt(1/17);

cov2=cov2+1;

endif;

sum7=sum7+REWA2;MSE2=MSE2+(REWA2-sqrt(1/17))^2;

k=k+1;

endo;

UWLSBias1=(sum1/rep)-sqrt(1/17);UWLScov1=cov3/rep;

UWLSBias2=(sum2/rep)-sqrt(1/17);UWLScov2=cov4/rep;

REBias1=(sum5/rep)-sqrt(1/17);REcov1=cov1/rep;Isq=sum8/rep;

REBias2=(sum7/rep)-sqrt(1/17);REcov2=cov2/rep;

WLSRMSE1=(MSE3/rep)^.5;WLSRMSE2=(MSE4/rep)^.5;

RERMSE1=(MSE1/rep)^.5;RERMSE2=(MSE2/rep)^.5;

AveBias=(sum3/rep)-sqrt(1/17);

screen on;

Isq;;AveBias;;REBias1;;REBias2;;UWLSBias2;;UWLSBias1;;REcov1;;REcov2;;UWLScov2;;UWLScov1;;RERMSE1;;RERMSE2;;WLSRMSE2;;WLSRMSE1;

screen off;

sum1=0;sum2=0;sum3=0;sum10=0;sum11=0;

MSE1=0;MSE2=0;MSE3=0;MSE4=0;

sum4=0;sum5=0;sum6=0;sum7=0;sum9=0;sum8=0;

cov1=0;cov4=0;cov2=0;cov3=0;

k=1;

do while (k<=rep);/* the number of replications*/

Seinv1={};Seinv2={};Seinv3={};Seinv4={};

t1={}; t2={};r={};t3={};t4={};

W1i={};Zf={};

Ssq1={};Ssq3={};

W2i={};W3i={};W4i={};

Ssq2={}; Ssq4={};

/* space holder 1*/

/* space holder 2*/

/* space holder 3*/

r2=5;r1=0;/* the number of research studies is the sum times 10*/

j=1;

do while (j<=r2);

i=1;

do while (i<=10);

PB=rndu(1,1);

if PB <= .5;

X2=rndn(ID[i],1)/9;

/* space holder 4*/

/* space holder 5*/

/* space holder 6*/

/*Bivariate Correlation using regressions' t-values */

X1=ones(ID[i],1);

Y=X1+X2+rndn(ID[i],1);

X=X2;

_olsres=1;

{ nam,m,b,stb,vc,std,sig,cx,rsq,resid,dbw } = ols(0,Y,X);

ti=b[2]/std[2];

corrpart=ti^2+ID[i]-2;corr=ti/sqrt(corrpart);

b1=het[i]*rndn(1,1);/*adds random Het in terms of Cohen's d*/

d1=b1+2*corr/(1-corr^2)^.5;corr=d1/(d1^2+4)^.5;

/* SEtest is the SE for testing whether corr=0*/

SEtest=sqrt((1-corr^2)/(ID[i]-2));

/* SEma is the conventional meta-analysis SE for corr's*/

SEma=(1-corr^2)/sqrt((ID[i]-1));

t1i=corr/SEma;Seinv1i=1/SEma;

t2i=corr/SEtest;Seinv2i=1/SEtest;

Seinv1=Seinv1|Seinv1i;t1=t1|t1i;

Seinv2=Seinv2|Seinv2i;t2=t2|t2i;

r=r|corr;

W1ii=Seinv1i^2;

W2ii=Seinv2i^2;

W1i=W1i|W1ii;W2i=W2i|W2ii;

Ssq1=Ssq1|SEma^2;Ssq2=Ssq2|SEtest^2;

/* The below program selects entirely for statistical significance */

else;

ti=0;

do until ti>=critt[i];

X2=rndn(ID[i],1)/9;

/*Bivariate Correlation using regressions' t-values */

/* space holder 4*/

/* space holder 5*/

/* space holder 6*/

X1=ones(ID[i],1);

Y=X1+X2+rndn(ID[i],1);

X=X2;

_olsres=1;

{ nam,m,b,stb,vc,std,sig,cx,rsq,resid,dbw } = ols(0,Y,X);

ti=b[2]/std[2];

corrpart=ti^2+ID[i]-2;corr=ti/sqrt(corrpart);

b1=het[i]*rndn(1,1);/*adds random Het in terms of Cohen's d*/

d1=b1+2*corr/(1-corr^2)^.5;corr=d1/(d1^2+4)^.5;

/* SEtest is the SE for testing whether corr=0*/

SEtest=sqrt((1-corr^2)/(ID[i]-2));

ti=corr/SEtest;

endo;

/* SEma is the conventional meta-analysis SE for corr's*/

SEma=(1-corr^2)/sqrt((ID[i]-1));

t1i=corr/SEma;Seinv1i=1/SEma;

t2i=corr/SEtest;Seinv2i=1/SEtest;

Seinv1=Seinv1|Seinv1i;t1=t1|t1i;

Seinv2=Seinv2|Seinv2i;t2=t2|t2i;

r=r|corr;

W1ii=Seinv1i^2;

W2ii=Seinv2i^2;

W1i=W1i|W1ii;W2i=W2i|W2ii;

Ssq1=Ssq1|SEma^2;Ssq2=Ssq2|SEtest^2;

endif;

i=i+1;

endo;

j=j+1;

endo;

rr=10*(r2+r1);

z1=ones(rr,1);

ave=r'*z1/rr; sum3=sum3+ave;

sumW1=W1i'*z1;sumW2=W2i'*z1;

/* The below program calculates the unrestricted WLS2 & UWLS1 */

bb1=inv(Seinv2'*Seinv2)*Seinv2't2;sum1=sum1+bb1;

s2wls=(t2-Seinv2*bb1)'*(t2-Seinv2*bb1)/(rr-1);

WLSVAR1=s2wls*inv(Seinv1'*Seinv1);MSE3=MSE3+(bb1[1]-sqrt(1/82))^2;

UL1=bb1[1]+2.01*sqrt(WLSVAR1);

LL1=bb1[1]-2.01*sqrt(WLSVAR1);

if LL1<=sqrt(1/82) and UL1>=sqrt(1/82);

cov3=cov3+1;

endif;

bb2=inv(Seinv1'*Seinv1)*Seinv1't1;sum2=sum2+bb2;

s2wls2=(t1-Seinv1*bb2)'*(t1-Seinv1*bb2)/(rr-1);

WLSVAR2=s2wls2*inv(Seinv1'*Seinv1);MSE4=MSE4+(bb2[1]-sqrt(1/82))^2;

UL2=bb2[1]+2.01*sqrt(WLSVAR2);

LL2=bb2[1]-2.01*sqrt(WLSVAR2);

if LL2<=sqrt(1/82) and UL2>=sqrt(1/82);

cov4=cov4+1;

endif;

I2=(s2wls2-1)/s2wls2;

if I2<0;I2=0;endif;sum8=sum8+I2;

/* The below program calculates RE */

Wave1=sumW1/rr;/*see Eq (5.3) in Sutton et al (2000) */

S2w1=(t1'*z1-rr*Wave1^2)/(rr-1);/*Eq (5.4)*/

U=(rr-1)*(Wave1-S2w1/(rr*Wave1));/*Eq (5.5)*/

Q=(W1i'r^2)-(r'*W1i)^2/sumW1;/*Eq (3.2)*/

BSvar=(Q-rr+1)/U;/*Eq (5.6)*/

if Q<=rr-1;

BSvar=0;

endif;

iBS=1; weight={};

do while (iBS<=rr);

weighti=1/(BSvar+Ssq1[iBS]);/*Eq(5.7)*/

weight=weight|weighti;

iBS=iBS+1;

endo;

sumWeight1=weight'*z1;

REWA1=(r'*weight)/sumWeight1; /*Eq(5.8)*/

VARRE=1/sumWeight1;

ulr=REWA1+1.96*sqrt(VARRE);

llr=REWA1-1.96*sqrt(VARRE);

if llr<=sqrt(1/82) and ulr>=sqrt(1/82);

cov1=cov1+1;

endif;

sum5=sum5+REWA1;MSE1=MSE1+(REWA1-sqrt(1/82))^2;

Wave2=sumW2/rr;/*see Eq (5.3) in Sutton et al (2000) */

S2w2=(t2'*z1-rr*Wave2^2)/(rr-1);/*Eq (5.4)*/

U=(rr-1)*(Wave2-S2w2/(rr*Wave2));/*Eq (5.5)*/

Q=(W2i'r^2)-(r'*W2i)^2/sumW2;/*Eq (3.2)*/

BSvar=(Q-rr+1)/U;/*Eq (5.6)*/

if Q<=rr-1;

BSvar=0;

endif;

iBS=1; weight={};

do while (iBS<=rr);

weighti=1/(BSvar+Ssq2[iBS]);/*Eq(5.7)*/

weight=weight|weighti;

iBS=iBS+1;

endo;

sumWeight2=weight'*z1;

REWA2=(r'*weight)/sumWeight2; /*Eq(5.8)*/

VARRE2=1/sumWeight2;

ulr=REWA2+1.96*sqrt(VARRE2);

llr=REWA2-1.96*sqrt(VARRE2);

if llr<=sqrt(1/82) and ulr>=sqrt(1/82);

cov2=cov2+1;

endif;

sum7=sum7+REWA2;MSE2=MSE2+(REWA2-sqrt(1/82))^2;

k=k+1;

endo;

UWLSBias1=(sum1/rep)-sqrt(1/82);UWLScov1=cov3/rep;

UWLSBias2=(sum2/rep)-sqrt(1/82);UWLScov2=cov4/rep;

REBias1=(sum5/rep)-sqrt(1/82);REcov1=cov1/rep;Isq=sum8/rep;

REBias2=(sum7/rep)-sqrt(1/82);REcov2=cov2/rep;

WLSRMSE1=(MSE3/rep)^.5;WLSRMSE2=(MSE4/rep)^.5;

RERMSE1=(MSE1/rep)^.5;RERMSE2=(MSE2/rep)^.5;

AveBias=(sum3/rep)-sqrt(1/82);

screen on;

Isq;;AveBias;;REBias1;;REBias2;;UWLSBias2;;UWLSBias1;;REcov1;;REcov2;;UWLScov2;;UWLScov1;;RERMSE1;;RERMSE2;;WLSRMSE2;;WLSRMSE1;

screen off;

**Table 2:**

/************************************************************************/

/* PROGRAM: Correlations with different n, het, & pub'bias */

/* AUTHOR: Tom Stanley 12:50 PM 08-Mar-2024 */

/************************************************************************/

new;

screen off;

rep=10000;/*the number of replications*/

sum1=0;sum2=0;sum3=0;sum10=0;sum11=0;

MSE1=0;MSE2=0;MSE3=0;MSE4=0;

sum4=0;sum5=0;sum6=0;sum7=0;sum9=0;sum8=0;

cov1=0;cov4=0;cov2=0;cov3=0;

ID={30,40,50,75,100,100,125,160,200,400};/* sample size of the original studies*/

het={.45,.45,.3,.3,.3,.3,.3,.3,.075,.075};

critt={2.048,2.024,2.011,1.993,1.984,1.984,1.98,1.978,1.975,1.97};

k=1;

do while (k<=rep);/* the number of replications*/

Seinv1={};Seinv2={};Seinv3={};Seinv4={};

t1={}; t2={};r={};t3={};

W1i={};Zf={};Se={};t4={};

Ssq1={};Ssq3={};Sez={};

W2i={};W3i={};W4i={};

Ssq2={}; Ssq4={};

/* The below generates random subject data for each of 50 studies*/

/*Bivariate Correlation using regressions' t-values */

r2=5;/* the number of research studies is the sum times 10*/

j=1;

do while (j<=r2);

i=1;

do while (i<=10);

X2=rndn(ID[i],1)/2;

/* space holder 4*/

/* space holder 5*/

/* space holder 6*/

/*Bivariate Correlation using regressions' t-values */

X1=ones(ID[i],1);

Y=X1+X2+rndn(ID[i],1);

X=X2;

_olsres=1;

{ nam,m,b,stb,vc,std,sig,cx,rsq,resid,dbw } = ols(0,Y,X);

ti=b[2]/std[2];/*corr is conventional corr, corr for UWLS3*/

corr=ti/(ti^2+ID[i]-2)^.5;cor=ti/(ti^2+ID[i]+1)^.5;

b1=0;/*adds random Het in terms of Cohen's d*/

d1=b1+2*corr/(1-corr^2)^.5;corr=d1/(d1^2+4)^.5;

d2=b1+2*cor/(1-cor^2)^.5;cor=d2/(d2^2+4)^.5;

Zfi=.5*ln((1+corr)/(1-corr));/* Fisher's z*/

r=r|corr;Zf=Zf|Zfi;

/* SEtest is the SE for testing whether corr=0*/

SEtest=sqrt((1-corr^2)/(ID[i]-2));

/* SEma is the conventional meta-analysis SE for corr's*/

SEma=(1-corr^2)/sqrt((ID[i]-1));

/* SE3 is the SE for UWLS+3*/

SE3=sqrt((1-cor^2)/(ID[i]+1));

Se=Se|SEtest;

/* SE4 is the SE for Fisher's z*/

SE4=1/sqrt(ID[i]-3);Sez=Sez|SE4;

t1i=corr/SEma;Seinv1i=1/SEma;

t2i=corr/SEtest;Seinv2i=1/SEtest;

Seinv1=Seinv1|Seinv1i;t1=t1|t1i;

Seinv2=Seinv2|Seinv2i;t2=t2|t2i;

t3i=cor/SE3;Seinv3i=1/SE3;

t4i=Zfi/SE4;Seinv4i=1/SE4;

Seinv3=Seinv3|Seinv3i;t3=t3|t3i;

Seinv4=Seinv4|Seinv4i;t4=t4|t4i;

W4ii=Seinv4i^2;

W4i=W4i|W4ii;

Ssq4=Ssq4|SE4^2;

i=i+1;

endo;

j=j+1;

endo;

rr=10*r2;

z1=ones(rr,1);n=W4i+(3*z1);

ave=r'*z1/rr; sum3=sum3+ave;

sumW4=W4i'*z1;

/* The below program calculates UWLS+3 & UWLS1 for I-sq */

bb1=inv(Seinv3'*Seinv3)*Seinv3't3;sum1=sum1+bb1;

s2wls=(t3-Seinv3*bb1)'*(t3-Seinv3*bb1)/(rr-1);

WLSVAR1=s2wls*inv(Seinv3'*Seinv3);MSE3=MSE3+(bb1[1]-sqrt(1/5))^2;

UL1=bb1[1]+2.01*sqrt(WLSVAR1);

LL1=bb1[1]-2.01*sqrt(WLSVAR1);

if LL1<=sqrt(1/5) and UL1>=sqrt(1/5);

cov3=cov3+1;

endif;

bb2=inv(Seinv1'*Seinv1)*Seinv1't1;

s2wls2=(t1-Seinv1*bb2)'*(t1-Seinv1*bb2)/(rr-1);

WLSVAR2=s2wls2*inv(Seinv1'*Seinv1);

I2=(s2wls2-1)/s2wls2;

if I2<0;I2=0;endif;sum8=sum8+I2;

/*Calculates Hunter&Schmidt--see H&S(1990) &Field(2001)*/

HS=(n'*r)/(z1'*n);/*Calculates Hunter&Schmidt MA*/

PP=HS;

VarHS=(n'*(r-HS*z1)^2)/(z1'n);

SEHS=sqrt((VarHS)/rr);/*Calculates Hunter&Schmidt's MA's SE*/

PPse=SEHS;

UL2=PP+1.96*PPse;

LL2=PP-1.96*PPse;

if LL2<=sqrt(1/5) and UL2>=sqrt(1/5);

cov2=cov2+1;

endif;

sum2=sum2+PP;MSE2=MSE2+(PP-sqrt(1/5))^2;

/* The below program calculates REz */

Wave4=sumW4/rr;/*see Eq (5.3) in Sutton et al (2000) */

S2w4=(t4'*z1-rr*Wave4^2)/(rr-1);/*Eq (5.4)*/

U=(rr-1)*(Wave4-S2w4/(rr*Wave4));/*Eq (5.5)*/

Q=(W4i'Zf^2)-(Zf'*W4i)^2/sumW4;/*Eq (3.2)*/

BSvar=(Q-rr+1)/U;/*Eq (5.6)*/

if Q<=rr-1;

BSvar=0;

endif;

iBS=1; weight={};

do while (iBS<=rr);

weighti=1/(BSvar+Ssq4[iBS]);/*Eq(5.7)*/

weight=weight|weighti;

iBS=iBS+1;

endo;

sumWeight1=weight'*z1;

REWA1=(Zf'*weight)/sumWeight1; /*Eq(5.8)*/

VARRE=1/sumWeight1;

ulr=REWA1+1.96*sqrt(VARRE);ulr=((exp(2*ulr)-1)/(exp(2*ulr)+1));

llr=REWA1-1.96*sqrt(VARRE);llr=((exp(2*llr)-1)/(exp(2*llr)+1));

if llr<=sqrt(1/5) and ulr>=sqrt(1/5);

cov1=cov1+1;

endif;

REzCorr=(exp(2*REWA1)-1)/(exp(2*REWA1)+1);

sum5=sum5+REzCorr;MSE1=MSE1+(REzCorr-sqrt(1/5))^2;

/*Calculates PET-PEESE using Fisher's z truncated at zero*/

zz=Seinv4~Sez;bb5=inv(zz'*zz)*zz't4;/*PEESE*/

PEESEzS2=(t4-zz*bb5)'*(t4-zz*bb5)/(rr-2);

PEESEzVAR=PEESEzS2*inv(zz'*zz);

zz=z1~Seinv4;bb6=inv(zz'*zz)*zz't4;/*PET*/

/*PET variance*/

PETzS2=(t4-zz*bb6)'*(t4-zz*bb6)/(rr-2);

PETVARz=PETzS2*inv(zz'zz);PETzSE=sqrt(PETVARz[2,2]);

teff=bb6[2]/PETzSE;PPz=bb6[2];PPsez=PETzSE;

if teff>=1.299;

PPz=bb5[1];PPsez=sqrt(PEESEzVAR[1,1]);

endif;

if PPz<0;PPz=0;endif;PPcorr=(exp(2*PPz)-1)/(exp(2*PPz)+1);

UL3=PPz+2.008*PPsez;UL3=((exp(2*UL3)-1)/(exp(2*UL3)+1));

LL3=PPz-2.008*PPsez;LL3=((exp(2*LL3)-1)/(exp(2*LL3)+1));

if LL3<=sqrt(1/5) and UL3>=sqrt(1/5);

cov4=cov4+1;

endif;

sum4=sum4+PPcorr;MSE4=MSE4+(PPcorr-sqrt(1/5))^2;

k=k+1;

endo;

UWLS3Bias=(sum1/rep)-sqrt(1/5);UWLS3cov=cov3/rep;

REzBias=(sum5/rep)-sqrt(1/5);REzcov=cov1/rep;Isq=sum8/rep;

PPBias=(sum2/rep)-sqrt(1/5);PPcov=cov2/rep;

PPzBias=(sum4/rep)-sqrt(1/5);PPzcov=cov4/rep;

UWLS3RMSE=(MSE3/rep)^.5;PPzRMSE=(MSE4/rep)^.5;

REzRMSE=(MSE1/rep)^.5;PPRMSE=(MSE2/rep)^.5;

AveBias=(sum3/rep)-sqrt(1/5);

screen on;

"UWLS3,REz,HS,PPz:NoHet,CorrHet& PB50;k=50;n={30,40,50,75,100,100,125,160,200,400};True r=sqrt{1/5;1/17;1/82}";

"I-sq, Average bias, UWLS3, z, Hunter&Schmidt, PPz, Coverages, MSEs";

Isq;;AveBias;;UWLS3Bias;;REzBias;;PPBias;;PPzBias;;UWLS3cov;;REzcov;;PPcov;;PPzcov;;UWLS3RMSE;;REzRMSE;;PPRMSE;;PPzRMSE;

screen off;

sum1=0;sum2=0;sum3=0;sum10=0;sum11=0;

MSE1=0;MSE2=0;MSE3=0;MSE4=0;

sum4=0;sum5=0;sum6=0;sum7=0;sum9=0;sum8=0;

cov1=0;cov4=0;cov2=0;cov3=0;

ID={30,40,50,75,100,100,125,160,200,400};/* sample size of the original studies*/

het={.45,.45,.3,.3,.3,.3,.3,.3,.075,.075};

critt={2.048,2.024,2.011,1.993,1.984,1.984,1.98,1.978,1.975,1.97};

k=1;

do while (k<=rep);/* the number of replications*/

Seinv1={};Seinv2={};Seinv3={};Seinv4={};

t1={}; t2={};r={};t3={};

W1i={};Zf={};Se={};t4={};

Ssq1={};Ssq3={};Sez={};

W2i={};W3i={};W4i={};

Ssq2={}; Ssq4={};

/* space holder 1*/

/* space holder 2*/

/* space holder 3*/

r2=5;/* the number of research studies is the sum times 10*/

j=1;

do while (j<=r2);

i=1;

do while (i<=10);

X2=rndn(ID[i],1)/4;

/* space holder 4*/

/* space holder 5*/

/* space holder 6*/

/*Bivariate Correlation using regressions' t-values */

X1=ones(ID[i],1);

Y=X1+X2+rndn(ID[i],1);

X=X2;

_olsres=1;

{ nam,m,b,stb,vc,std,sig,cx,rsq,resid,dbw } = ols(0,Y,X);

ti=b[2]/std[2];/*corr is conventional corr, corr for UWLS3*/

corr=ti/(ti^2+ID[i]-2)^.5;cor=ti/(ti^2+ID[i]+1)^.5;

b1=0;/*adds random Het in terms of Cohen's d*/

d1=b1+2*corr/(1-corr^2)^.5;corr=d1/(d1^2+4)^.5;

d2=b1+2*cor/(1-cor^2)^.5;cor=d2/(d2^2+4)^.5;

Zfi=.5*ln((1+corr)/(1-corr));/* Fisher's z*/

r=r|corr;Zf=Zf|Zfi;

/* SEtest is the SE for testing whether corr=0*/

SEtest=sqrt((1-corr^2)/(ID[i]-2));

/* SEma is the conventional meta-analysis SE for corr's*/

SEma=(1-corr^2)/sqrt((ID[i]-1));

/* SE3 is the SE for UWLS+3*/

SE3=sqrt((1-cor^2)/(ID[i]+1));

Se=Se|SEtest;

/* SE4 is the SE for Fisher's z*/

SE4=1/sqrt(ID[i]-3);Sez=Sez|SE4;

t1i=corr/SEma;Seinv1i=1/SEma;

t2i=corr/SEtest;Seinv2i=1/SEtest;

Seinv1=Seinv1|Seinv1i;t1=t1|t1i;

Seinv2=Seinv2|Seinv2i;t2=t2|t2i;

t3i=cor/SE3;Seinv3i=1/SE3;

t4i=Zfi/SE4;Seinv4i=1/SE4;

Seinv3=Seinv3|Seinv3i;t3=t3|t3i;

Seinv4=Seinv4|Seinv4i;t4=t4|t4i;

W4ii=Seinv4i^2;

W4i=W4i|W4ii;

Ssq4=Ssq4|SE4^2;

i=i+1;

endo;

j=j+1;

endo;

rr=10*r2;

z1=ones(rr,1);n=W4i+(3*z1);

ave=r'*z1/rr; sum3=sum3+ave;

sumW4=W4i'*z1;

/* The below program calculates UWLS+3 & UWLS1 for I-sq */

bb1=inv(Seinv3'*Seinv3)*Seinv3't3;sum1=sum1+bb1;

s2wls=(t3-Seinv3*bb1)'*(t3-Seinv3*bb1)/(rr-1);

WLSVAR1=s2wls*inv(Seinv3'*Seinv3);MSE3=MSE3+(bb1[1]-sqrt(1/17))^2;

UL1=bb1[1]+2.01*sqrt(WLSVAR1);

LL1=bb1[1]-2.01*sqrt(WLSVAR1);

if LL1<=sqrt(1/17) and UL1>=sqrt(1/17);

cov3=cov3+1;

endif;

bb2=inv(Seinv1'*Seinv1)*Seinv1't1;

s2wls2=(t1-Seinv1*bb2)'*(t1-Seinv1*bb2)/(rr-1);

WLSVAR2=s2wls2*inv(Seinv1'*Seinv1);

I2=(s2wls2-1)/s2wls2;

if I2<0;I2=0;endif;sum8=sum8+I2;

/*Calculates Hunter&Schmidt--see H&S(1990) &Field(2001)*/

HS=(n'*r)/(z1'*n);/*Calculates Hunter&Schmidt MA*/

PP=HS;

VarHS=(n'*(r-HS*z1)^2)/(z1'n);

SEHS=sqrt((VarHS)/rr);/*Calculates Hunter&Schmidt's MA's SE*/

PPse=SEHS;

UL2=PP+1.96*PPse;

LL2=PP-1.96*PPse;

if LL2<=sqrt(1/17) and UL2>=sqrt(1/17);

cov2=cov2+1;

endif;

sum2=sum2+PP;MSE2=MSE2+(PP-sqrt(1/17))^2;

Wave4=sumW4/rr;/*see Eq (5.3) in Sutton et al (2000) */

S2w4=(t4'*z1-rr*Wave4^2)/(rr-1);/*Eq (5.4)*/

U=(rr-1)*(Wave4-S2w4/(rr*Wave4));/*Eq (5.5)*/

Q=(W4i'Zf^2)-(Zf'*W4i)^2/sumW4;/*Eq (3.2)*/

BSvar=(Q-rr+1)/U;/*Eq (5.6)*/

if Q<=rr-1;

BSvar=0;

endif;

iBS=1; weight={};

do while (iBS<=rr);

weighti=1/(BSvar+Ssq4[iBS]);/*Eq(5.7)*/

weight=weight|weighti;

iBS=iBS+1;

endo;

sumWeight1=weight'*z1;

REWA1=(Zf'*weight)/sumWeight1; /*Eq(5.8)*/

VARRE=1/sumWeight1;

ulr=REWA1+1.96*sqrt(VARRE);ulr=((exp(2*ulr)-1)/(exp(2*ulr)+1));

llr=REWA1-1.96*sqrt(VARRE);llr=((exp(2*llr)-1)/(exp(2*llr)+1));

if llr<=sqrt(1/17) and ulr>=sqrt(1/17);

cov1=cov1+1;

endif;

REzCorr=(exp(2*REWA1)-1)/(exp(2*REWA1)+1);

sum5=sum5+REzCorr;MSE1=MSE1+(REzCorr-sqrt(1/17))^2;

/*Calculates PET-PEESE using Fisher's z truncated at zero*/

zz=Seinv4~Sez;bb5=inv(zz'*zz)*zz't4;/*PEESE*/

PEESEzS2=(t4-zz*bb5)'*(t4-zz*bb5)/(rr-2);

PEESEzVAR=PEESEzS2*inv(zz'*zz);

zz=z1~Seinv4;bb6=inv(zz'*zz)*zz't4;/*PET*/

/*PET variance*/

PETzS2=(t4-zz*bb6)'*(t4-zz*bb6)/(rr-2);

PETVARz=PETzS2*inv(zz'zz);PETzSE=sqrt(PETVARz[2,2]);

teff=bb6[2]/PETzSE;PPz=bb6[2];PPsez=PETzSE;

if teff>=1.299;

PPz=bb5[1];PPsez=sqrt(PEESEzVAR[1,1]);

endif;

if PPz<0;PPz=0;endif;PPcorr=(exp(2*PPz)-1)/(exp(2*PPz)+1);

UL3=PPz+2.008*PPsez;UL3=((exp(2*UL3)-1)/(exp(2*UL3)+1));

LL3=PPz-2.008*PPsez;LL3=((exp(2*LL3)-1)/(exp(2*LL3)+1));

if LL3<=sqrt(1/17) and UL3>=sqrt(1/17);

cov4=cov4+1;

endif;

sum4=sum4+PPcorr;MSE4=MSE4+(PPcorr-sqrt(1/17))^2;

k=k+1;

endo;

UWLS3Bias=(sum1/rep)-sqrt(1/17);UWLS3cov=cov3/rep;

REzBias=(sum5/rep)-sqrt(1/17);REzcov=cov1/rep;Isq=sum8/rep;

PPBias=(sum2/rep)-sqrt(1/17);PPcov=cov2/rep;

PPzBias=(sum4/rep)-sqrt(1/17);PPzcov=cov4/rep;

UWLS3RMSE=(MSE3/rep)^.5;PPzRMSE=(MSE4/rep)^.5;

REzRMSE=(MSE1/rep)^.5;PPRMSE=(MSE2/rep)^.5;

AveBias=(sum3/rep)-sqrt(1/17);

screen on;

Isq;;AveBias;;UWLS3Bias;;REzBias;;PPBias;;PPzBias;;UWLS3cov;;REzcov;;PPcov;;PPzcov;;UWLS3RMSE;;REzRMSE;;PPRMSE;;PPzRMSE;

screen off;

sum1=0;sum2=0;sum3=0;sum10=0;sum11=0;

MSE1=0;MSE2=0;MSE3=0;MSE4=0;

sum4=0;sum5=0;sum6=0;sum7=0;sum9=0;sum8=0;

cov1=0;cov4=0;cov2=0;cov3=0;

ID={30,40,50,75,100,100,125,160,200,400};/* sample size of the original studies*/

het={.45,.45,.3,.3,.3,.3,.3,.3,.075,.075};

critt={2.048,2.024,2.011,1.993,1.984,1.984,1.98,1.978,1.975,1.97};

k=1;

do while (k<=rep);/* the number of replications*/

Seinv1={};Seinv2={};Seinv3={};Seinv4={};

t1={}; t2={};r={};t3={};

W1i={};Zf={};Se={};t4={};

Ssq1={};Ssq3={};Sez={};

W2i={};W3i={};W4i={};

Ssq2={}; Ssq4={};

/* space holder 1*/

/* space holder 2*/

/* space holder 3*/

r2=5;/* the number of research studies is the sum times 10*/

j=1;

do while (j<=r2);

i=1;

do while (i<=10);

X2=rndn(ID[i],1)/9;

/* space holder 4*/

/* space holder 5*/

/* space holder 6*/

/*Bivariate Correlation using regressions' t-values */

X1=ones(ID[i],1);

Y=X1+X2+rndn(ID[i],1);

X=X2;

_olsres=1;

{ nam,m,b,stb,vc,std,sig,cx,rsq,resid,dbw } = ols(0,Y,X);

ti=b[2]/std[2];/*corr is conventional corr, corr for UWLS3*/

corr=ti/(ti^2+ID[i]-2)^.5;cor=ti/(ti^2+ID[i]+1)^.5;

b1=0;/*adds random Het in terms of Cohen's d*/

d1=b1+2*corr/(1-corr^2)^.5;corr=d1/(d1^2+4)^.5;

d2=b1+2*cor/(1-cor^2)^.5;cor=d2/(d2^2+4)^.5;

Zfi=.5*ln((1+corr)/(1-corr));/* Fisher's z*/

r=r|corr;Zf=Zf|Zfi;

/* SEtest is the SE for testing whether corr=0*/

SEtest=sqrt((1-corr^2)/(ID[i]-2));

/* SEma is the conventional meta-analysis SE for corr's*/

SEma=(1-corr^2)/sqrt((ID[i]-1));

/* SE3 is the SE for UWLS+3*/

SE3=sqrt((1-cor^2)/(ID[i]+1));

Se=Se|SEtest;

/* SE4 is the SE for Fisher's z*/

SE4=1/sqrt(ID[i]-3);Sez=Sez|SE4;

t1i=corr/SEma;Seinv1i=1/SEma;

t2i=corr/SEtest;Seinv2i=1/SEtest;

Seinv1=Seinv1|Seinv1i;t1=t1|t1i;

Seinv2=Seinv2|Seinv2i;t2=t2|t2i;

t3i=cor/SE3;Seinv3i=1/SE3;

t4i=Zfi/SE4;Seinv4i=1/SE4;

Seinv3=Seinv3|Seinv3i;t3=t3|t3i;

Seinv4=Seinv4|Seinv4i;t4=t4|t4i;

W4ii=Seinv4i^2;

W4i=W4i|W4ii;

Ssq4=Ssq4|SE4^2;

i=i+1;

endo;

j=j+1;

endo;

rr=10*r2;

z1=ones(rr,1);n=W4i+(3*z1);

ave=r'*z1/rr; sum3=sum3+ave;

sumW4=W4i'*z1;

/* The below program calculates UWLS+3 & UWLS1 for I-sq */

bb1=inv(Seinv3'*Seinv3)*Seinv3't3;sum1=sum1+bb1;

s2wls=(t3-Seinv3*bb1)'*(t3-Seinv3*bb1)/(rr-1);

WLSVAR1=s2wls*inv(Seinv3'*Seinv3);MSE3=MSE3+(bb1[1]-sqrt(1/82))^2;

UL1=bb1[1]+2.01*sqrt(WLSVAR1);

LL1=bb1[1]-2.01*sqrt(WLSVAR1);

if LL1<=sqrt(1/82) and UL1>=sqrt(1/82);

cov3=cov3+1;

endif;

bb2=inv(Seinv1'*Seinv1)*Seinv1't1;

s2wls2=(t1-Seinv1*bb2)'*(t1-Seinv1*bb2)/(rr-1);

WLSVAR2=s2wls2*inv(Seinv1'*Seinv1);

I2=(s2wls2-1)/s2wls2;

if I2<0;I2=0;endif;sum8=sum8+I2;

/*Calculates Hunter&Schmidt--see H&S(1990) &Field(2001)*/

HS=(n'*r)/(z1'*n);/*Calculates Hunter&Schmidt MA*/

PP=HS;

VarHS=(n'*(r-HS*z1)^2)/(z1'n);

SEHS=sqrt((VarHS)/rr);/*Calculates Hunter&Schmidt's MA's SE*/

PPse=SEHS;

UL2=PP+1.96*PPse;

LL2=PP-1.96*PPse;

if LL2<=sqrt(1/82) and UL2>=sqrt(1/82);

cov2=cov2+1;

endif;

sum2=sum2+PP;MSE2=MSE2+(PP-sqrt(1/82))^2;

Wave4=sumW4/rr;/*see Eq (5.3) in Sutton et al (2000) */

S2w4=(t4'*z1-rr*Wave4^2)/(rr-1);/*Eq (5.4)*/

U=(rr-1)*(Wave4-S2w4/(rr*Wave4));/*Eq (5.5)*/

Q=(W4i'Zf^2)-(Zf'*W4i)^2/sumW4;/*Eq (3.2)*/

BSvar=(Q-rr+1)/U;/*Eq (5.6)*/

if Q<=rr-1;

BSvar=0;

endif;

iBS=1; weight={};

do while (iBS<=rr);

weighti=1/(BSvar+Ssq4[iBS]);/*Eq(5.7)*/

weight=weight|weighti;

iBS=iBS+1;

endo;

sumWeight1=weight'*z1;

REWA1=(Zf'*weight)/sumWeight1; /*Eq(5.8)*/

VARRE=1/sumWeight1;

ulr=REWA1+1.96*sqrt(VARRE);ulr=((exp(2*ulr)-1)/(exp(2*ulr)+1));

llr=REWA1-1.96*sqrt(VARRE);llr=((exp(2*llr)-1)/(exp(2*llr)+1));

if llr<=sqrt(1/82) and ulr>=sqrt(1/82);

cov1=cov1+1;

endif;

REzCorr=(exp(2*REWA1)-1)/(exp(2*REWA1)+1);

sum5=sum5+REzCorr;MSE1=MSE1+(REzCorr-sqrt(1/82))^2;

/*Calculates PET-PEESE using Fisher's z truncated at zero*/

zz=Seinv4~Sez;bb5=inv(zz'*zz)*zz't4;/*PEESE*/

PEESEzS2=(t4-zz*bb5)'*(t4-zz*bb5)/(rr-2);

PEESEzVAR=PEESEzS2*inv(zz'*zz);

zz=z1~Seinv4;bb6=inv(zz'*zz)*zz't4;/*PET*/

/*PET variance*/

PETzS2=(t4-zz*bb6)'*(t4-zz*bb6)/(rr-2);

PETVARz=PETzS2*inv(zz'zz);PETzSE=sqrt(PETVARz[2,2]);

teff=bb6[2]/PETzSE;PPz=bb6[2];PPsez=PETzSE;

if teff>=1.299;

PPz=bb5[1];PPsez=sqrt(PEESEzVAR[1,1]);

endif;

if PPz<0;PPz=0;endif;PPcorr=(exp(2*PPz)-1)/(exp(2*PPz)+1);

UL3=PPz+2.008*PPsez;UL3=((exp(2*UL3)-1)/(exp(2*UL3)+1));

LL3=PPz-2.008*PPsez;LL3=((exp(2*LL3)-1)/(exp(2*LL3)+1));

if LL3<=sqrt(1/82) and UL3>=sqrt(1/82);

cov4=cov4+1;

endif;

sum4=sum4+PPcorr;MSE4=MSE4+(PPcorr-sqrt(1/82))^2;

k=k+1;

endo;

UWLS3Bias=(sum1/rep)-sqrt(1/82);UWLS3cov=cov3/rep;

REzBias=(sum5/rep)-sqrt(1/82);REzcov=cov1/rep;Isq=sum8/rep;

PPBias=(sum2/rep)-sqrt(1/82);PPcov=cov2/rep;

PPzBias=(sum4/rep)-sqrt(1/82);PPzcov=cov4/rep;

UWLS3RMSE=(MSE3/rep)^.5;PPzRMSE=(MSE4/rep)^.5;

REzRMSE=(MSE1/rep)^.5;PPRMSE=(MSE2/rep)^.5;

AveBias=(sum3/rep)-sqrt(1/82);

screen on;

Isq;;AveBias;;UWLS3Bias;;REzBias;;PPBias;;PPzBias;;UWLS3cov;;REzcov;;PPcov;;PPzcov;;UWLS3RMSE;;REzRMSE;;PPRMSE;;PPzRMSE;

screen off;

sum1=0;sum2=0;sum3=0;sum10=0;sum11=0;

MSE1=0;MSE2=0;MSE3=0;MSE4=0;

sum4=0;sum5=0;sum6=0;sum7=0;sum9=0;sum8=0;

cov1=0;cov4=0;cov2=0;cov3=0;

ID={30,40,50,75,100,100,125,160,200,400};/* sample size of the original studies*/

het={.45,.45,.3,.3,.3,.3,.3,.3,.075,.075};

critt={2.048,2.024,2.011,1.993,1.984,1.984,1.98,1.978,1.975,1.97};

k=1;

do while (k<=rep);/* the number of replications*/

Seinv1={};Seinv2={};Seinv3={};Seinv4={};

t1={}; t2={};r={};t3={};

W1i={};Zf={};Se={};t4={};

Ssq1={};Ssq3={};Sez={};

W2i={};W3i={};W4i={};

Ssq2={}; Ssq4={};

/* space holder 1*/

/* space holder 2*/

/* space holder 3*/

r2=5;/* the number of research studies is the sum times 10*/

j=1;

do while (j<=r2);

i=1;

do while (i<=10);

X2=rndn(ID[i],1)/2;

/* space holder 4*/

/* space holder 5*/

/* space holder 6*/

/*Bivariate Correlation using regressions' t-values */

X1=ones(ID[i],1);

Y=X1+X2+rndn(ID[i],1);

X=X2;

_olsres=1;

{ nam,m,b,stb,vc,std,sig,cx,rsq,resid,dbw } = ols(0,Y,X);

ti=b[2]/std[2];/*corr is conventional corr, corr for UWLS3*/

corr=ti/(ti^2+ID[i]-2)^.5;cor=ti/(ti^2+ID[i]+1)^.5;

b1=het[i]*rndn(1,1);/*adds random Het in terms of Cohen's d*/

d1=b1+2*corr/(1-corr^2)^.5;corr=d1/(d1^2+4)^.5;

d2=b1+2*cor/(1-cor^2)^.5;cor=d2/(d2^2+4)^.5;

Zfi=.5*ln((1+corr)/(1-corr));/* Fisher's z*/

r=r|corr;Zf=Zf|Zfi;

/* SEtest is the SE for testing whether corr=0*/

SEtest=sqrt((1-corr^2)/(ID[i]-2));

/* SEma is the conventional meta-analysis SE for corr's*/

SEma=(1-corr^2)/sqrt((ID[i]-1));

/* SE3 is the SE for UWLS+3*/

SE3=sqrt((1-cor^2)/(ID[i]+1));

Se=Se|SEtest;

/* SE4 is the SE for Fisher's z*/

SE4=1/sqrt(ID[i]-3);Sez=Sez|SE4;

t1i=corr/SEma;Seinv1i=1/SEma;

t2i=corr/SEtest;Seinv2i=1/SEtest;

Seinv1=Seinv1|Seinv1i;t1=t1|t1i;

Seinv2=Seinv2|Seinv2i;t2=t2|t2i;

t3i=cor/SE3;Seinv3i=1/SE3;

t4i=Zfi/SE4;Seinv4i=1/SE4;

Seinv3=Seinv3|Seinv3i;t3=t3|t3i;

Seinv4=Seinv4|Seinv4i;t4=t4|t4i;

W4ii=Seinv4i^2;

W4i=W4i|W4ii;

Ssq4=Ssq4|SE4^2;

i=i+1;

endo;

j=j+1;

endo;

rr=10*r2;

z1=ones(rr,1);n=W4i+(3*z1);

ave=r'*z1/rr; sum3=sum3+ave;

sumW4=W4i'*z1;

/* The below program calculates UWLS+3 & UWLS1 for I-sq */

bb1=inv(Seinv3'*Seinv3)*Seinv3't3;sum1=sum1+bb1;

s2wls=(t3-Seinv3*bb1)'*(t3-Seinv3*bb1)/(rr-1);

WLSVAR1=s2wls*inv(Seinv3'*Seinv3);MSE3=MSE3+(bb1[1]-sqrt(1/5))^2;

UL1=bb1[1]+2.01*sqrt(WLSVAR1);

LL1=bb1[1]-2.01*sqrt(WLSVAR1);

if LL1<=sqrt(1/5) and UL1>=sqrt(1/5);

cov3=cov3+1;

endif;

bb2=inv(Seinv1'*Seinv1)*Seinv1't1;

s2wls2=(t1-Seinv1*bb2)'*(t1-Seinv1*bb2)/(rr-1);

WLSVAR2=s2wls2*inv(Seinv1'*Seinv1);

I2=(s2wls2-1)/s2wls2;

if I2<0;I2=0;endif;sum8=sum8+I2;

/*Calculates Hunter&Schmidt--see H&S(1990) &Field(2001)*/

HS=(n'*r)/(z1'*n);/*Calculates Hunter&Schmidt MA*/

PP=HS;

VarHS=(n'*(r-HS*z1)^2)/(z1'n);

SEHS=sqrt((VarHS)/rr);/*Calculates Hunter&Schmidt's MA's SE*/

PPse=SEHS;

UL2=PP+1.96*PPse;

LL2=PP-1.96*PPse;

if LL2<=sqrt(1/5) and UL2>=sqrt(1/5);

cov2=cov2+1;

endif;

sum2=sum2+PP;MSE2=MSE2+(PP-sqrt(1/5))^2;

Wave4=sumW4/rr;/*see Eq (5.3) in Sutton et al (2000) */

S2w4=(t4'*z1-rr*Wave4^2)/(rr-1);/*Eq (5.4)*/

U=(rr-1)*(Wave4-S2w4/(rr*Wave4));/*Eq (5.5)*/

Q=(W4i'Zf^2)-(Zf'*W4i)^2/sumW4;/*Eq (3.2)*/

BSvar=(Q-rr+1)/U;/*Eq (5.6)*/

if Q<=rr-1;

BSvar=0;

endif;

iBS=1; weight={};

do while (iBS<=rr);

weighti=1/(BSvar+Ssq4[iBS]);/*Eq(5.7)*/

weight=weight|weighti;

iBS=iBS+1;

endo;

sumWeight1=weight'*z1;

REWA1=(Zf'*weight)/sumWeight1; /*Eq(5.8)*/

VARRE=1/sumWeight1;

ulr=REWA1+1.96*sqrt(VARRE);ulr=((exp(2*ulr)-1)/(exp(2*ulr)+1));

llr=REWA1-1.96*sqrt(VARRE);llr=((exp(2*llr)-1)/(exp(2*llr)+1));

if llr<=sqrt(1/5) and ulr>=sqrt(1/5);

cov1=cov1+1;

endif;

REzCorr=(exp(2*REWA1)-1)/(exp(2*REWA1)+1);

sum5=sum5+REzCorr;MSE1=MSE1+(REzCorr-sqrt(1/5))^2;

/*Calculates PET-PEESE using Fisher's z truncated at zero*/

zz=Seinv4~Sez;bb5=inv(zz'*zz)*zz't4;/*PEESE*/

PEESEzS2=(t4-zz*bb5)'*(t4-zz*bb5)/(rr-2);

PEESEzVAR=PEESEzS2*inv(zz'*zz);

zz=z1~Seinv4;bb6=inv(zz'*zz)*zz't4;/*PET*/

/*PET variance*/

PETzS2=(t4-zz*bb6)'*(t4-zz*bb6)/(rr-2);

PETVARz=PETzS2*inv(zz'zz);PETzSE=sqrt(PETVARz[2,2]);

teff=bb6[2]/PETzSE;PPz=bb6[2];PPsez=PETzSE;

if teff>=1.299;

PPz=bb5[1];PPsez=sqrt(PEESEzVAR[1,1]);

endif;

if PPz<0;PPz=0;endif;PPcorr=(exp(2*PPz)-1)/(exp(2*PPz)+1);

UL3=PPz+2.008*PPsez;UL3=((exp(2*UL3)-1)/(exp(2*UL3)+1));

LL3=PPz-2.008*PPsez;LL3=((exp(2*LL3)-1)/(exp(2*LL3)+1));

if LL3<=sqrt(1/5) and UL3>=sqrt(1/5);

cov4=cov4+1;

endif;

sum4=sum4+PPcorr;MSE4=MSE4+(PPcorr-sqrt(1/5))^2;

k=k+1;

endo;

UWLS3Bias=(sum1/rep)-sqrt(1/5);UWLS3cov=cov3/rep;

REzBias=(sum5/rep)-sqrt(1/5);REzcov=cov1/rep;Isq=sum8/rep;

PPBias=(sum2/rep)-sqrt(1/5);PPcov=cov2/rep;

PPzBias=(sum4/rep)-sqrt(1/5);PPzcov=cov4/rep;

UWLS3RMSE=(MSE3/rep)^.5;PPzRMSE=(MSE4/rep)^.5;

REzRMSE=(MSE1/rep)^.5;PPRMSE=(MSE2/rep)^.5;

AveBias=(sum3/rep)-sqrt(1/5);

screen on;

Isq;;AveBias;;UWLS3Bias;;REzBias;;PPBias;;PPzBias;;UWLS3cov;;REzcov;;PPcov;;PPzcov;;UWLS3RMSE;;REzRMSE;;PPRMSE;;PPzRMSE;

screen off;

sum1=0;sum2=0;sum3=0;sum10=0;sum11=0;

MSE1=0;MSE2=0;MSE3=0;MSE4=0;

sum4=0;sum5=0;sum6=0;sum7=0;sum9=0;sum8=0;

cov1=0;cov4=0;cov2=0;cov3=0;

ID={30,40,50,75,100,100,125,160,200,400};/* sample size of the original studies*/

het={.45,.45,.3,.3,.3,.3,.3,.3,.075,.075};

critt={2.048,2.024,2.011,1.993,1.984,1.984,1.98,1.978,1.975,1.97};

k=1;

do while (k<=rep);/* the number of replications*/

Seinv1={};Seinv2={};Seinv3={};Seinv4={};

t1={}; t2={};r={};t3={};

W1i={};Zf={};Se={};t4={};

Ssq1={};Ssq3={};Sez={};

W2i={};W3i={};W4i={};

Ssq2={}; Ssq4={};

/* space holder 1*/

/* space holder 2*/

/* space holder 3*/

r2=5;/* the number of research studies is the sum times 10*/

j=1;

do while (j<=r2);

i=1;

do while (i<=10);

X2=rndn(ID[i],1)/4;

/* space holder 4*/

/* space holder 5*/

/* space holder 6*/

/*Bivariate Correlation using regressions' t-values */

X1=ones(ID[i],1);

Y=X1+X2+rndn(ID[i],1);

X=X2;

_olsres=1;

{ nam,m,b,stb,vc,std,sig,cx,rsq,resid,dbw } = ols(0,Y,X);

ti=b[2]/std[2];/*corr is conventional corr, corr for UWLS3*/

corr=ti/(ti^2+ID[i]-2)^.5;cor=ti/(ti^2+ID[i]+1)^.5;

b1=het[i]*rndn(1,1);/*adds random Het in terms of Cohen's d*/

d1=b1+2*corr/(1-corr^2)^.5;corr=d1/(d1^2+4)^.5;

d2=b1+2*cor/(1-cor^2)^.5;cor=d2/(d2^2+4)^.5;

Zfi=.5*ln((1+corr)/(1-corr));/* Fisher's z*/

r=r|corr;Zf=Zf|Zfi;

/* SEtest is the SE for testing whether corr=0*/

SEtest=sqrt((1-corr^2)/(ID[i]-2));

/* SEma is the conventional meta-analysis SE for corr's*/

SEma=(1-corr^2)/sqrt((ID[i]-1));

/* SE3 is the SE for UWLS+3*/

SE3=sqrt((1-cor^2)/(ID[i]+1));

Se=Se|SEtest;

/* SE4 is the SE for Fisher's z*/

SE4=1/sqrt(ID[i]-3);Sez=Sez|SE4;

t1i=corr/SEma;Seinv1i=1/SEma;

t2i=corr/SEtest;Seinv2i=1/SEtest;

Seinv1=Seinv1|Seinv1i;t1=t1|t1i;

Seinv2=Seinv2|Seinv2i;t2=t2|t2i;

t3i=cor/SE3;Seinv3i=1/SE3;

t4i=Zfi/SE4;Seinv4i=1/SE4;

Seinv3=Seinv3|Seinv3i;t3=t3|t3i;

Seinv4=Seinv4|Seinv4i;t4=t4|t4i;

W4ii=Seinv4i^2;

W4i=W4i|W4ii;

Ssq4=Ssq4|SE4^2;

i=i+1;

endo;

j=j+1;

endo;

rr=10*r2;

z1=ones(rr,1);n=W4i+(3*z1);

ave=r'*z1/rr; sum3=sum3+ave;

sumW4=W4i'*z1;

/* The below program calculates UWLS+3 & UWLS1 for I-sq */

bb1=inv(Seinv3'*Seinv3)*Seinv3't3;sum1=sum1+bb1;

s2wls=(t3-Seinv3*bb1)'*(t3-Seinv3*bb1)/(rr-1);

WLSVAR1=s2wls*inv(Seinv3'*Seinv3);MSE3=MSE3+(bb1[1]-sqrt(1/17))^2;

UL1=bb1[1]+2.01*sqrt(WLSVAR1);

LL1=bb1[1]-2.01*sqrt(WLSVAR1);

if LL1<=sqrt(1/17) and UL1>=sqrt(1/17);

cov3=cov3+1;

endif;

bb2=inv(Seinv1'*Seinv1)*Seinv1't1;

s2wls2=(t1-Seinv1*bb2)'*(t1-Seinv1*bb2)/(rr-1);

WLSVAR2=s2wls2*inv(Seinv1'*Seinv1);

I2=(s2wls2-1)/s2wls2;

if I2<0;I2=0;endif;sum8=sum8+I2;

/*Calculates Hunter&Schmidt--see H&S(1990) &Field(2001)*/

HS=(n'*r)/(z1'*n);/*Calculates Hunter&Schmidt MA*/

PP=HS;

VarHS=(n'*(r-HS*z1)^2)/(z1'n);

SEHS=sqrt((VarHS)/rr);/*Calculates Hunter&Schmidt's MA's SE*/

PPse=SEHS;

UL2=PP+1.96*PPse;

LL2=PP-1.96*PPse;

if LL2<=sqrt(1/17) and UL2>=sqrt(1/17);

cov2=cov2+1;

endif;

sum2=sum2+PP;MSE2=MSE2+(PP-sqrt(1/17))^2;

Wave4=sumW4/rr;/*see Eq (5.3) in Sutton et al (2000) */

S2w4=(t4'*z1-rr*Wave4^2)/(rr-1);/*Eq (5.4)*/

U=(rr-1)*(Wave4-S2w4/(rr*Wave4));/*Eq (5.5)*/

Q=(W4i'Zf^2)-(Zf'*W4i)^2/sumW4;/*Eq (3.2)*/

BSvar=(Q-rr+1)/U;/*Eq (5.6)*/

if Q<=rr-1;

BSvar=0;

endif;

iBS=1; weight={};

do while (iBS<=rr);

weighti=1/(BSvar+Ssq4[iBS]);/*Eq(5.7)*/

weight=weight|weighti;

iBS=iBS+1;

endo;

sumWeight1=weight'*z1;

REWA1=(Zf'*weight)/sumWeight1; /*Eq(5.8)*/

VARRE=1/sumWeight1;

ulr=REWA1+1.96*sqrt(VARRE);ulr=((exp(2*ulr)-1)/(exp(2*ulr)+1));

llr=REWA1-1.96*sqrt(VARRE);llr=((exp(2*llr)-1)/(exp(2*llr)+1));

if llr<=sqrt(1/17) and ulr>=sqrt(1/17);

cov1=cov1+1;

endif;

REzCorr=(exp(2*REWA1)-1)/(exp(2*REWA1)+1);

sum5=sum5+REzCorr;MSE1=MSE1+(REzCorr-sqrt(1/17))^2;

/*Calculates PET-PEESE using Fisher's z truncated at zero*/

zz=Seinv4~Sez;bb5=inv(zz'*zz)*zz't4;/*PEESE*/

PEESEzS2=(t4-zz*bb5)'*(t4-zz*bb5)/(rr-2);

PEESEzVAR=PEESEzS2*inv(zz'*zz);

zz=z1~Seinv4;bb6=inv(zz'*zz)*zz't4;/*PET*/

/*PET variance*/

PETzS2=(t4-zz*bb6)'*(t4-zz*bb6)/(rr-2);

PETVARz=PETzS2*inv(zz'zz);PETzSE=sqrt(PETVARz[2,2]);

teff=bb6[2]/PETzSE;PPz=bb6[2];PPsez=PETzSE;

if teff>=1.299;

PPz=bb5[1];PPsez=sqrt(PEESEzVAR[1,1]);

endif;

if PPz<0;PPz=0;endif;PPcorr=(exp(2*PPz)-1)/(exp(2*PPz)+1);

UL3=PPz+2.008*PPsez;UL3=((exp(2*UL3)-1)/(exp(2*UL3)+1));

LL3=PPz-2.008*PPsez;LL3=((exp(2*LL3)-1)/(exp(2*LL3)+1));

if LL3<=sqrt(1/17) and UL3>=sqrt(1/17);

cov4=cov4+1;

endif;

sum4=sum4+PPcorr;MSE4=MSE4+(PPcorr-sqrt(1/17))^2;

k=k+1;

endo;

UWLS3Bias=(sum1/rep)-sqrt(1/17);UWLS3cov=cov3/rep;

REzBias=(sum5/rep)-sqrt(1/17);REzcov=cov1/rep;Isq=sum8/rep;

PPBias=(sum2/rep)-sqrt(1/17);PPcov=cov2/rep;

PPzBias=(sum4/rep)-sqrt(1/17);PPzcov=cov4/rep;

UWLS3RMSE=(MSE3/rep)^.5;PPzRMSE=(MSE4/rep)^.5;

REzRMSE=(MSE1/rep)^.5;PPRMSE=(MSE2/rep)^.5;

AveBias=(sum3/rep)-sqrt(1/17);

screen on;

Isq;;AveBias;;UWLS3Bias;;REzBias;;PPBias;;PPzBias;;UWLS3cov;;REzcov;;PPcov;;PPzcov;;UWLS3RMSE;;REzRMSE;;PPRMSE;;PPzRMSE;

screen off;

sum1=0;sum2=0;sum3=0;sum10=0;sum11=0;

MSE1=0;MSE2=0;MSE3=0;MSE4=0;

sum4=0;sum5=0;sum6=0;sum7=0;sum9=0;sum8=0;

cov1=0;cov4=0;cov2=0;cov3=0;

ID={30,40,50,75,100,100,125,160,200,400};/* sample size of the original studies*/

het={.45,.45,.3,.3,.3,.3,.3,.3,.075,.075};

critt={2.048,2.024,2.011,1.993,1.984,1.984,1.98,1.978,1.975,1.97};

k=1;

do while (k<=rep);/* the number of replications*/

Seinv1={};Seinv2={};Seinv3={};Seinv4={};

t1={}; t2={};r={};t3={};

W1i={};Zf={};Se={};t4={};

Ssq1={};Ssq3={};Sez={};

W2i={};W3i={};W4i={};

Ssq2={}; Ssq4={};

/* space holder 1*/

/* space holder 2*/

/* space holder 3*/

r2=5;/* the number of research studies is the sum times 10*/

j=1;

do while (j<=r2);

i=1;

do while (i<=10);

X2=rndn(ID[i],1)/9;

/* space holder 4*/

/* space holder 5*/

/* space holder 6*/

/*Bivariate Correlation using regressions' t-values */

X1=ones(ID[i],1);

Y=X1+X2+rndn(ID[i],1);

X=X2;

_olsres=1;

{ nam,m,b,stb,vc,std,sig,cx,rsq,resid,dbw } = ols(0,Y,X);

ti=b[2]/std[2];/*corr is conventional corr, corr for UWLS3*/

corr=ti/(ti^2+ID[i]-2)^.5;cor=ti/(ti^2+ID[i]+1)^.5;

b1=het[i]*rndn(1,1);/*adds random Het in terms of Cohen's d*/

d1=b1+2*corr/(1-corr^2)^.5;corr=d1/(d1^2+4)^.5;

d2=b1+2*cor/(1-cor^2)^.5;cor=d2/(d2^2+4)^.5;

Zfi=.5*ln((1+corr)/(1-corr));/* Fisher's z*/

r=r|corr;Zf=Zf|Zfi;

/* SEtest is the SE for testing whether corr=0*/

SEtest=sqrt((1-corr^2)/(ID[i]-2));

/* SEma is the conventional meta-analysis SE for corr's*/

SEma=(1-corr^2)/sqrt((ID[i]-1));

/* SE3 is the SE for UWLS+3*/

SE3=sqrt((1-cor^2)/(ID[i]+1));

Se=Se|SEtest;

/* SE4 is the SE for Fisher's z*/

SE4=1/sqrt(ID[i]-3);Sez=Sez|SE4;

t1i=corr/SEma;Seinv1i=1/SEma;

t2i=corr/SEtest;Seinv2i=1/SEtest;

Seinv1=Seinv1|Seinv1i;t1=t1|t1i;

Seinv2=Seinv2|Seinv2i;t2=t2|t2i;

t3i=cor/SE3;Seinv3i=1/SE3;

t4i=Zfi/SE4;Seinv4i=1/SE4;

Seinv3=Seinv3|Seinv3i;t3=t3|t3i;

Seinv4=Seinv4|Seinv4i;t4=t4|t4i;

W4ii=Seinv4i^2;

W4i=W4i|W4ii;

Ssq4=Ssq4|SE4^2;

i=i+1;

endo;

j=j+1;

endo;

rr=10*r2;

z1=ones(rr,1);n=W4i+(3*z1);

ave=r'*z1/rr; sum3=sum3+ave;

sumW4=W4i'*z1;

/* The below program calculates UWLS+3 & UWLS1 for I-sq */

bb1=inv(Seinv3'*Seinv3)*Seinv3't3;sum1=sum1+bb1;

s2wls=(t3-Seinv3*bb1)'*(t3-Seinv3*bb1)/(rr-1);

WLSVAR1=s2wls*inv(Seinv3'*Seinv3);MSE3=MSE3+(bb1[1]-sqrt(1/82))^2;

UL1=bb1[1]+2.01*sqrt(WLSVAR1);

LL1=bb1[1]-2.01*sqrt(WLSVAR1);

if LL1<=sqrt(1/82) and UL1>=sqrt(1/82);

cov3=cov3+1;

endif;

bb2=inv(Seinv1'*Seinv1)*Seinv1't1;

s2wls2=(t1-Seinv1*bb2)'*(t1-Seinv1*bb2)/(rr-1);

WLSVAR2=s2wls2*inv(Seinv1'*Seinv1);

I2=(s2wls2-1)/s2wls2;

if I2<0;I2=0;endif;sum8=sum8+I2;

/*Calculates Hunter&Schmidt--see H&S(1990) &Field(2001)*/

HS=(n'*r)/(z1'*n);/*Calculates Hunter&Schmidt MA*/

PP=HS;

VarHS=(n'*(r-HS*z1)^2)/(z1'n);

SEHS=sqrt((VarHS)/rr);/*Calculates Hunter&Schmidt's MA's SE*/

PPse=SEHS;

UL2=PP+1.96*PPse;

LL2=PP-1.96*PPse;

if LL2<=sqrt(1/82) and UL2>=sqrt(1/82);

cov2=cov2+1;

endif;

sum2=sum2+PP;MSE2=MSE2+(PP-sqrt(1/82))^2;

Wave4=sumW4/rr;/*see Eq (5.3) in Sutton et al (2000) */

S2w4=(t4'*z1-rr*Wave4^2)/(rr-1);/*Eq (5.4)*/

U=(rr-1)*(Wave4-S2w4/(rr*Wave4));/*Eq (5.5)*/

Q=(W4i'Zf^2)-(Zf'*W4i)^2/sumW4;/*Eq (3.2)*/

BSvar=(Q-rr+1)/U;/*Eq (5.6)*/

if Q<=rr-1;

BSvar=0;

endif;

iBS=1; weight={};

do while (iBS<=rr);

weighti=1/(BSvar+Ssq4[iBS]);/*Eq(5.7)*/

weight=weight|weighti;

iBS=iBS+1;

endo;

sumWeight1=weight'*z1;

REWA1=(Zf'*weight)/sumWeight1; /*Eq(5.8)*/

VARRE=1/sumWeight1;

ulr=REWA1+1.96*sqrt(VARRE);ulr=((exp(2*ulr)-1)/(exp(2*ulr)+1));

llr=REWA1-1.96*sqrt(VARRE);llr=((exp(2*llr)-1)/(exp(2*llr)+1));

if llr<=sqrt(1/82) and ulr>=sqrt(1/82);

cov1=cov1+1;

endif;

REzCorr=(exp(2*REWA1)-1)/(exp(2*REWA1)+1);

sum5=sum5+REzCorr;MSE1=MSE1+(REzCorr-sqrt(1/82))^2;

/*Calculates PET-PEESE using Fisher's z truncated at zero*/

zz=Seinv4~Sez;bb5=inv(zz'*zz)*zz't4;/*PEESE*/

PEESEzS2=(t4-zz*bb5)'*(t4-zz*bb5)/(rr-2);

PEESEzVAR=PEESEzS2*inv(zz'*zz);

zz=z1~Seinv4;bb6=inv(zz'*zz)*zz't4;/*PET*/

/*PET variance*/

PETzS2=(t4-zz*bb6)'*(t4-zz*bb6)/(rr-2);

PETVARz=PETzS2*inv(zz'zz);PETzSE=sqrt(PETVARz[2,2]);

teff=bb6[2]/PETzSE;PPz=bb6[2];PPsez=PETzSE;

if teff>=1.299;

PPz=bb5[1];PPsez=sqrt(PEESEzVAR[1,1]);

endif;

if PPz<0;PPz=0;endif;PPcorr=(exp(2*PPz)-1)/(exp(2*PPz)+1);

UL3=PPz+2.008*PPsez;UL3=((exp(2*UL3)-1)/(exp(2*UL3)+1));

LL3=PPz-2.008*PPsez;LL3=((exp(2*LL3)-1)/(exp(2*LL3)+1));

if LL3<=sqrt(1/82) and UL3>=sqrt(1/82);

cov4=cov4+1;

endif;

sum4=sum4+PPcorr;MSE4=MSE4+(PPcorr-sqrt(1/82))^2;

k=k+1;

endo;

UWLS3Bias=(sum1/rep)-sqrt(1/82);UWLS3cov=cov3/rep;

REzBias=(sum5/rep)-sqrt(1/82);REzcov=cov1/rep;Isq=sum8/rep;

PPBias=(sum2/rep)-sqrt(1/82);PPcov=cov2/rep;

PPzBias=(sum4/rep)-sqrt(1/82);PPzcov=cov4/rep;

UWLS3RMSE=(MSE3/rep)^.5;PPzRMSE=(MSE4/rep)^.5;

REzRMSE=(MSE1/rep)^.5;PPRMSE=(MSE2/rep)^.5;

AveBias=(sum3/rep)-sqrt(1/82);

screen on;

Isq;;AveBias;;UWLS3Bias;;REzBias;;PPBias;;PPzBias;;UWLS3cov;;REzcov;;PPcov;;PPzcov;;UWLS3RMSE;;REzRMSE;;PPRMSE;;PPzRMSE;

screen off;

sum1=0;sum2=0;sum3=0;sum10=0;sum11=0;

MSE1=0;MSE2=0;MSE3=0;MSE4=0;

sum4=0;sum5=0;sum6=0;sum7=0;sum9=0;sum8=0;

cov1=0;cov4=0;cov2=0;cov3=0;

k=1;

do while (k<=rep);/* the number of replications*/

Seinv1={};Seinv2={};Seinv3={};Seinv4={};

t1={}; t2={};r={};t3={};

W1i={};Zf={};Se={};t4={};Se3={};

Ssq1={};Ssq3={};Sez={};

W2i={};W3i={};W4i={};

Ssq2={}; Ssq4={};

/* space holder 1*/

/* space holder 2*/

/* space holder 3*/

r2=5;r1=0;/* the number of research studies is the sum times 10*/

j=1;

do while (j<=r2);

i=1;

do while (i<=10);

PB=rndu(1,1);

if PB <= .5;

X2=rndn(ID[i],1)/2;

/* space holder 4*/

/* space holder 5*/

/* space holder 6*/

/*Bivariate Correlation using regressions' t-values */

X1=ones(ID[i],1);

Y=X1+X2+rndn(ID[i],1);

X=X2;

_olsres=1;

{ nam,m,b,stb,vc,std,sig,cx,rsq,resid,dbw } = ols(0,Y,X);

ti=b[2]/std[2];/*corr is conventional corr, corr for UWLS3*/

corr=ti/(ti^2+ID[i]-2)^.5;cor=ti/(ti^2+ID[i]+1)^.5;

b1=het[i]*rndn(1,1);/*adds random Het in terms of Cohen's d*/

d1=b1+2*corr/(1-corr^2)^.5;corr=d1/(d1^2+4)^.5;

d2=b1+2*cor/(1-cor^2)^.5;cor=d2/(d2^2+4)^.5;

Zfi=.5*ln((1+corr)/(1-corr));/* Fisher's z*/

r=r|corr;Zf=Zf|Zfi;

/* SEtest is the SE for testing whether corr=0*/

SEtest=sqrt((1-corr^2)/(ID[i]-2));

/* SEma is the conventional meta-analysis SE for corr's*/

SEma=(1-corr^2)/sqrt((ID[i]-1));

/* SE3 is the SE for UWLS+3*/

SE3=sqrt((1-cor^2)/(ID[i]+1));

Se=Se|SEtest;

/* SE4 is the SE for Fisher's z*/

SE4=1/sqrt(ID[i]-3);Sez=Sez|SE4;

t1i=corr/SEma;Seinv1i=1/SEma;

t2i=corr/SEtest;Seinv2i=1/SEtest;

Seinv1=Seinv1|Seinv1i;t1=t1|t1i;

Seinv2=Seinv2|Seinv2i;t2=t2|t2i;

t3i=cor/SE3;Seinv3i=1/SE3;

t4i=Zfi/SE4;Seinv4i=1/SE4;

Seinv3=Seinv3|Seinv3i;t3=t3|t3i;

Seinv4=Seinv4|Seinv4i;t4=t4|t4i;

W4ii=Seinv4i^2;

W4i=W4i|W4ii;

Ssq4=Ssq4|SE4^2;

/* The below program selects entirely for statistical significance */

else;

ti=0;

do until ti>=critt[i];

X2=rndn(ID[i],1)/2;

/*Bivariate Correlation using regressions' t-values */

/* space holder 4*/

/* space holder 5*/

/* space holder 6*/

X1=ones(ID[i],1);

Y=X1+X2+rndn(ID[i],1);

X=X2;

_olsres=1;

{ nam,m,b,stb,vc,std,sig,cx,rsq,resid,dbw } = ols(0,Y,X);

ti=b[2]/std[2];/*corr is conventional corr, corr for UWLS3*/

corr=ti/(ti^2+ID[i]-2)^.5;cor=ti/(ti^2+ID[i]+1)^.5;

b1=het[i]*rndn(1,1);/*adds random Het in terms of Cohen's d*/

d1=b1+2*corr/(1-corr^2)^.5;corr=d1/(d1^2+4)^.5;

d2=b1+2*cor/(1-cor^2)^.5;cor=d2/(d2^2+4)^.5;

Zfi=.5*ln((1+corr)/(1-corr));/* Fisher's z*/

/* SEtest is the SE for testing whether corr=0*/

SEtest=sqrt((1-corr^2)/(ID[i]-2));

ti=corr/SEtest;

endo;

r=r|corr;Zf=Zf|Zfi;

/* SEma is the conventional meta-analysis SE for corr's*/

SEma=(1-corr^2)/sqrt((ID[i]-1));

/* SE3 is the SE for UWLS+3*/

SE3=sqrt((1-cor^2)/(ID[i]+1));

Se=Se|SE3;

/* SE4 is the SE for Fisher's z*/

SE4=1/sqrt(ID[i]-3);Sez=Sez|SE4;

t1i=corr/SEma;Seinv1i=1/SEma;

t2i=corr/SEtest;Seinv2i=1/SEtest;

Seinv1=Seinv1|Seinv1i;t1=t1|t1i;

Seinv2=Seinv2|Seinv2i;t2=t2|t2i;

t3i=cor/SE3;Seinv3i=1/SE3;

t4i=Zfi/SE4;Seinv4i=1/SE4;

Seinv3=Seinv3|Seinv3i;t3=t3|t3i;

Seinv4=Seinv4|Seinv4i;t4=t4|t4i;

W4ii=Seinv4i^2;

W4i=W4i|W4ii;

Ssq4=Ssq4|SE4^2;

endif;

i=i+1;

endo;

j=j+1;

endo;

rr=10*r2;

z1=ones(rr,1);n=W4i+(3*z1);

ave=r'*z1/rr; sum3=sum3+ave;

sumW4=W4i'*z1;

/* The below program calculates UWLS+3 & UWLS1 for I-sq */

bb1=inv(Seinv3'*Seinv3)*Seinv3't3;sum1=sum1+bb1;

s2wls=(t3-Seinv3*bb1)'*(t3-Seinv3*bb1)/(rr-1);

WLSVAR1=s2wls*inv(Seinv3'*Seinv3);MSE3=MSE3+(bb1[1]-sqrt(1/5))^2;

UL1=bb1[1]+2.01*sqrt(WLSVAR1);

LL1=bb1[1]-2.01*sqrt(WLSVAR1);

if LL1<=sqrt(1/5) and UL1>=sqrt(1/5);

cov3=cov3+1;

endif;

bb2=inv(Seinv1'*Seinv1)*Seinv1't1;

s2wls2=(t1-Seinv1*bb2)'*(t1-Seinv1*bb2)/(rr-1);

WLSVAR2=s2wls2*inv(Seinv1'*Seinv1);

I2=(s2wls2-1)/s2wls2;

if I2<0;I2=0;endif;sum8=sum8+I2;

/*Calculates Hunter&Schmidt--see H&S(1990) &Field(2001)*/

HS=(n'*r)/(z1'*n);/*Calculates Hunter&Schmidt MA*/

PP=HS;

VarHS=(n'*(r-HS*z1)^2)/(z1'n);

SEHS=sqrt((VarHS)/rr);/*Calculates Hunter&Schmidt's MA's SE*/

PPse=SEHS;

UL2=PP+1.96*PPse;

LL2=PP-1.96*PPse;

if LL2<=sqrt(1/5) and UL2>=sqrt(1/5);

cov2=cov2+1;

endif;

sum2=sum2+PP;MSE2=MSE2+(PP-sqrt(1/5))^2;

Wave4=sumW4/rr;/*see Eq (5.3) in Sutton et al (2000) */

S2w4=(t4'*z1-rr*Wave4^2)/(rr-1);/*Eq (5.4)*/

U=(rr-1)*(Wave4-S2w4/(rr*Wave4));/*Eq (5.5)*/

Q=(W4i'Zf^2)-(Zf'*W4i)^2/sumW4;/*Eq (3.2)*/

BSvar=(Q-rr+1)/U;/*Eq (5.6)*/

if Q<=rr-1;

BSvar=0;

endif;

iBS=1; weight={};

do while (iBS<=rr);

weighti=1/(BSvar+Ssq4[iBS]);/*Eq(5.7)*/

weight=weight|weighti;

iBS=iBS+1;

endo;

sumWeight1=weight'*z1;

REWA1=(Zf'*weight)/sumWeight1; /*Eq(5.8)*/

VARRE=1/sumWeight1;

ulr=REWA1+1.96*sqrt(VARRE);ulr=((exp(2*ulr)-1)/(exp(2*ulr)+1));

llr=REWA1-1.96*sqrt(VARRE);llr=((exp(2*llr)-1)/(exp(2*llr)+1));

if llr<=sqrt(1/5) and ulr>=sqrt(1/5);

cov1=cov1+1;

endif;

REzCorr=(exp(2*REWA1)-1)/(exp(2*REWA1)+1);

sum5=sum5+REzCorr;MSE1=MSE1+(REzCorr-sqrt(1/5))^2;

/*Calculates PET-PEESE using Fisher's z truncated at zero*/

zz=Seinv4~Sez;bb5=inv(zz'*zz)*zz't4;/*PEESE*/

PEESEzS2=(t4-zz*bb5)'*(t4-zz*bb5)/(rr-2);

PEESEzVAR=PEESEzS2*inv(zz'*zz);

zz=z1~Seinv4;bb6=inv(zz'*zz)*zz't4;/*PET*/

/*PET variance*/

PETzS2=(t4-zz*bb6)'*(t4-zz*bb6)/(rr-2);

PETVARz=PETzS2*inv(zz'zz);PETzSE=sqrt(PETVARz[2,2]);

teff=bb6[2]/PETzSE;PPz=bb6[2];PPsez=PETzSE;

if teff>=1.299;

PPz=bb5[1];PPsez=sqrt(PEESEzVAR[1,1]);

endif;

if PPz<0;PPz=0;endif;

PPcorr=(exp(2*PPz)-1)/(exp(2*PPz)+1);

UL3=PPz+2.008*PPsez;UL3=((exp(2*UL3)-1)/(exp(2*UL3)+1));

LL3=PPz-2.008*PPsez;LL3=((exp(2*LL3)-1)/(exp(2*LL3)+1));

if LL3<=sqrt(1/5) and UL3>=sqrt(1/5);

cov4=cov4+1;

endif;

sum4=sum4+PPcorr;MSE4=MSE4+(PPcorr-sqrt(1/5))^2;

k=k+1;

endo;

UWLS3Bias=(sum1/rep)-sqrt(1/5);UWLS3cov=cov3/rep;

REzBias=(sum5/rep)-sqrt(1/5);REzcov=cov1/rep;Isq=sum8/rep;

PPBias=(sum2/rep)-sqrt(1/5);PPcov=cov2/rep;

PPzBias=(sum4/rep)-sqrt(1/5);PPzcov=cov4/rep;

UWLS3RMSE=(MSE3/rep)^.5;PPzRMSE=(MSE4/rep)^.5;

REzRMSE=(MSE1/rep)^.5;PPRMSE=(MSE2/rep)^.5;

AveBias=(sum3/rep)-sqrt(1/5);

screen on;

Isq;;AveBias;;UWLS3Bias;;REzBias;;PPBias;;PPzBias;;UWLS3cov;;REzcov;;PPcov;;PPzcov;;UWLS3RMSE;;REzRMSE;;PPRMSE;;PPzRMSE;

screen off;

sum1=0;sum2=0;sum3=0;sum10=0;sum11=0;

MSE1=0;MSE2=0;MSE3=0;MSE4=0;

sum4=0;sum5=0;sum6=0;sum7=0;sum9=0;sum8=0;

cov1=0;cov4=0;cov2=0;cov3=0;

k=1;

do while (k<=rep);/* the number of replications*/

Seinv1={};Seinv2={};Seinv3={};Seinv4={};

t1={}; t2={};r={};t3={};

W1i={};Zf={};Se={};t4={};Se3={};

Ssq1={};Ssq3={};Sez={};

W2i={};W3i={};W4i={};

Ssq2={}; Ssq4={};

/* space holder 1*/

/* space holder 2*/

/* space holder 3*/

r2=5;r1=0;/* the number of research studies is the sum times 10*/

j=1;

do while (j<=r2);

i=1;

do while (i<=10);

PB=rndu(1,1);

if PB <= .5;

X2=rndn(ID[i],1)/4;

/* space holder 4*/

/* space holder 5*/

/* space holder 6*/

/*Bivariate Correlation using regressions' t-values */

X1=ones(ID[i],1);

Y=X1+X2+rndn(ID[i],1);

X=X2;

_olsres=1;

{ nam,m,b,stb,vc,std,sig,cx,rsq,resid,dbw } = ols(0,Y,X);

ti=b[2]/std[2];/*corr is conventional corr, corr for UWLS3*/

corr=ti/(ti^2+ID[i]-2)^.5;cor=ti/(ti^2+ID[i]+1)^.5;

b1=het[i]*rndn(1,1);/*adds random Het in terms of Cohen's d*/

d1=b1+2*corr/(1-corr^2)^.5;corr=d1/(d1^2+4)^.5;

d2=b1+2*cor/(1-cor^2)^.5;cor=d2/(d2^2+4)^.5;

Zfi=.5*ln((1+corr)/(1-corr));/* Fisher's z*/

r=r|corr;Zf=Zf|Zfi;

/* SEtest is the SE for testing whether corr=0*/

SEtest=sqrt((1-corr^2)/(ID[i]-2));

/* SEma is the conventional meta-analysis SE for corr's*/

SEma=(1-corr^2)/sqrt((ID[i]-1));

/* SE3 is the SE for UWLS+3*/

SE3=sqrt((1-cor^2)/(ID[i]+1));

Se=Se|SEtest;

/* SE4 is the SE for Fisher's z*/

SE4=1/sqrt(ID[i]-3);Sez=Sez|SE4;

t1i=corr/SEma;Seinv1i=1/SEma;

t2i=corr/SEtest;Seinv2i=1/SEtest;

Seinv1=Seinv1|Seinv1i;t1=t1|t1i;

Seinv2=Seinv2|Seinv2i;t2=t2|t2i;

t3i=cor/SE3;Seinv3i=1/SE3;

t4i=Zfi/SE4;Seinv4i=1/SE4;

Seinv3=Seinv3|Seinv3i;t3=t3|t3i;

Seinv4=Seinv4|Seinv4i;t4=t4|t4i;

W4ii=Seinv4i^2;

W4i=W4i|W4ii;

Ssq4=Ssq4|SE4^2;

/* The below program selects entirely for statistical significance */

else;

ti=0;

do until ti>=critt[i];

X2=rndn(ID[i],1)/4;

/*Bivariate Correlation using regressions' t-values */

/* space holder 4*/

/* space holder 5*/

/* space holder 6*/

X1=ones(ID[i],1);

Y=X1+X2+rndn(ID[i],1);

X=X2;

_olsres=1;

{ nam,m,b,stb,vc,std,sig,cx,rsq,resid,dbw } = ols(0,Y,X);

ti=b[2]/std[2];/*corr is conventional corr, corr for UWLS3*/

corr=ti/(ti^2+ID[i]-2)^.5;cor=ti/(ti^2+ID[i]+1)^.5;

b1=het[i]*rndn(1,1);/*adds random Het in terms of Cohen's d*/

d1=b1+2*corr/(1-corr^2)^.5;corr=d1/(d1^2+4)^.5;

d2=b1+2*cor/(1-cor^2)^.5;cor=d2/(d2^2+4)^.5;

Zfi=.5*ln((1+corr)/(1-corr));/* Fisher's z*/

/* SEtest is the SE for testing whether corr=0*/

SEtest=sqrt((1-corr^2)/(ID[i]-2));

ti=corr/SEtest;

endo;

r=r|corr;Zf=Zf|Zfi;

/* SEma is the conventional meta-analysis SE for corr's*/

SEma=(1-corr^2)/sqrt((ID[i]-1));

/* SE3 is the SE for UWLS+3*/

SE3=sqrt((1-cor^2)/(ID[i]+1));

Se=Se|SE3;

/* SE4 is the SE for Fisher's z*/

SE4=1/sqrt(ID[i]-3);Sez=Sez|SE4;

t1i=corr/SEma;Seinv1i=1/SEma;

t2i=corr/SEtest;Seinv2i=1/SEtest;

Seinv1=Seinv1|Seinv1i;t1=t1|t1i;

Seinv2=Seinv2|Seinv2i;t2=t2|t2i;

t3i=cor/SE3;Seinv3i=1/SE3;

t4i=Zfi/SE4;Seinv4i=1/SE4;

Seinv3=Seinv3|Seinv3i;t3=t3|t3i;

Seinv4=Seinv4|Seinv4i;t4=t4|t4i;

W4ii=Seinv4i^2;

W4i=W4i|W4ii;

Ssq4=Ssq4|SE4^2;

endif;

i=i+1;

endo;

j=j+1;

endo;

rr=10*r2;

z1=ones(rr,1);n=W4i+(3*z1);

ave=r'*z1/rr; sum3=sum3+ave;

sumW4=W4i'*z1;

/* The below program calculates UWLS+3 & UWLS1 for I-sq */

bb1=inv(Seinv3'*Seinv3)*Seinv3't3;sum1=sum1+bb1;

s2wls=(t3-Seinv3*bb1)'*(t3-Seinv3*bb1)/(rr-1);

WLSVAR1=s2wls*inv(Seinv3'*Seinv3);MSE3=MSE3+(bb1[1]-sqrt(1/17))^2;

UL1=bb1[1]+2.01*sqrt(WLSVAR1);

LL1=bb1[1]-2.01*sqrt(WLSVAR1);

if LL1<=sqrt(1/17) and UL1>=sqrt(1/17);

cov3=cov3+1;

endif;

bb2=inv(Seinv1'*Seinv1)*Seinv1't1;

s2wls2=(t1-Seinv1*bb2)'*(t1-Seinv1*bb2)/(rr-1);

WLSVAR2=s2wls2*inv(Seinv1'*Seinv1);

I2=(s2wls2-1)/s2wls2;

if I2<0;I2=0;endif;sum8=sum8+I2;

/*Calculates Hunter&Schmidt--see H&S(1990) &Field(2001)*/

HS=(n'*r)/(z1'*n);/*Calculates Hunter&Schmidt MA*/

PP=HS;

VarHS=(n'*(r-HS*z1)^2)/(z1'n);

SEHS=sqrt((VarHS)/rr);/*Calculates Hunter&Schmidt's MA's SE*/

PPse=SEHS;

UL2=PP+1.96*PPse;

LL2=PP-1.96*PPse;

if LL2<=sqrt(1/17) and UL2>=sqrt(1/17);

cov2=cov2+1;

endif;

sum2=sum2+PP;MSE2=MSE2+(PP-sqrt(1/17))^2;

Wave4=sumW4/rr;/*see Eq (5.3) in Sutton et al (2000) */

S2w4=(t4'*z1-rr*Wave4^2)/(rr-1);/*Eq (5.4)*/

U=(rr-1)*(Wave4-S2w4/(rr*Wave4));/*Eq (5.5)*/

Q=(W4i'Zf^2)-(Zf'*W4i)^2/sumW4;/*Eq (3.2)*/

BSvar=(Q-rr+1)/U;/*Eq (5.6)*/

if Q<=rr-1;

BSvar=0;

endif;

iBS=1; weight={};

do while (iBS<=rr);

weighti=1/(BSvar+Ssq4[iBS]);/*Eq(5.7)*/

weight=weight|weighti;

iBS=iBS+1;

endo;

sumWeight1=weight'*z1;

REWA1=(Zf'*weight)/sumWeight1; /*Eq(5.8)*/

VARRE=1/sumWeight1;

ulr=REWA1+1.96*sqrt(VARRE);ulr=((exp(2*ulr)-1)/(exp(2*ulr)+1));

llr=REWA1-1.96*sqrt(VARRE);llr=((exp(2*llr)-1)/(exp(2*llr)+1));

if llr<=sqrt(1/17) and ulr>=sqrt(1/17);

cov1=cov1+1;

endif;

REzCorr=(exp(2*REWA1)-1)/(exp(2*REWA1)+1);

sum5=sum5+REzCorr;MSE1=MSE1+(REzCorr-sqrt(1/17))^2;

/*Calculates PET-PEESE using Fisher's z truncated at zero*/

zz=Seinv4~Sez;bb5=inv(zz'*zz)*zz't4;/*PEESE*/

PEESEzS2=(t4-zz*bb5)'*(t4-zz*bb5)/(rr-2);

PEESEzVAR=PEESEzS2*inv(zz'*zz);

zz=z1~Seinv4;bb6=inv(zz'*zz)*zz't4;/*PET*/

/*PET variance*/

PETzS2=(t4-zz*bb6)'*(t4-zz*bb6)/(rr-2);

PETVARz=PETzS2*inv(zz'zz);PETzSE=sqrt(PETVARz[2,2]);

teff=bb6[2]/PETzSE;PPz=bb6[2];PPsez=PETzSE;

if teff>=1.299;

PPz=bb5[1];PPsez=sqrt(PEESEzVAR[1,1]);

endif;

if PPz<0;PPz=0;endif;

PPcorr=(exp(2*PPz)-1)/(exp(2*PPz)+1);

UL3=PPz+2.008*PPsez;UL3=((exp(2*UL3)-1)/(exp(2*UL3)+1));

LL3=PPz-2.008*PPsez;LL3=((exp(2*LL3)-1)/(exp(2*LL3)+1));

if LL3<=sqrt(1/17) and UL3>=sqrt(1/17);

cov4=cov4+1;

endif;

sum4=sum4+PPcorr;MSE4=MSE4+(PPcorr-sqrt(1/17))^2;

k=k+1;

endo;

UWLS3Bias=(sum1/rep)-sqrt(1/17);UWLS3cov=cov3/rep;

REzBias=(sum5/rep)-sqrt(1/17);REzcov=cov1/rep;Isq=sum8/rep;

PPBias=(sum2/rep)-sqrt(1/17);PPcov=cov2/rep;

PPzBias=(sum4/rep)-sqrt(1/17);PPzcov=cov4/rep;

UWLS3RMSE=(MSE3/rep)^.5;PPzRMSE=(MSE4/rep)^.5;

REzRMSE=(MSE1/rep)^.5;PPRMSE=(MSE2/rep)^.5;

AveBias=(sum3/rep)-sqrt(1/17);

screen on;

Isq;;AveBias;;UWLS3Bias;;REzBias;;PPBias;;PPzBias;;UWLS3cov;;REzcov;;PPcov;;PPzcov;;UWLS3RMSE;;REzRMSE;;PPRMSE;;PPzRMSE;

screen off;

sum1=0;sum2=0;sum3=0;sum10=0;sum11=0;

MSE1=0;MSE2=0;MSE3=0;MSE4=0;

sum4=0;sum5=0;sum6=0;sum7=0;sum9=0;sum8=0;

cov1=0;cov4=0;cov2=0;cov3=0;

k=1;

do while (k<=rep);/* the number of replications*/

Seinv1={};Seinv2={};Seinv3={};Seinv4={};

t1={}; t2={};r={};t3={};

W1i={};Zf={};Se={};t4={};Se3={};

Ssq1={};Ssq3={};Sez={};

W2i={};W3i={};W4i={};

Ssq2={}; Ssq4={};

/* space holder 1*/

/* space holder 2*/

/* space holder 3*/

r2=5;r1=0;/* the number of research studies is the sum times 10*/

j=1;

do while (j<=r2);

i=1;

do while (i<=10);

PB=rndu(1,1);

if PB <= .5;

X2=rndn(ID[i],1)/9;

/* space holder 4*/

/* space holder 5*/

/* space holder 6*/

/*Bivariate Correlation using regressions' t-values */

X1=ones(ID[i],1);

Y=X1+X2+rndn(ID[i],1);

X=X2;

_olsres=1;

{ nam,m,b,stb,vc,std,sig,cx,rsq,resid,dbw } = ols(0,Y,X);

ti=b[2]/std[2];/*corr is conventional corr, corr for UWLS3*/

corr=ti/(ti^2+ID[i]-2)^.5;cor=ti/(ti^2+ID[i]+1)^.5;

b1=het[i]*rndn(1,1);/*adds random Het in terms of Cohen's d*/

d1=b1+2*corr/(1-corr^2)^.5;corr=d1/(d1^2+4)^.5;

d2=b1+2*cor/(1-cor^2)^.5;cor=d2/(d2^2+4)^.5;

Zfi=.5*ln((1+corr)/(1-corr));/* Fisher's z*/

r=r|corr;Zf=Zf|Zfi;

/* SEtest is the SE for testing whether corr=0*/

SEtest=sqrt((1-corr^2)/(ID[i]-2));

/* SEma is the conventional meta-analysis SE for corr's*/

SEma=(1-corr^2)/sqrt((ID[i]-1));

/* SE3 is the SE for UWLS+3*/

SE3=sqrt((1-cor^2)/(ID[i]+1));

Se=Se|SEtest;

/* SE4 is the SE for Fisher's z*/

SE4=1/sqrt(ID[i]-3);Sez=Sez|SE4;

t1i=corr/SEma;Seinv1i=1/SEma;

t2i=corr/SEtest;Seinv2i=1/SEtest;

Seinv1=Seinv1|Seinv1i;t1=t1|t1i;

Seinv2=Seinv2|Seinv2i;t2=t2|t2i;

t3i=cor/SE3;Seinv3i=1/SE3;

t4i=Zfi/SE4;Seinv4i=1/SE4;

Seinv3=Seinv3|Seinv3i;t3=t3|t3i;

Seinv4=Seinv4|Seinv4i;t4=t4|t4i;

W4ii=Seinv4i^2;

W4i=W4i|W4ii;

Ssq4=Ssq4|SE4^2;

/* The below program selects entirely for statistical significance */

else;

ti=0;

do until ti>=critt[i];

X2=rndn(ID[i],1)/9;

/*Bivariate Correlation using regressions' t-values */

/* space holder 4*/

/* space holder 5*/

/* space holder 6*/

X1=ones(ID[i],1);

Y=X1+X2+rndn(ID[i],1);

X=X2;

_olsres=1;

{ nam,m,b,stb,vc,std,sig,cx,rsq,resid,dbw } = ols(0,Y,X);

ti=b[2]/std[2];/*corr is conventional corr, corr for UWLS3*/

corr=ti/(ti^2+ID[i]-2)^.5;cor=ti/(ti^2+ID[i]+1)^.5;

b1=het[i]*rndn(1,1);/*adds random Het in terms of Cohen's d*/

d1=b1+2*corr/(1-corr^2)^.5;corr=d1/(d1^2+4)^.5;

d2=b1+2*cor/(1-cor^2)^.5;cor=d2/(d2^2+4)^.5;

Zfi=.5*ln((1+corr)/(1-corr));/* Fisher's z*/

/* SEtest is the SE for testing whether corr=0*/

SEtest=sqrt((1-corr^2)/(ID[i]-2));

ti=corr/SEtest;

endo;

r=r|corr;Zf=Zf|Zfi;

/* SEma is the conventional meta-analysis SE for corr's*/

SEma=(1-corr^2)/sqrt((ID[i]-1));

/* SE3 is the SE for UWLS+3*/

SE3=sqrt((1-cor^2)/(ID[i]+1));

Se=Se|SE3;

/* SE4 is the SE for Fisher's z*/

SE4=1/sqrt(ID[i]-3);Sez=Sez|SE4;

t1i=corr/SEma;Seinv1i=1/SEma;

t2i=corr/SEtest;Seinv2i=1/SEtest;

Seinv1=Seinv1|Seinv1i;t1=t1|t1i;

Seinv2=Seinv2|Seinv2i;t2=t2|t2i;

t3i=cor/SE3;Seinv3i=1/SE3;

t4i=Zfi/SE4;Seinv4i=1/SE4;

Seinv3=Seinv3|Seinv3i;t3=t3|t3i;

Seinv4=Seinv4|Seinv4i;t4=t4|t4i;

W4ii=Seinv4i^2;

W4i=W4i|W4ii;

Ssq4=Ssq4|SE4^2;

endif;

i=i+1;

endo;

j=j+1;

endo;

rr=10*r2;

z1=ones(rr,1);n=W4i+(3*z1);

ave=r'*z1/rr; sum3=sum3+ave;

sumW4=W4i'*z1;

/* The below program calculates UWLS+3 & UWLS1 for I-sq */

bb1=inv(Seinv3'*Seinv3)*Seinv3't3;sum1=sum1+bb1;

s2wls=(t3-Seinv3*bb1)'*(t3-Seinv3*bb1)/(rr-1);

WLSVAR1=s2wls*inv(Seinv3'*Seinv3);MSE3=MSE3+(bb1[1]-sqrt(1/82))^2;

UL1=bb1[1]+2.01*sqrt(WLSVAR1);

LL1=bb1[1]-2.01*sqrt(WLSVAR1);

if LL1<=sqrt(1/82) and UL1>=sqrt(1/82);

cov3=cov3+1;

endif;

bb2=inv(Seinv1'*Seinv1)*Seinv1't1;

s2wls2=(t1-Seinv1*bb2)'*(t1-Seinv1*bb2)/(rr-1);

WLSVAR2=s2wls2*inv(Seinv1'*Seinv1);

I2=(s2wls2-1)/s2wls2;

if I2<0;I2=0;endif;sum8=sum8+I2;

/*Calculates Hunter&Schmidt--see H&S(1990) &Field(2001)*/

HS=(n'*r)/(z1'*n);/*Calculates Hunter&Schmidt MA*/

PP=HS;

VarHS=(n'*(r-HS*z1)^2)/(z1'n);

SEHS=sqrt((VarHS)/rr);/*Calculates Hunter&Schmidt's MA's SE*/

PPse=SEHS;

UL2=PP+1.96*PPse;

LL2=PP-1.96*PPse;

if LL2<=sqrt(1/82) and UL2>=sqrt(1/82);

cov2=cov2+1;

endif;

sum2=sum2+PP;MSE2=MSE2+(PP-sqrt(1/82))^2;

Wave4=sumW4/rr;/*see Eq (5.3) in Sutton et al (2000) */

S2w4=(t4'*z1-rr*Wave4^2)/(rr-1);/*Eq (5.4)*/

U=(rr-1)*(Wave4-S2w4/(rr*Wave4));/*Eq (5.5)*/

Q=(W4i'Zf^2)-(Zf'*W4i)^2/sumW4;/*Eq (3.2)*/

BSvar=(Q-rr+1)/U;/*Eq (5.6)*/

if Q<=rr-1;

BSvar=0;

endif;

iBS=1; weight={};

do while (iBS<=rr);

weighti=1/(BSvar+Ssq4[iBS]);/*Eq(5.7)*/

weight=weight|weighti;

iBS=iBS+1;

endo;

sumWeight1=weight'*z1;

REWA1=(Zf'*weight)/sumWeight1; /*Eq(5.8)*/

VARRE=1/sumWeight1;

ulr=REWA1+1.96*sqrt(VARRE);ulr=((exp(2*ulr)-1)/(exp(2*ulr)+1));

llr=REWA1-1.96*sqrt(VARRE);llr=((exp(2*llr)-1)/(exp(2*llr)+1));

if llr<=sqrt(1/82) and ulr>=sqrt(1/82);

cov1=cov1+1;

endif;

REzCorr=(exp(2*REWA1)-1)/(exp(2*REWA1)+1);

sum5=sum5+REzCorr;MSE1=MSE1+(REzCorr-sqrt(1/82))^2;

/*Calculates PET-PEESE using Fisher's z truncated at zero*/

zz=Seinv4~Sez;bb5=inv(zz'*zz)*zz't4;/*PEESE*/

PEESEzS2=(t4-zz*bb5)'*(t4-zz*bb5)/(rr-2);

PEESEzVAR=PEESEzS2*inv(zz'*zz);

zz=z1~Seinv4;bb6=inv(zz'*zz)*zz't4;/*PET*/

/*PET variance*/

PETzS2=(t4-zz*bb6)'*(t4-zz*bb6)/(rr-2);

PETVARz=PETzS2*inv(zz'zz);PETzSE=sqrt(PETVARz[2,2]);

teff=bb6[2]/PETzSE;PPz=bb6[2];PPsez=PETzSE;

if teff>=1.299;

PPz=bb5[1];PPsez=sqrt(PEESEzVAR[1,1]);

endif;

if PPz<0;PPz=0;endif;

PPcorr=(exp(2*PPz)-1)/(exp(2*PPz)+1);

UL3=PPz+2.008*PPsez;UL3=((exp(2*UL3)-1)/(exp(2*UL3)+1));

LL3=PPz-2.008*PPsez;LL3=((exp(2*LL3)-1)/(exp(2*LL3)+1));

if LL3<=sqrt(1/82) and UL3>=sqrt(1/82);

cov4=cov4+1;

endif;

sum4=sum4+PPcorr;MSE4=MSE4+(PPcorr-sqrt(1/82))^2;

k=k+1;

endo;

UWLS3Bias=(sum1/rep)-sqrt(1/82);UWLS3cov=cov3/rep;

REzBias=(sum5/rep)-sqrt(1/82);REzcov=cov1/rep;Isq=sum8/rep;

PPBias=(sum2/rep)-sqrt(1/82);PPcov=cov2/rep;

PPzBias=(sum4/rep)-sqrt(1/82);PPzcov=cov4/rep;

UWLS3RMSE=(MSE3/rep)^.5;PPzRMSE=(MSE4/rep)^.5;

REzRMSE=(MSE1/rep)^.5;PPRMSE=(MSE2/rep)^.5;

AveBias=(sum3/rep)-sqrt(1/82);

screen on;

Isq;;AveBias;;UWLS3Bias;;REzBias;;PPBias;;PPzBias;;UWLS3cov;;REzcov;;PPcov;;PPzcov;;UWLS3RMSE;;REzRMSE;;PPRMSE;;PPzRMSE;

screen off;
